# Acknowledgment to the Reviewers of *Cells* in 2022

**DOI:** 10.3390/cells12030375

**Published:** 2023-01-19

**Authors:** Cells Editorial Office

**Affiliations:** MDPI AG, St. Alban-Anlage 66, 4052 Basel, Switzerland

High-quality academic publishing is built on rigorous peer review. *Cells* was able to uphold its high standards for published papers due to the outstanding efforts of our reviewers. Thanks to the efforts of our reviewers in 2022, the median time to first decision was 16 days and the median time to publication was 42 days. Regardless of whether the articles they examined were ultimately published, the editors would like to express their appreciation and thank the following reviewers for the time and dedication that they have shown *Cells*:
Ababei, Daniela CarmenLiu, ManAbate, GiuliaLiu, PengdaAbate, MarioLiu, QinghuaAbbas, Zulfiqarali G.Liu, SaifeiAbbaspour-Ravasjani, SoheilLiu, Shing-HwaAbdallah, AliLiu, ShubingAbdeen, AhmedLiu, SumeiAbdel Latef, Arafat Abdel HamedLiu, TaoAbdelgied, MohamedLiu, TianAbd-Elhafeez, Hanan HassanLiu, TingAbdelhamid, LeilaLiu, TongbaoAbdellatif, AhmedLiu, Wen-ChunAbdelmohsen, KotbLiu, XiamingAbdolahinia, Elaheh DalirLiu, XiaoguangAbdul, Hamid NazefahLiu, XiaomengAbdul, Momin SiddiqueLiu, YanAbdul, Rahman AhmadLiu, YanqingAbdullah, Nurul AsmaLiu, YeAbe, MasanoriLiu, YongqingAbedini, Nassab RoozbehLiu, Yong-YuAbid, MuhammadLiu, YuAbken, HinrichLiu, YuruAboouf, Mostafa A.Liu, ZezhongAboubakar, FrankLiu, ZhiyongAbramowitz, Lara K.Liu, ZhonghuaÅbrink, MagnusLiu, Zhong-huaAbu-Gharbieh, EmanLiu, ZuqiangAbukhalil, Mohammad H.Liverani, ChiaraAbu-Shah, EnasLivesey, Matthew RobertAbuyassin, Bisher H.Liwo, Jozef AdamAcar, NurayLjubimov, AlexanderAccardi, LuisaLlama-Palacios, AranchaAcevedo, Juan JoseLlano, ElenaAcharya, SuchismitaLlansola, MartaAcosta, Tomás JavierLleo, MarAd’hiah, AliLlop, MartaAdachi, YasushiLlopis, Jose LaparraÁdám, Attila L.Llorens, JordiAdam, KostiLo Giudice, RobertoAdam, Stephen A.Lo Gullo, AlbertoAdamakis, Ioannis-DimosthenisLo Nigro, LucaAdameyko, IgorLo, Chih HungAdams, Michelle M.Lo, Re OrianaAdams, VolkerLobachevsky, PavelAdell, TeresaLoffredo, StefaniaAdem, AbduLoftås, Per I.Adhikari, ManishLogarinho, ElsaAdil, MaaroufLogoyda, Liliya S.Adjobimey, TomabuLoh, Y. PengAdlung, LorenzLohan, SandeepAdolf-Bryfogle, JaredLoiola, Rodrigo A.Afonnikov, Dimitry ArkadievichLok, Josephine M.Afzal, ObaidLolicato, MarcoAfzal, SheryarLombardo, GiorgioAggeli, ConstantinaLombó, MartaAghayan, HamidrezaLondei, PaolaAgnoletti, DavideLone, Ajaz A.Agrawal, AshishLong, Sarah AliceAgrawal, Devendra K.Long, YongAgrawal, ShrutiLongato, Giovanna BarbariniAgrawal-Rajput, ReenaLongatto-Filho, AdhemarAgresta, FerdinandoLoo, ShiningAguado, EnriqueLopes, CarlaAgudo-Barriuso, MartaLopes, Ferreira MonicaAguilar‑Pontes, Maria VictoriaLopes-Pacheco, MiquéiasAhamad, ShahzaibLópez, Claudia S.Ahel, IvanLópez, Jesús AdriánAhluwalia, PankajLópez, JudithAhmad, AamirLopez, Michael A.Ahmad, AnasLópez-Bertoni, HernandoAhmad, KhurshidLópez-Camarillo, CésarAhmadian, ElhamLopez-Charcas, OsbaldoAhmed, AmerLópez-Hernández, Luz BereniceAhmed, EbtehalLópez-Iglesias, CarmenAhmed, Idris AdewaleLopez-Jornet, PiaAhmed, ShabbirLõpez-Pingarrõn, LauraAhmed, ZaheelLopez-Sanchez, CarmenAhn, Mok-RyeonLópez-Urrutia, EduardoAhrendsen, Jared T.LoPresti, PatriziaAhrenfeldt, Linda JuelLorentz, Axel C.Ahsan, NagibLorenzo, Gabriele DiAich, PalokLorenzo, JosephAire, Tom A.Lorenzo, ÓscarAisa, Haji A.Lorico, AurelioAit-El-Mokhtar, MohamedLospinoso, Severini LudovicaAjduk, AnnaLouis, FionaAkbar, NaveedLouis, Irina St.Akbar, Sheikh Mohammad FazleLow, WalterAkbaş, BirolLozach, Pierre-YvesAkbulut, SamiLoza-Coll, Mariano AndresAkeda, KojiLozano, ReymundoAkgül, BakiLu, HaiquanAkhade, Ajay SureshLu, HongAkimoto, SeijiLu, Hong S.Akiyama, HisashiLu, HuiAkrida, IoannaLu, HuimingAksak-Wąs, BoguszLu, JinqiuAksoy, Gündoğdu AslıLu, Joyce JeanAkundi, Ravi ShankarLu, Kang-leAkutsu, HidenoriLu, LiminAla, UgoLu, LingengAlaaeldin, RaniaLu, LixiaAlam, AfrozLu, LuAlam, Khan Md.Lu, ShaoyongAlam, Talha MahboobLu, Wei-YangAlbanesi, Joseph P.Lu, Wei-YuAlbarracín, Sonia L.Lu, YonggangAlberdi, ElenaLu, YujiaoAl-brakati, AshrafLuan, HaitaoAlbrecht, ElkeLuan, YiAlcalde, IgnacioLuarte, AlejandroAlcántara-Ortigoza, Miguel AngelLubecka-Gajewska, KatarzynaAlder, JanetLubet, Ronald A.Aldskogius, HakanLucà, FabianaAl-Dujaili, EmadLucas, SebastianAleanizy, FadilahLucas-Hahn, AndreaAlemán, IgnacioLucchetti, DonatellaAlepuz, PaulaLuchetti, FrancescaAlessandro, FurlanLuchini, ClaudioAlessenko, Alice V.Luci, CarmeloAlexandre, EscargueilLuciani, Giovanni BattistaAlfaidy, NadiaLucien-Matteoni, FabriceAlfatah, MohammadLuddi, AliceAlfieri, CarolineLudovico, PaulaAl-Hilal, Taslim AhmedLue, YanheAl-Hilaly, YoussraLuevano-Contreras, ClaudiaAli, AkhtarLugini, LuanaAli, AliLuhmann, Heiko J.Ali, Amira A.Luigi, De BellisAli, AsifLuís Silva, CatarinaAli, FatmaLuís, Ana LúciaAli, Hamed I.Luis, Pereira GuilhermeAli, HashimLuisa, BelluAli, Md KhademLuissint, Anny ClaudeAli, ReemLukacova, NadezdaAli, Syed AzmalLukas, Rimas V.Aliel, Vigano PugliesLukaszuk, KrzysztofAlipour, MahdiehLukens, JohnAlisi, AnnaLukyanov, Konstantin A.Alizargar, JavadLuna-Luna, MaríaAlkass, KanarLuo, ShiyuAlkhateeb, AbedalrhmanLuo, ShouqingAllahverdi, AbdollahLuo, Zhen-GeAllard-Chamard, HuguesLupia, EnricoAllegretti, Silmara MarquesLupiáñez, José AntonioAllen-Petersen, BrittanyLuscinskas, Francis William (Bill)Allmendinger, AndreaLussier, MarcAllsopp, RichardLuttge, ReginaAlmeida, ArmandoLutz, DavidAlmeida, Palha JoanaLutz, SusanneAl-Musawi, SharafaldinLuvoni, Gaia CeciliaAloisi, Anna MariaLuxton, G. W. GantAlonso, Miguel A.Lv, DeguanAlonso, SergioLyakhovitsky, AnnaAlpini, GianfrancoLyer, StefanAlquisiras-Burgos, IvánLyssek-Borón, AnitaAlshehri, MansoorŁyszkiewicz, MarcinAlsop, KathrynM’kacher, RadhiaAlster, PiotrMa, HongAltaf, Muhammad AhsanMa, JingAltamura, ClaudiaMa, LiangAltmann, FriedrichMa, NanAltrock, EvaMa, TongcuiAltun, ZekiyeMa, XiaokuangAltuna, MirenMa, XiaoxiaoAlula, KibromMa, XinliangAluri, JahnaviMa, YixuanAlvares, DayaneMaarsingh, HarmAlvarez, AngelMaass, KendraAlvarez, JavierMabasa, LawrenceAlvarez-Mercado, Ana IsabelMabbott, Neil AndrewÁlvarez-Rodríguez, ManuelMabeta, PeaceAlvarez-Silva, MarcioMacAulay, Valentine MoyaÁlvarez-Sola, GloriaMaccagnano, GiuseppeAlves, EduardoMaccagno, MassimoAlves, Marco G.Maccio, AntonioAlzahrani, FaisalMacDonald, Clinton C.Amador, Cristián A.MacDowell, Karina S.Amantea, DianaMach, Robert H.Amaral, AnaMachado, JulianoAmaro, AdrianoMachado-Neto, João AgostinhoAmasheh, SalahMachairas, NikolaosAmbatipudi, KiranMachlus, KellieAmber, Kyle T.Macias, Maria J.Ambrosio, MariaMacias-Segura, NoéAmdare, NitinMaciej, CieslaAmedeo, LonardoMaciver, SutherlandAmes, James B.Mackenzie, FrancescaAmé-Thomas, PatriciaMackrill, John J.Amico, GiandomenicoMacLean, JamesAmillis, SotirisMacNamara, Katherine C.Amin, AmrMacurek, LiborAmmirabile, MassimilianoMaddala, RupalathaAmodeo, Giuseppe FedericoMadeira Lucci, CarolinaAmor, MatthieuMadeira Nogueira, António JoséAmorim, ChristianiMadka, VenkateshwarAmrani, AbdelazizMadonna, Mary BethAmro, HanoraMadonna, RosalindaAn, Steven S.Madrid-Marina, VicenteAn, Yu AaronMądry, EdytaAnagnostopoulos, KonstantinosMadurga, RodrigoAnagnostouli, MariaMaduro, MorrisAnand, JayMaeda, KazuhikoAnastasiadou, EleniMaeda, RobertAnastasio, Albert ThomasMaeda, TadaoAnbazhagan, A. N.Maeda, TomojiAndersen, HenningMaeda, YoshiakiAndersen, Jens VeldeMaekawa, RyoAndersen, Joshua L.Maeng, SunghoAnderson, EthanMaestri, MarcelloAndersson Ørom, UlfMaffini, FaustoAndjelic, SofijaMaftei, George AlexandruAndl, Claudia D.Magagnoli, FedericaAndó, IstvánMagdinier, FrederiqueAndrás, IbolyaMager, DianaAndre, Alberto WeberMagerus, AudeAndrea, LeviMaggi, DavideAndreadis, SteliosMaggi, Leonard B.Andreatos, NikolaosMaggi, LorenzoAndreetta, FrancescaMaggiolini, MarcelloAndreoni, KennethMagnaudeix, AmandineAndrés, AntonioMagrassi, LorenzoAndres-Mach, MartaMagrì, AndreaAndreuzzi, EvaMaguy, AngeAndrianova, Nadezda V.Mahadevan, DarukaAndronov, Evgeny E.Mahajan, GautamAnelia, HorvathMahalingam, RaviAngel, Sergio O.Mahalingam, ShanmugaAngelastro, JamesMahapatra, SidharthAngelini, PaolaMahavadi, PoornimaAngelov, DoychinMahendra, MikhilaAngelova, Plamena R.Maher, Pamela A.Anger, MartinMahmood, KhalidAnghel, LarisaMahmood, SyedAnifandis, George-SpyrosMahmoud, AymanAniksztejn, LaurentMahmud, ShafiAnnamaria, AloisioMahrooz, AbdolkarimAnnels, NicolaMaia, ClaudioAnsari, MojtabaMaia, Fátima RaquelAnsari, PrawejMaier, Jeanette A. M.Ansari, Suraiya A.Mailleux, ArnaudAntiga, EmilianoMaiti, GuruAnto, Ruby JohnMaître, BlandineAntognelli, CinziaMaity, PritiprasannaAntón, Inés M.Maity, ShuvadeepAntoniali, GiuliaMajewska, Ania K.Antonin, WolframMajima, Hideyuki J.Antoniu, SabinaMajumder, SamarpanAntony, JustinMakarevitch, IrinaAnwar, MumtazMakarova, ElenaAnwar, SirajudheenMakena, MonishAoe, TomohikoMakridakis, ManousosAoki, JunMalakootian, MahshidAoudjit, FawziMalampati, SandeepAparecida, Rainho ClaudiaMalandish, AbbasAparicio-Trejo, Omar EmilianoMalapelle, UmbertoApostolova, NadezdaMalashicheva, Anna B.Aqdas, MohammadMalatesta, ManuelaArab, Hany HamdyMalavasi, FabioAragona, MariaMalavolta, MarcoAragonés, JuliánMalcangi, GiuseppinaAraki, ToshiyukiMalcher, AgnieszkaAraldi, Rodrigo PinheiroMalejczyk, JacekAramini, BeatriceMalek, AnastasiaAranda-Abreu, Gonzalo EmilianoMalewski, TadeuszAraneda, Oscar F.Malik, KafaitArata, ToshiakiMalik, RadekAraújo, Eugênio GonçalvesMalik, Rayaz A.Arav, AmirMalinova, DessiAravamudhan, AjaMalinowska, BarbaraArbab, Ali S.Malinzi, JosephArbeithuber, BarbaraMaljevic, SnezanaArcanjo, DanielMalkin, SergeyArce, Agustín L.Mallack, EricArciszewski, MarcinMallett, AndrewArcucci, AlessandroMallis, PanagiotisArdiles, AlvaroMalmström, AnnikaArditi, MosheMalook, SaifulArduini, ArduinoMalorni, WalterArefieva, TatianaMaltsev, VictorArellanes-Robledo, JaimeMalyshev, IgorArgyle, DavidMalysheva, OlgaAriani, AlaricoMamessier-Birnbaum, EmilieArias, JonathanMamun-Or-Rashid, A. N. M.Arias-Pulido, HugoMan, Si MingArif, Mian AbdurManavathi, BramanandamArif, TasleemMancinelli, RominaAringer, IdaMancinelli, RosaArisan, Elif DamlaMancini, AndreaArjunan, SridharMancini, MaicolArmakolas, AthanasiosMancuso, ElettraArmato, UbaldoMandal, AmritlalArmenis, IakovosMandal, ChanchalArndt, Patrick G.Mandal, Tapas K.Arnold, PhilippMandrich, LuigiArosarena, OneidaManenti, AntonioArosio, PaoloManera, ClementinaArranz, Amaia M.Manetti, MirkoArriagada, CésarManferdini, CristinaArroyo, MaríaManfredi, FrancescoArroyo-García, Luis EnriqueManfredi, Kirk P.Arslan, Mehmet EnesManfredi, MarcelloArtico, MarcoManganaro, LaraArtinger, KatharinaManganelli, ValeriaArufe, María C.Mangeot, PhilippeArumugam, MuthuMangiagalli, MarcoArũnas, RamanavičiusMangino, GiorgioArutjunyan, Alexander V.Mani, MuralidharanArvanitis, Demetrios A.Maniaci, AntoninoArzmi, Mohd HafizManian, MostafaAsa’ad, FarahMann, GiovanniAsada, KiyoshiMann, MellissaAsano, ShinjiMannelli, FrancescoAsao, HironobuMannherz, Hans GeorgAscenzioni, FiorentinaMannino, FedericaAseervatham, JayaManogaran, PrasathAshkavand, ZahraManon, Stéphen T.Ashrafian, FatemehManry, JeremyAshwell, Ken WilliamManterola, MarciaAsif, MuhammadManti, LorenzoAskjaer, PeterManya, HiroshiAsperti, MichelaManzano-López, JavierAsproni, PietroManzotti, GloriaAssfalg, MichaelMao, Chai-AnAssinck, PeggyMao, CunguiAssiri, AbdullahMao, XumingAtalay, ErayMao, YichengAten, SydneyMao, YoudongAthanassiou, PanagiotisMaoz, Ben M.Atif, Shaikh MuhammadMapoung, SariyaAtiomo, WilliamMapuskar, Kranti A.Atlante, AnnaMarabese, MirkoAtrash, ShebliMarampon, FrancescoAtsushi, KaidaMarangi, GiuseppeAttri, Kuldeep SinghMarangos, PetrosAttur, MukundanMarcato, PaolaAuburger, GeorgMarchal, Juan AlbertoAudrito, ValentinaMarchese, MariaAugusto, Danillo GardenalMarchesini, MaurizioAugusto, LeonardoMarchetti, LauraAumailley, MoniqueMarchetti, MoniaAustin, ShaneMarchini, TimoteoAvendaño, CarlosMarchisio, MarioAvidor-Reiss, TomerMarciniak, PawełÁvila-Flores, AntoniaMarco, Eva M.Ávila-Quezada, Graciela D.Marco, RasileAvilés-Trigueros, MarcelinoMarcoccia, DanieleAvitan-Hersh, EmilyMarcoli, ManuelaAvni, OrlyMarcolongo, PaolaAwasthi, DeepikaMarcucci, FabrizioAwasthi, SharadMarcuello, CarlosAwatade, NikhilMarcuzzo, StefaniaAxelson, HakanMarec, FrantišekAydin, MalikMaréchal, AlexandreAyroldi, Emira MariaMarei, HanyAyuso-Dominguez, José MaríaMarengo, BarbaraAzam, MaleehaMaret, WolfgangAzam, ShofiulMaretzky, ThorstenAzevedo, JorgeMargari, LuciaAzimzadeh, OmidMargolis, LeonidAziriova, SilviaMargulis, BorisAziz, FaisalMaria, Teresa ParraAziz, IrfanMarian, BrigitteAzkona, GarikoitzMariani, Louise-LaureAznar, SusanaMarigo, ValeriaAzpiazu, NataliaMarimani, Musa D.Azuma, Yasu-TakaMarina Elli, MarinaBabajani, AmirhesamMarinas-Pardo, LuisBabelova, AndreaMaringhini, SilvioBabenko, VladimirMarini, MarinaBábíčková, JankaMarino, AngelaBabu, Gopal J.Marino, BrunoBabuska, VaclavMarino, FabiolaBabwah, AndyMarino, JosephBachmann, MalteMarino, SilviaBacso, ZsoltMarionneau, CélineBadimon, Juan J.Mariotti, Francesca RomanaBaer, Patrick C.Mark, A. NakasoneBaez, Maria VeronicaMarkelic, MilicaBagam, PrathyushaMärklin, MelanieBagchi, SounakMarković, MarijaBagga, SumedhaMarkussen, LasseBagnoli, PaolaMarney, ChristinaBagyinszky, EvaMarone, GianniBahn, Jae HoonMarote, AnaBahri, RajiaMarques, André R. A.Bai, MeizhuMarqués, María CarmenBai, QifengMarques, Mariana GrokeBai, XiaowenMarquez-Molins, JoanBailly, ChristianMarracci, SilviaBaines, JoelMarriott, IanBaiula, MonicaMarsano, Renè MassimilianoBajguz, AndrzejMarshall, Sarah A.Bajoghli, BaubakMarston, Nicholas A.Bajwa, Vikramjit S.Martagón, Alexandro J.Bakacsy, LászlóMartel, CatherineBakaev, MaximMartens, SaschaBakchoul, TamamMartha, C. Rosales-HernándezBaker, Brent A.Martí, JoaquínBaker, Jonathan R.Martignetti, LoredanaBaker, Mark D.Martikainen, MiikaBaker, Martin J.Martikainen, RiikkaBaker, Michael E.Martin, DavyBakht, MartinMartin, FranckBakhti, MostafaMartin, Lee J.Baksh, ShairazMartín, MargaritaBal, NataliaMartín, Miguel A.Balaban, PavelMartin, NeilBalaguer, PatrickMartinelli, JonathanBalakathiresan, Nagaraja S.Martínez, Beamonte RobertoBalakirev, Evgeniy S.Martínez, ConstantinoBalakrishnan, AshaMartinez, SalvadorBalan, MurugabaskarMartínez-Costa, Oscar H.Balasubramanian, SureshkumarMartínez-Hackert, ErikBalázs, NémethMartínez-Iglesias, OlaiaBalbi, CarolinaMartínez-Martínez, ErnestoBaldanzi, GianlucaMartínez-Martínez, IreneBalderas, EnriqueMartinez-Moreno, FernandoBaldini, FrancescaMartínez-Valle, FernandoBaldridge, MeganMartini, AndreaBali, Zsolt KristofMartiniano, BelloBalieva, Flora N.Martín-Nieto, JoséBaljinnyam, BolormaaMartino, Carlos F.Balla, AndrásMartino, SabataBallesteros, DanielMartino, TamiBalligand, ThomasMartin-Requero, AngelesBallinger, MeganMartins, Ana D.Balog, BrianMartins, DanielBalogh, MihalyMartins, PauloBalsinde, JesúsMartins, TâniaBalzarotti, MonicaMartins-Marques, TaniaBamford, ConnorMartín-Vasallo, PabloBan, JelenaMartorana, AlessandroBanack, SandraMarty, CarolineBandopadhyay, RinaMarucho, MarceloBandyopadhyay, Bidhan C.Marullo, StefanoBanerjee, BodhisattwaMaruyama, JunkiBanerjee, DebabrataMaruyama, KeiBanerjee, ManishaMarwarha, GurdeepBanerjee, RajdeepMarx, ÍtalaBanerji, Christopher R. S.Marzano, Angelo ValerioBanerji, VershaMarziliano, NicolaBangru, SushantMarzioni, DanielaBaniahmad, AriaMas, EricBaniulyte, GabrieleMasafumi, MurataniBank, IlanMasamune, AtsushiBankapalli, KondalaraoMasarone, DanieleBano, BilqeesMashhadzadeh, Amin HamedBansal, Shyam BihariMasondo, NqobileBao, XiucongMassadi, Omar AlBaptista, PedroMassart, JulieBar, JuliaMassenzio, FrancescaBarabadi, ZahraMasserdotti, GiacomoBaraldo, MartinaMassieu, LourdesBaran, JarekMassimiani, MicolBaranauskiene, LinaMasson, PatrickBarancik, MiroslavMastropasqua, LeonardoBaranyi, UlrikeMasui, KentaBarata, JoãoMasunaga, Shin-ichiroBarauna, Valério GarroneMasutani, MitsukoBarbagallo, FedericaMasyuk, AnatoliyBarbalho, SandraMatějka, RomanBarbanti, PieroMateos, JesúsBarbasz, JakubMathada, Umesh TharehalliBarbato, ChristianMatharu, NavneetBarberis, MatteoMatias-Guiu, JorgeBarbi, JosephMatilde, TaddeiBarbieri, IsaiaMatiolli, CleversonBarbier-Torres, LucíaMatos, Ângelo P.Barbosa, Daniel JoséMatos, PauloBarcena, Maria LuisaMatou-Nasri, SabineBarchiesi, JulietaMatsas, RebeccaBarciszewska, Anna-MariaMatsubara, KeiichiBar-Haim, ErezMatsuda, FukoBarhoumi, TililiMatsuda, HisashiBarile, LucioMatsuda, MihoBarison, AndreaMatsuda, YoshikoBarisón, María JuliaMatsui, HirotakaBarja-Fidalgo, ChristinaMatsumoto, KazumasaBarletta, GiuseppeMatsuno, YusukeBarman, PijusMatsushima, AyamiBarman, SusmitaMatsuyama, ShigemiBarna, BalazsMatsuzaka, YasunariBarnea, Eytan R.Matt, StephanieBarodia, Sandeep KumarMattei, FabrizioBaron, ElmaMattei, VincenzoBarone, RosarioMatter, KarlBarozzi, SerenaMatters, Gail L.Barraud, PierreMatthews, DouglasBarreca, DavideMattia, LaffranchiBarresi, ValeriaMattner, JochenBarret, Jean-MarcMatulewicz, PawelBarreto, GoncaloMatulić, MajaBarro, LassinaMaturi, MirkoBarros, Marcelo PaesMaturi, VarunBarrow, TimothyMaurer, BarbaraBarshtein, GregoryMaurizio, BattinoBartesaghi, RenataMauro, AnnunziataBarthakur, SharmisthaMauro, NicolòBartlett, MaggieMaurya, ShailendraBartnik, EwaMavragani, Clio P.Bartoszewska, SylwiaMavrikou, SofiaBarwe, Sonali P.Mavrogianni, Vasia S.Barykin, EvgenyMay, Linda E.Basak, TrayambakMaya-Miles, DouglasBasar, MuratMaycotte, PaolaBaşçı, SemihMayerhofer, ArturBasharat, ZarrinMayes, MaureenBashir, SaqibMaylem, Excel RioBashir, ZafarMazaira, GiselaBasiak, MarcinMazan-Mamczarz, KrystynaBasile, GiorgioMazur-Bialy, AgnieszkaBasile, UmbertoMazurek, HenrykBasini, GiuseppinaMazzoccoli, Gianluigi UbaldoBasioura, AthinaMazzoccoli, LucianoBassi, Anna MariaMazzone, PellegrinoBasso, EmyMazzuoccolo, Luis DanielBasson, Marc D.McCall, JenniferBastian, LorenzMcClements, LanaBastihalli, Tukaramra DiwakarMcComb, ScottBasu, ParamitaMccorkle, Joseph RobertBasu, SoumyaMcDonald, PatrickBasu, SupratimMcdonnell, ThomasBatenburg, Nicole L.McDougal, David HarryBateup, HelenMcDowell, Colleen M.Baticic, LaraMcFadyen, JamesBatish, MonaMcGrady, NolanBatsalova, TsvetelinaMcgregor, NeilBattaglia, SimoneMcgrew, MikeBattista, MarcoMcIlwrath, Sabrina L.Battistelli, CeciliaMcKay, RonBattistuzzi, GianantonioMcKay, TinaBaum, OliverMcKechanie, Andrew G.Bavananthasivam, JegarubeeMcKenzie, DebbieBaxter, LauraMcKeown, LynnBayer, WibkeMcKim, Kim S.Bayin, N. SumruMcloon, Linda K.Baykov, AlexandeMcLoughlin, Hayley S.Bayona-Bafaluy, María PilarMcMahon, Timothy JosephBayrak, Ömer FarukMcMillan, DavidBazan-Socha, StanislawaMcMillin, MatthewBazil, JasonMcMillin, Shawna L.Bazopoulou, DaphneMeans, Robert T.Bazwinsky-Wutschke, IvonneMeda, Venkata SaiBazzan, EricaMedalia, OhadBear, ChristineMedas, DanielaBeard, MatthewMedcalf, Robert L.Beaujean, NathalieMedeiros, Isac Almeida deBeberok, ArturMederos, Crespo SaraBecciolini, AndreaMedhat, DaliaBechmann, NicoleMedina, Daniel A.Beck, SarahMedvedev, Roman Y.Becker, EstherMeena, JairamBeckmann, AnjaMeerson, AriBednarsch, JanMege, Jean-LouisBednarski, Tomasz K.Mehanna, EmanBeer-Hammer, SandraMéhes, GáborBeghé, BiancaMehrotra, ShikharBegonja, Antonija JurakMeier, Kathryn E.Behar, Oded Z.Meijer, LisetheBehr, BjörnMeiners, SilkeBehrens, María IsabelMeir, MichaelBehringer, ErikMeira, LisianeBei, WeijianMeireles, ManuelaBeisswenger, ChristophMeistermann, DimitriBejinariu, AlexandruMejía, MayraBeksac, MeralMejías, RebecaBelaidi, Abdel AliMeka, Durga PraveenBelayew, AlexandraMekala, NaveenBelderbos, Mirjam E.Mekhemar, MohamedBelfiore, AntoninoMelcangi, Roberto CosimoBell, Aaron W.Melchior, ChloeBellan, Cristiana.Meléndez-Hevia, EnriqueBellei, BarbaraMeli, AlbanoBellelli, RobertoMelica, Maria ElenaBellien, JeremyMello, TommasoBellini, Maria JoseMelloul, EmmanuelBellocchio, LuigiMelo-Carrillo, AgustinBelluti, SilviaMemè, LuciaBelluzzi, ElisaMemon, BushraBeloborodova, Natalia V.Men, HongshengBelosludtsev, KonstantinMen’shikov, M. YuBeltrame, Flávia L.Menale, CiroBeltrán-Partida, ErnestoMenck, KerstinBelyaeva, Olga V.Mendes, FabioBelyakov, MikhailMendes-Jorge, LuísaBelyavsky, Alexander V.Méndez-Álvarez, EstefaníaBem, Reinout A.Menegon, AndreaBenassarou, MouradMeneses, Maria JoaoBénazéraf, BertrandMeneses, PatricioBencivenga, DeboraMenezes-Rodrigues, Francisco SandroBéné, Marie C.Meng, FanjuBenedetti, AntonioMeng, FanwangBenelli, RobertoMeng, JialinBenfaremo, DevisMeng, LinyanBengel, PhilippMeng, QingshuBeninati, SimoneMeng, ZhipengBenkahla, MehdiMenke, AndreBenkhoucha, MahdiaMenke, Aswin L.Benmerah, AlexandreMenna, ElisabettaBennardo, LuigiMenon, Archita VenugopalBennett, DavidMensah, Afua AdjeiwaaBennett, James P.Meo, Ermelinda DeBennett, JohnMeraviglia, SerenaBen-Nissan, GiliMercadante, SebastianoBenvenuti, FedericaMercati, FrancescaBenzaquen, JonathanMerched, Aksam J.Bepari, Asim KumarMerimi, MakramBerdasco, ClaraMérmoud, AndréBerezhnaya, ElenaMerrick, B. AlexBerezin, Alexander E.Merrick, BlairBergant, MartinaMerrick, William C.Bergantini, LauraMerritt, MatthewBerge, ToneMertas, AnnaBerger, AdelineMertens, ChristinaBerger, Joseph R.Mertens, PeterBerghoff, MartinMertowska, PaulinaBergler, HelmutMertowski, SebastianBergman, LinaMesirca, PietroBergougnoux, VéroniqueMesquita, Fernanda Cristina PaccolaBergquist, MariaMessedi, MeriamBerg-Sorensen, KirstineMesselodi, DariaBernabe, García MarielaMessi, PatriziaBernad, AntonioMessina, AntoniettaBernal, Juan A.Meštrović, TomislavBernardes, Emerson S.Metwally, ElsayedBernardes, Joana P.Metzger, MartinBernardini, ChiaraMetzinger, LaurentBernardini, GiuliaMeyer, Jason S.Bernardino, Raquel L.Meyer-Almes, Franz-JosefBernardo, AntoniettaMezhuev, Yaroslav O.Bernareggi, AnnalisaMeznaric, MarijaBernasconi, CorradoMezzelani, AlessandraBernatik, OndrejMhatre, SiddhitaBernecker, ClaudiaMia, SobujBernhardt, AnneMialet-Perez, JeanneBernhardt, NadineMiao, WeiBernstein, Audrey M.Miazek, ArkadiuszBernstein, Hans-GertMicale, VincenzoBernstein, Sanford I.Miceli, VitaleBernstein, Steven LanceMichaelsen-Preusse, KristinBerrien-Elliott, Melissa M.Michalek, KatarzynaBerrino, EnricoMichalopoulos, IoannisBerrocal-Lobo, MartaMichel, Martin ChristianBerta, TemuginMicheroli, RaphaelBertacchi, MicheleMichita, Rafael TomoyaBertelli, EugenioMichlewski, GracjanBertoli, AlessandroMicioni, Di BonaventuBertolini, EdoardoMickleborough, Marla J. S.Bertolino, PhilippeMiddendorff, RalfBertomeu, Jesus M.Midorikawa, YutakaBertram, Christof AlbertMielczarek-Palacz, AleksandraBertuccio, Maria PaolaMierek-Adamska, AgnieszkaBerzins, StuartMieszkowski, JanBesse, SophieMietzsch, UlrikeBestwick, MeganMiftakhova, ReginaBetekhtin, AlexanderMigas, LukaszBette, MichaelMigita, SatoshiBeutner, GiselaMigliaccio, Anna RitaBevilaqua, Rangel ErikaMigoń, DorianBeyer, Eric C.Mihaicuta, StefanBezprozvanny, Ilya B.Mihaila, MirelaBharadwaj, AlameluMihály, JózsefBharadwaj, VimalaMihasan, MariusBhargava, VarshaMikheev, IvanBhaskar, RakeshMiki, DaisukeBhatia, DhirajMikołajczak, PrzemysławBhatia, DivyaMikula, MarioBhatnagar, NoopurMikulska-Ruminska, KarolinaBhattacharya, AbhisekMilanovic, DesankaBhattacharya, PallabMilardi, DaniloBhattacharya, SantanuMilenkovic, Vladimir M.Bhattacharya, SomanonMiličić, Tanja J.Bhawal, Ujjal KumarMiljković, Miloš D.Bhawe, KaumudiMillan, JaimeBhoopathi, BhoopathiMillar, J. CameronBhullar, Khushwant SinghMiller, AdamBi, LiangkuanMiller, John H.Biagini, GiuseppeMiller, KarlBiałek, SławomirMillet, ArnaudBialkowska, KatarzynaMills, KenBian, ShanMillward, StevenBian, YueminMin, KisukBianchetti, GiadaMin, ZhangBianchetto-Aguilera, FranciscoMinafra, LuigiBianchi, NicolettaMinas, GraçaBianco, Piero R.Minea, RaduBianucci, GiovanniMinelli, RosalbaBichet, Daniel G.Minerick, AdrienneBicknell, BrianMinetti, GiampaoloBiersack, BernhardMinic, ZoranBifulco, MaurizioMiniero, Daniela ValeriaBigi, AlessandraMinnes, RefaelBijlsma, Maarten F.Minoli, LuciaBikov, AndrasMinser, James S.Bild, VeronicaMinshall, RichardBilgory, AsafMinutolo, AntonellaBilic-Ćurčić, InesMiotto, BenoîtBilkei-Gorzo, AndrasMirabelli, MariaBilski, JanMirakaj, ValbonaBinder, RobertMiralrio, Pineda AlanBindu, Dhanesh SivadasanMiranda, JezidBinetruy, BernardMiranda, Joana P.Biocca, SilviaMirko, ManchiaBiondi, GiuseppinaMirmira, Raghavendra G.Birge, Raymond B.Miró Roig, JordiBirn, HenrikMiroshnikova, Valentina V.Bironaité, DaivaMirshahi, RezaBirukov, Konstantin G.Mirzaei, RasoulBisaccia, FaustinoMiserocchi, GiacomoBisbal, MarianoMisery, LaurentBisio, AngelaMishra, AbhinavaBiskup, EdytaMishra, Ajay KumarBisserier, MalikMishra, AvinashBiswas, BanhiMishra, DeepshikhaBiswas, DebolinaMishra, Gyan PrakashBiswas, JyotirmayMishra, Hemant KumarBixby, John L.Mishra, KrishnaveniBjörn, Lars-OlofMishra, RiteshBlahova, JanaMishra, SushilBlaine, JudithMiska, Jason MichaelBlalock, WilliamMisra, PrashantBlanco-Colio, LuisMiszalski, ZbigniewBlanco-Suarez, ElenaMiszczyk, JustynaBlank, UlrichMitani, ShoheiBlanquart, ChristopheMitchell, Cassie S.Blanquer, AndreuMitchell, Patricia L.Blas-García, AnaMiteva, KapkaBlattner, ChristineMitic, TijanaBlicharski, TomaszMitola, StefaniaBlitshteyn, SvetlanaMitov, MihailBloise, NoraMitry, RagaiBloomfield, MathewMitsikostas, DimosBloomfield, SusanMiyagawa, TomoakiBoakari, Yatta LinharesMiyashiro, DenisBobba, Kondapa NaiduMiyauchi, SeijiBobe, RegisMiyazaki, TakuroBobrovskaya, LarisaMiyoshi, KeikoBoccaccini, AlessandraMizokami, AkikoBodas, ManishMizsei, RékaBodman-Smith, MarkMizushima, NoboruBoehme, Karen A.Mlinarić, SelmaBoeuf, HélèneMlitz, VeronikaBoffano, PaoloMlynarz, PiotrBogdanov, Alexey M.Mnasri, SamiBogolyubov, DmitriyMnich, KatarzynaBoguenet, MagalieMnif, BasamaBogusławska, JoannaMo, DelinBohmwald, KarenMocchetti, ItaloBoitani, CarlaMochizuki-Kashio, MakikoBojarska-Junak, AgnieszkaMocsár, GáborBojić, TijanaModel, MichaelBojko, Evgeny R.Modica, RobertaBollini, SvevaMoens, UgoBommireddy, RamireddyMoghaddam, Banaem LidaBonacchi, MassimoMohamed, Maged E.Bonaccorsi Di Patti, Maria CarmelaMohamed, TamerBonanad, SantiagoMohammad, MobashirBoncompain, GaelleMohammad, SameerBonetto, ValentinaMohammadabadi, M. R.Bonfanti, LucaMohanta, SarajoBonfert, Michaela VeronikaMohanty, BijayalaxmiBonfill, MercedesMohanty, SujataBongarzone, ItaliaMohany, MohamedBönig, Halvard B.Mohapatra, BhopalBonin, SerenaMohar, BoazBonini, Sara AnnaMohd, Khairi MdBonner-Weir, SusanMohibbullah, Md.Bono, María RosaMohibi, ShakurBoo, Yong ChoolMohiuddin, OmairBoon, SiawMöhn, NoraBoonen, MarielleMohr, SusanneBooth, BrianMohr, ThomasBoradia, VishantMohtar, M. AimanBoratto, PaoloMoi, Sankar ChBordet, Jean-ClaudeMoiseev, Alexander A.Borek, SławomirMojarad, Ehsan NazemalhosseiniBorg, JosephMojsilović, Slavko B.Borges, Andres A.Mokhtari, TahminehBorggrefe, MartinMoldovan, FlorinaBorghetti, PaoloMolins, BlancaBorghi, SergioMollaoglu, GurkanBorgia, Jeffrey A.Molnár, KingaBorhani-Haghighi, MaryamMomčilović, Stefan D.Boriek, Aladin M.Mondal, DibakarBornman, RianaMonje-Casas, FernandoBorocci, StefanoMonk, PeterBorovikov, YuriiMonory, KrisztinaBorowski, Lukasz S.Monson, EbonyBorrell-Pages, MariaMontagnese, FedericaBorro, MarinaMontalbán, Itziar A.Borroni, DavideMontalbano, GiuseppeBortner, Carl D.Montaño, Ramírez LuisBortolin, Raul HernandesMontarolo, FrancescaBortolotto, TissianaMontecucco, FabrizioBortoluzzi, AlessandraMonteiro, Luís SilvaBosáková, MichaelaMonteith, Teshamae S.Boscardin, Silvia BeatrizMontenarh, MathiasBoschetti, ElisaMontenegro, LuisBose, BipashaMontenegro, María F.Boshra, SylviaMontgomery, McKaleBos-Mikich, AdrianaMontgomery, Samuel T.Bosnakovski, DarkoMooers, BlaineBosserhoff, Anja KatrinMooli, Raja GopalBossowska, AgnieszkaMoon, II SooBot, IlzeMoon, In SeokBott, AlexMoon, JisookBottai, DanieleMoore, CarleneBottari, FabioMoore, Ronald B.Böttcher, MartinMoore-Carrasco, RodrigoBotuyan, Maria VictoriaMora, Delio JoséBoudaka, Ammar AbdullkaderMorabito, AntoninoBouhassira, EricMorabito, RossanaBouissane, LatifaMorad, MartinBouitbir, JamalMoraes, João AlfredoBoulant, SteeveMorales, ManuelBouley, RichardMorales-González, José AntonioBourdin, ArnaudMorales-Lázaro, Sara LuzBourgeois, PatriceMorani , FedericaBourgoin, Sylvain G.Morani, FedericaBouschet, TristanMora-Rodríguez, RodrigoBouskila, JosephMoratalla, RosarioBoutillier, Anne-LaurenceMorava, EvaBowen, AnthonyMorbidelli, LuciaBoyce, John D.Morciano, GiampaoloBoyman, LironMore, ShyamBozzini, SaraMore, Swati S.Brabek, JanMoreau, ViolaineBracco, EnricoMoreira, VanessaBradbury, PetaMoreira-Filho, Carlos AlbertoBradshaw, NicholasMoreno, HectorBradshaw, PatrickMoreno, HeitorBrady, Graham F.Moreno, Ricardo D.Brady, KristenMoreno-Loshuertos, RaquelBraga, Susana SantosMoreno-Navarrete, José MaríaBraicu, CorneliaMoretti, FabiolaBrancaccio, AndreaMoretti, SoniaBrancaccio, MaraMorgan, MichaelBranco, CristinaMorgan, NoelBrandalise, FedericoMori, GiorgioBrandenburg, Lars OveMorigny, PaulineBrandi, Maria LuisaMorimoto, TatsuyaBrandt, SabineMorin, NathalieBras, EduardoMorino, KatsutaroBrasoveanu, Lorelei IrinaMorio, YoshiteruBratseth, VibekeMorisco, CarmineBratus, AlexanderMorisco, FilomenaBrault, JeffreyMoriscot, AnselmoBraun, DanielaMorishita, HideakiBraun, FrankMorita, MasanobuBraun, RalfMoriyama, MarikoBrautigan, David L.Morley, Steven DouglasBravo, Santos RafaelMorocho, VladimirBravo, Susana B.Möröy, TarikBrech, AndreasMorrione, AndreaBréchard, SabrinaMortensen, Luke J.Breiderhoff, TilmanMortier, ErwanBreitbart, HaimMorucci, GabrieleBrelot, AnneMoruno Manchon, Jose FelixBrenmoehl, JuliaMoschini, RobertaBrenner, TalmaMoser, MarkusBreunig, JoshuaMosieniak, GrazynaBreuzard, GillesMosińska, PaulaBrezovakova, VeronikaMoskvin, SergeyBrignole, ChiaraMossa, Abubakr H.Brisca, GiacomoMotamed, CyrusBrites, DoraMotazedian, AliBritto-Borges, ThiagoMotevaseli, ElaheBrizuela, LeyreMotiani, RajenderBrocca, StefaniaMotohashi, NorioBrockbank, Kelvin G. M.Motta, ChiaraBrocke, StefanMottarlini, FrancescaBrodsky, SergeyMotti, Maria LetiziaBrody, MatthewMottola, GiovannaBroggi, GiuseppeMotýl, JiříBrook, John DavidMougeot, Jean-LucBrooks, BrianMoura, Fabiana AndréaBrooks, DavidMoura, Marcelo TigreBrooks, Tracy A.Mousley, CarlBros, MatthiasMoussawi, KhaledBross, PeterMoya, Iván M.Broude, Eugenia V.Moyse, EmmanuelBrown, Christopher JohnMozdziak, PaulBrown, JayMozzetta, ChiaraBrown, LindsayMrabet, SalouaBrowne, CarolineMridha, M. FirozBrozel, VolkerMrówka, PiotrBruce, A. BunnellMsaouel, PavlosBrugnoli, FedericaMu, PeiqiangBrundel, BiancaMu, QingxinBrunetta, HenverMu, XinBrunetti, DarioMubarak, Bashayer AlBrunetti, GiacominaMuccee, FatimaBrun-Heath, IsabelleMucientes, ArkaitzBruni, PaolaMueller, MatteoBruno, FrancescoMuhovski, YordanBruno, MeloniMujtaba, HasanBrusco, AlfredoMukai, TakahitoBryson-Richardson, RobertMukherjee, ChandramaBrzóska, EdytaMukherjee, ManaBrzóska, TomaszMukherjee, NabanitaBrzostek, JoannaMukherjee, SayaliBrzuzan, PawełMukhopadhayay, Chander ShekharBucci, CeciliaMukhopadhyay, SubhadipBucci, MarcoMukund, KavithaBucciantini, MonicaMukwaya, AnthonyBuch, ShilpaMulet, Jose M.Büch, ThomasMüller, Andreas MarcBuchaim, Daniela VieiraMüller, Hans-ArnoBucher, SimonMuller, KarelBuchet, ReneMuller, Yunhua LiBüchler, ChristaMüller-Taubenberger, AnnetteBüchler, TomášMullick, MadhubantiBuchwalow, Igor BorisowitschMullins, Robert F.Buck, Michael D.Munday, MichaelBucki, RobertMunell, FrancinaBucolo, ClaudioMunetsuna, EijiBudani, Maria CristinaMunjas, JelenaBudini, MauricioMunker, ReinholdBuendía, Roldán IvetteMuñoa, Hoyos IraiaBueno, MilagrosMuñoz, AnaBuescher, MaritaMuñoz, Juan PabloBuettner, ReinhardMunson, GeorgeBuffone, CaterinaMünsterberg, AndreaBugert, PeterMuraki, KatsuhikoBujko, MateuszMural, Ravi V.Bujold, EmmanuelMuraleva, Natalia A.Bukhari, IhtishamMurali-Krishna, KajaBulfone-Paus, SilviaMuranov, KonstantinBulut, ErdoganMurata, SoichiroBunick, ChristopherMurata, TakayukiBuono, Nicoletta DelMuratani, MasafumiBuono, PasqualinaMuratore, EdoardoBuonvicino, DanielaMuratori, LuigiBurada, FlorinMurciano-Calles, JavierBuranasudja, VisarutMurdaca, GiuseppeBurbulla, Lena F.Mürdter, ThomasBurdach, StefanMuresu, NarcisaBurdzinska, AnnaMurooka, ThomasBurek, MalgorzataMurphy, RonanBurelle, YanMurphy, William J.Burgess, David R.Murray, Nicole R.Burghardt, KyleMurray, Thomas ScotBurgos-Robles, AnthonyMurru, ElisabettaBurgute, BhagyashriMurthi, PadmaBurico, MichelaMurthy, DivyaBurks, JaredMurugan, KumarBurlacu, AlexandruMuruzabal, FranciscoBurman, AnkitaMusani, VesnaBurnap, RobMusatov, AndreyBurrell Bustos, María ÁngelaMusazzi, LauraBurrello, JacopoMushtaq, MuntazirBurster, TimoMushtaque, MdBurton, AdamMusiał, KingaBuschmann, JohannaMussagy, Cassamo U.Bushart, David D.Mussbacher, MarionBusquets, AntonioMustelin, TomasButcher, Lee RyanMusti, Anna MariaButini, StefaniaMusto, Alberto E.Butler-Browne, Gillian SandraMusumeci, FrancescaButt, ElkeMuszyński, SiemowitButtigliero, ConsueloMuthu, Madaswamy S.Byrne, NikoleMuthukrishnan, Sree DeepthiBystrykh, LeonidMuthuraman, ArunachalamByun, SanguineMutis, TunaByun, Woong SubMutoh, TatsuroCaballero-Villarraso, JavierMutolo, DonatellaCaballo, Mar CarriónMuxel, Sandra MarciaCabañas, CarlosMűzes, GyörgyiCabral Portugal, CamilaMuzza, MarinaCabukusta, BirolMwangi, PeterCacciola, Emma C.Myakala, KomuraiahCacciola, FrancescoMyers, Michael J.Cacciola, RossellaMylonas, Konstantinos S.Caccuri, FrancescaMylonis, IliasCachón-Gonzalez, María BegoñaMyöhänen, Timo T.Cacopardo, LudovicaMysliveček, JaromírCadamuro, MassimilianoMzoughi, SlimCadoret, VéroniqueNabissi, MassimoCaetano, GuilhermeNadalin, SilvioCaffo, MariaNadir, Zaman NadirCaggiati, AlbertoNaeem, Muhammad KashifCagnin, StefanoNagafuchi, SeihoCahuzac, MaximeNagahara, YukitoshiCai, GiampieroNagai, AtsushiCai, JasonNagai, Luis Augusto EijyCai, JingwenNagalakshmi, UgrappaCai, JiyangNagamura-Inoue, TokikoCai, LuNagano, ChinaCai, QingqingNagaoka, KentaroCai, WeikangNagappan, ArulkumarCaiazzo, MassimilianoNagaraj, SiranjeeviCaillet-Saguy, CéliaNagata, AliceCaiola, ElisaNagatomo, YujiCairns, DanaNägerl, ValentinCairrao, ElisaNagler, ArnonCalabrese, Francesco MariaNagre, NagarajaCalaf, GloriaNaguib, Deyala MohamedCalafiore, DarioNagy, MagdolnaCalamita, GiuseppeNagy, TiborCalbo, SebastienNagy-Grócz, GáborCalcabrini, AnnaricaNah, JihoonCaldwell, R. WilliamNaif, Alhussien MohannedCaldwell, Ruth B.Nair, AnupCali, JamesNair, JayakumarCalì, TitoNair, SharmilaCallander, Natalie ScottNajdekr, LukášCallicott, Kenneth A.Nakagawa, TakuroCalucho, MaiteNakahara, YukikoCalvert, JohnNakai, KeiCalvisi, Diego F.Nakamura, KazuakiCalzada, FernandoNakamura, KazufumiCalzetti, GiacomoNakamura, MasanaoCalzolari, FilippoNakamura, NobuhiroCamargo, MarianaNakamura, TomokiCamino, José DanielNakamura, YoshikazuCammas, FlorenceNakamura, YoshiyukiCamões, Sérgio P.Nakatani, SachieCampa, Carlo CosimoNakaya, NaokiCampadelli-Fiume, GabriellaNakazawa, MitsuruCampagna, RobertoNakazawa, TsutomuCampaner, StefanoNaldini, AntonellaCampilho, AnaNale, JanetCampolo, JonicaNalepa, IrenaCampos, Pereira TiagoNaletova, IrinaCampusano, Jorge M.Nallasamy, PalanisamyCanaider, SilviaNam, Mi-HyunCancela, Josè ManuelNam, Min-HoCancemi, PatriziaNambeesan, Savithri U.Canchi, SaranyaNamgaladze, DmitryCancino, JorgeNampoothiri, MadhavanCancio, IbonNanda, SatyabrataCanepari, MonicaNanni, LorisCanetta, ElisabettaNapoli, SaraCannon, JasonNappini, SilviaCanosa, StefanoNarayanan, RameshCansian, Rogério LuisNarayanan, S. PriyaCantacorps, LidiaNarboux-neme, NicolasCantarella, GiuseppinaNardelli, SilviaCanterini, SoniaNaroui, Rad MohammadCantin, André M.Narra, SudhakarCantó, CarlesNaruse, KatsuhikoCantoni, ClaudiaNascimento da Silva, Luís CláudioCantore, StefaniaNascimento, KyriaCao, DengchaoNaser-Moghadasi, AbdorrezaCao, DongdongNash, KevinCao, HongNashar, TouficCao, KanNashine, SonaliCao, QilinNaskar, ShovanCao, XuanyeNasri, ZahraCaparrós, EstherNassa, GiovanniCaparros-Martin, JoseNasseri, Mohammad AliCapasso, DomenicaNasso, GiuseppeCapellari, OrnellaNassoy, PierreCapelli, DeboraNataf, SergeCapellino, SilviaNatale, GianfrancoCapitanio, NazzarenoNatalia, V. KlementievaCaplan, AllanNatalicchio, AnnalisaCaponio, Vito CarloNatarelli, LuciaCapozzi, AntonellaNatella, RaffaeleCappelli, ClaudioNath, Lekshmi R.Caraci, FilippoNatsuka, ShunjiCarafa, VincenzoNatsumeda, ManabuCaraglia, MicheleNava, SaraCarbone, CarmineNava, Victor E.Carbonelli, CristianoNavarrete, FranciscoCarbonero, FranckNavarro, CésarCarboni, LuciaNavarro, CristinaCarcangiu, VincenzoNavarro, EstanislaoCardenas, Ana M.Navarro, GemmaCardnell, RobertNavarro, LuzCardol, PierreNavarro-Langa, Juan AntonioCaretti, GiuseppinaNavarro-Tapia, ElisabetCargnoni, AnnaNavas, AlfonsoCariboni, AnnaNava-Villalba, MarioCaric, DavorNavolan, Dan-BogdanCaricato, AnselmoNavone, StefaniaCarini, RitaNawarawong, Natalie N.Carl, MatthiasNawaz, MuhammadCarlberg, CarstenNayak, Nihar RanjanCarleo, AlfonsoNayeem, Mohammed A.Carlini, CéliaNaylor, AmyCarlos, Ana RitaNazir, FarozaCarlsson, HenrikNeagu, AdrianCarmina, EnricoNebigil, CananCarmona-Mora, PaulinaNechaev, SergeiCarneiro, Sueli CoelhoNedelcu, AuroraCarnero, AmancioNeef, JakobCaron, De FromentelNees, MatthiasCarotenuto, PietroNeesse, AlbrechtCarotenuto, RosaNeeta, BhagatCarotti, SimoneNeganova, IrinaCarpaneto, ArmandoNeganova, MargaritaCarpenter, JohnNegm, AmrCarpentieri, AndreaNegoro, HiromitsuCarraro, MichelaNegri, FrancescaCarrascal, LiviaNegroiu, GabrielaCarriba, PaulinaNejati, RezaCarriero, MariaNekkar Rao, Praveen P.Carrillo, WilmanNelson, Chase W.Carrillo-Salinas, FranciscoNelson, DavidCarrión, MarNemeș, Roxana MariaCarro, Maria StellaNemeth, NorbertCarrotta, RitaNeri, MargheritaCarswell, HilaryNeri, TommasoCartarozzi, Luciana PolittiNesmelov, YuriCarter, WayneNeubauer, HansCaruana, IgnazioNeubauer, KatarzynaCarullo, GabrieleNeupane, Yub RajCaruso-Neves, CelsoNeves, DelmindaCarvalho, Andreia N.Neves, Renata HeislerCarvalho, CristinaNeviani, PaoloCarvalho, EdmundNewton, JasonCarvalho, Hernandes F.Ng, Beng KwangCarvalho, JoanaNg, ChinCarvalho, Lina A. S.Ngomba, RichardCarver, WayneNguyen, Huu PhucCasalou, CristinaNguyen, Ly Thi HuongCasarini, LivioNguyen, Thi-HuongCascardi, ElianoNí, Dhubhghaill SorchaCascella, RaffaellaNi, ZhiyongCasciaro, FrancescaNiazian, MohsenCasciati, AriannaNibbio, GabrieleCasella, GiovanniNicoara, Simona DeliaCasey, JenniferNicola, Juan PabloCasimiro, SandraNicolau, StefanCasiraghi, FedericaNicoletti, FerdinandoCaspari, ThomasNicolini, GiuseppinaCasselbrant, AnnaNicolini, Laura AmbraCastagna, AnnalisaNicolosi, DariaCastanha, Priscila MayrelleNicoś, MarcinCastaño-Zapata, JairoNicosia, AldoCastellani, StefanoNiculae, MihaelaCastellano-Pozo, MaikelNiculescu, Loredan StefanCastelucci, PatriciaNie, HaoCastets, MarieNie, ShuyiCastiello, LucianoNiechi, IgnacioCastiglione, RobertoNiedźwiedzka-Rystwej, PaulinaCastiglioni, BiancaNiello, MarcoCastoldi, MircoNielsen, MortenCastro, FláviaNiemann, HeinerCatalano, TeresaNighot, PrashantCataldi, AmeliaNiihara, YutakaCatar, RusanNiikawa, HiromichiCatargi, BogdanNikalje, GaneshCaterino, MariannaNikić-Spiegel, IvanaCattane, NadiaNikitovic, DraganaCattaneo, Maria GraziaNikolakopoulou, PolyxeniCatterall, William A.Nikolettos, NikosCaturano, AlfredoNikolic, LjubisaCauchie, PhilippeNikulin, AlexeyCaudle, William MichaelNiland, StephanCauffiez, ChristelleNilsson, PeterCavaliere, FabioNilsson-Janke, MariaCavarsan, Clarissa FantinNimura, KeisukeCayre, MyriamNina, BrutchCazzaniga, StefanoNinkina, NataliaCeccarelli, GabrieleNinkovic, JovicaCeccarelli, IlariaNirala, Niraj K.Cecconi, SandraNishida, KeiCecília, R. C. CaladoNishida, MotohiroCeckova, MartinaNishiguchi, MasamichiCelada, AntonioNishiguchi, ShuheiCelebi, Sözener ZeynepNishijima, KazutoshiCelsi, FulvioNishikimi, AkihikoCenariu, Mihai CosminNishimura, Robert N.Cenci, GiovanniNishio, MizuhoCeolotto, GiulioNishitani, HideoCerda, OscarNishiyama, KazuhiroČerný, MartinNiskanen, Einari A.Cero, CherylNittby, HenriettaCerón, Madrigal JuliánNiu, Chi ChienCerović, RadosavNizyaeva, Natalia ViktorovnaCerqueira, FatimaNobuhisa, IkuoCerrato, ValentinaNoelle, DwyerCerrone, MargheritaNogales, AitorCervellati, FrancoNoguchi, Constance TomCestmir, AltanerNoguera, Nelida InesCestra, GianlucaNoh, Ka-wonCesur, MustafaNohno, TsutomuÇetin, NihalNollert, Matthias U.Cetinel, SibelNonnemacher, Michael R.Cetrulo, Curtis L.Nono, Nankam PamelaCha, Sun JooNonoguchi, HiroshiChaari, AliNoothalapati, HemanthChabert, ClovisNordio, MaurizioChabot, BenoitNoreldin, AhmedChacko, Jenu V.Noren, Hooten NicoleChacón, Iván De-La-CruzNorman, Jennifer E.Chagraoui, AbdeslamNoronha, AshishChai, JianyuanNosov, Alexander V.Chai, LiNosrati, HamedChai, Tsun-ThaiNotarangelo, MichelaChai, ZhiNotartomaso, SerenaChaitanya, Ganta VijayxNothnick, Warren B.Chakrabarti, JayatiNout-Lomas, Yvette S.Chakraborty, KausikNovak, GregorChakraborty, SayanNovák, JanChakravarti, ShuktiNovakovic, IvanaChami, MouniaNowak, DanielChamuleau, Robert A. F. M.Nowicka, DanutaChan, David WaiNowicka, GrażynaChan, GordonNowsheen, SomairaChan, OwenNtafou, DimitraChand, Jha UdayNucci, MarianaChandler, Ronald L.Núñez González, CristinaChandra, MintuNúñez, ÁngelChandra, SharatNunzio Antonio, CacciolaChandra, SunandiniNurhayati, Retno WahyuChandrasekar, IndraNurtdinov, RamilChandrasekaran, VidyaNushtaeva, AnnaChang, Chia-YuanNuzzi, VincenzoChang, Ching WenNuzzo, AndreaChang, Ching-JinNylandsted, JesperChang, Chuang-RungNyström, AlexanderChang, Gwo-JyhO’Connell, Kevin F.Chang, Hsueh-WeiO’Connor, J. PatrickChang, HuibinO’Dell, William BradChang, Hui-huaO’Donoghue, PatrickChang, Jeong HoO’Flanagan, CiaraChang, Julia Yu-FongO’Malley, DervlaChang, JunleiO’Malley, KarenChang, Ke-VinO’Morain, Colm A.Chang, Kuo-HsuanO’Neal, WandaChang, LeiO’Rourke, ColmChang, LiO’Sullivan, Kim M.Chang, LinObara-Michlewska, MartaChang, Long-SenObuchowicz, EwaChang, MeichiOchi, ShinichiroChang, Wei-TingOczkowicz, MariaChang, Wen-WeiOda, ShojiChang, Yuan-YenOda, ToshiyukiChang, Yu-ChanOda, TsukasaChanoine, ChristopheOdeh, DyanaChao, Cho-MingOdenthal, MargareteChao, Hsu-WenOeing, Christian U.Chao, Louis KuopingOffidani, MassimoCharan, ManishOfman, GastonCharbe, NitinOgawa, HidehikoChard, Louisa S.Ogihara, JunCharif, MajidaOgiwara, HideakiCharlier, ThierryOgiya, DaisukeChartrel, NicolasOgmundsdottir, Helga M.Chassain, CarineOgoyama, ManabuChatham, John C.Oh, Seh-HoonChatla, KamalakarOh, SekyungChattopadhyay, NaibedyaOhana, MickaëlChattopadhyay, SaurabhOhara, ShutaChatziantoniou, ChristosOhba, SeigoChauhan, AshwiniOhishi, TomokazuChaulin, Aleksey MichailovichOhkawara, BiseiChaurand, PierreOhlendieck, KayChauss, DanielOhnishi, ShihoChaverri, José PedrazaOhnishi, TakanoriChavez, FranciscoOhnmacht, CasparCheang, Anna Wai SanOhno, JunCheillan, DavidOhori, FumitoshiChemin, IsabelleÖhrfelt, AnnikaChen, Chang-LiangOhtsuki, SumioChen, ChaoOhya, SusumuChen, ChenOjeda-May, PedroChen, ChiderOkada, HirokiChen, ChongOkamoto, MasanoriChen, Chuan-LinOkamoto, RobertaChen, ChunOkamoto, ShigefumiChen, Chung-HwanOkamoto, YosukeChen, Chung-MingOkazaki, YasumasaChen, Chun-LiangOkovityi, SergeyChen, DongOkumura, KoChen, Edward S.Okumura, NobuakiChen, Fang-MingOkumura, RyuChen, FeiOla, RoxanaChen, George GongOláh, JuditChen, Guang-ChaoOlaru, Octavian TudorelChen, GuangjieOlaya, Abril AlfonsoChen, GuobingOlczyk, KrystynaChen, GuoxunOlejniczak, ScottChen, HanyingOlejnik, JudithChen, HaoyuOlenina, Ludmila V.Chen, Hsin-ChienOlga, PershinaChen, HuOlgasi, CristinaChen, HuiOliva, JoanChen, Hui-ChenOlivas, MiguelChen, Hung-JenOliveira, Carla C.Chen, JichunOliveira, Maria CristinaChen, JingruiOliveira, Paula A.Chen, KetingOliveira, Sonia M.Chen, KevinOliver, BrianChen, Kuang-ChiOliveri, ValentinaChen, Kuan-HsuanOliveros, Juan C.Chen, KunqiOliviero, FrancescaChen, LiangOlivos, AnellChen, Miao-HsuehOllauri-Ibáñez, ClaudiaChen, MinOllero, MarioChen, PeichaoOlotu, FisayoChen, PengOlova, NellyChen, PingOlson, Michael F.Chen, QiOlzscha, HeidiChen, QianOmar, Syed HarisChen, RuijiaoOmasa, TakeshiChen, Sean Chun ChangOmilusik, Kyla D.Chen, Sheng-HungOndrasek, GabrijelChen, ShidaOng, HweiChen, Shih-HengOnida, FrancescoChen, Shih-YinOnisto, MaurizioChen, SonghaiOnofrillo, CarmineChen, SujunOnoue, KenjiChen, Szu-TahOnukwufor, John O.Chen, TingtaoOoi, London L.Chen, WeiOosterveer, Maaike H.Chen, Wei-ChihOpländer, ChristianChen, Wei-ShengOrecchioni, MarcoChen, Wei-YuOrekhov, Philipp S.Chen, XiOrengo, James P.Chen, XiangjunOriglia, NicolaChen, XingqiOrlacchio, AntonioChen, XiqunOrlandi, CesareChen, XiuhuiOrlov, Yuriy L.Chen, XuOrlova, AnnaChen, XuesongOrlova, NadezhdaChen, Xu-QiaoOrna, Amster-ChoderChen, Y. EugeneOrnello, RaffaeleChen, YanOrren, David K.Chen, YangOrsini, CristinaChen, Yan-HuaOrsolits, BarbaraChen, YantingOrtiz, AngelícaChen, Yi-ChunOrtiz, RaulChen, Yi-FanOrtiz-Masiá, DoloresChen, YiliangOry, StéphaneChen, YingOrzi, FrancescoChen, YongqiangOsei, Emmanuel TwumasiChen, Yu-ChihOsei-Bonsu, IsaacChen, Yu-ChunOsman-Ponchet, HananChen, YuqiOsminkina, Liubov A.Chen, ZenglongOsmolovskiy, Alexander A.Chen, ZhihaoOsmulski, Pawel A.Chen, Zhi-NanOsorio-Conles, ÓscarChen, ZitaoOsowiecka, KarolinaChen, ZixinOsredkar, JoškoCheng, Chun-ChiaOstaszewski, PiotrCheng, HaoOsti, Mattia FalchettoCheng, Hung-ChiÖstlund-Farrants, Ann-KristinCheng, JizhongOstroumova, EvgeniaCheng, Juei-tangOszajca, KatarzynaCheng, Kuang-HungOta, AkinobuCheng, PengOtáhal, JakubCheng, QingsuOtero, CarolinaCheng, XinOthman, Eman M.Cheng, YingOtosu, TakuhiroCheng, ZhenOtsuru, SatoruChengyi, SunOttoboni, LindaChen-Yoshikawa, Toyofumi FengshiOtto-Buczkowska, EwaCheon, Yong-PilOttosson, DaniellaChérel, IsabelleOtulak-Kozieł, KatarzynaCherepanov, Stanislav M.Ouarné, MarieCherkaoui-Malki, MustaphaOudart, Jean-BaptisteChernyak, BorisOuled-Haddou, HakimCherukula, KondareddyOuni, MeriemChevallier, LucieOura, ShojiChevigné, AndyOuro Villasante, AlbertoChi, JingyiOvtchinnikov, IgorChi, WeiOwczarczyk-Saczonek, AgnieszkaChiabrando, Gustavo AlbertoOwen, GarethChiang, JohnOwens, Raymond J.Chiang, Yi-fenOza, GoldieChiaramello, AnneOzaki, ShujiChiavacci, Leila A.Ozawa, Shin-IchiroChiba, TomohiroOzcelik, AdemChiba, ToshimiOziębło, DominikaChieffi Baccari, GabriellaOzkaya, NevalChiellini, GraziaOzpinar, AyselChilicka, KarolinaOzretić, PetarChilton, LisaOzturk, MehmetChin, Siok-FongOzturk, MelekChin, Yu-TangPaauwe, MadelonChinen, JavierPabelick, Christina M.Chinigò, GiorgiaPace, FabioChinthalapudi, KrishnaPace, LauraChiodelli, PaolaPace, Raffaella DeChiritoiu, MariPacella, ElenaChitko-Mckown, CarolPacey, AllanChiu, IngmingPacheu-Grau, DavidChiu, Norman H.Paciello, FabiolaChiu, Yi-LinPaczesny, ŁukaszChivero, ErnestPadavannil, AbhilashChlichlia, KaterinaPadhiar, ArshadChmielewski, MichalPadrão, Ana IsabelCho, Dong-HyungPădureanu, VladCho, HyungseokPagano, AlessandraCho, JaePage, JesúsCho, JongkiPagliaro, PasqualeChoi, Mi-RanPahlavan, SaraChoi, MurimPai, Pei-YingChong, KiltoPaiva, ArturChou, Chia-HungPal China, ShyamsundarChou, Tsung-HanPal, SoumojitChoubey, MayankPalacios-García, IsmaelChoudhary, MayurPaladini, PieroChoudhury, Mohammed E.Palanski, BradChoudhury, SujataPałasz, ArturChouhan, V. S.Palazzolo, GemmaChowdhury, BiswajitPalermo, RoccoChowdhury, SayantaniPall, EmokeChristensen, KarenPallagi, PetraChristensen, Sarah LouisePallio, GiovanniChristenson, Lane K.Palma, Sircili MarceloChristian, KönigPalmieri, Erika M.Christin-Maitre, SophiePalmour, RobertaChristofides, Elena A.Palomba, StefanoChristofoletti, GustavoPalomo, ValleChristoforou, NicolasPalou, MarionaChu, ChenPalù, GiorgioChu, Cheng-YingPaludo, Giane ReginaChu, John Man TakPalumbo, SaraChu, Pei-YiPamidi, NarendraChu, ShifengPampana, SilviaChu, Xiang-PingPan, ChunChua, Kien HuiPan, Ling-yaChua, Su-KiatPan, Ming-KaiChuang, Hui-ChingPan, QingjunChuang, Jen-PinPan, Shin-ChenChuang, Jian-YingPan, Tai-LongChudek, JerzyPan, ZhichengChueh, Pin JuPanaitescu, CarmenChuffa, Luiz Gustavo de AlmeidaPanaro, Maria AntoniettaChugh, Rishi ManPanayotova-Dimitrova, DianaChumnanpuen, PramotePancaldi, VeraChun, Kyung-SooPancsa, RitaChung, ChaeukPanda, Siva PrasadChung, Doug D.Pandey, AshutoshChung, Hwan-SuckPandey, Kapil D.Chung, Jay H.Pandey, ManojChung, KennyPandey, NootanChung, Patrick Ho YuPandey, PraveenChung, Yoon-SeokPandey, RajeshChunlin, ShaoPandey, Ram VinayChutipongtanate, SomchaiPandey, VijayChymkowitch, PierrePandita, Tej K.Ciarambino, TizianaPandolfo, Savio DomenicoCicatiello, Annunziata GaetanaPandruvada, SubramanyaCicco, SebastianoPanic, MarkoCiccone, MarcoPanickar, KiranCiccone, ValerioPanicucci, ChiaraCichero, ElenaPanina, Larissa V.Cichorek, MirosławaPankonien, InesCicuéndez, MónicaPanneerselvam, JananiCid, EmiliPanopoulos, StylianosCid, Víctor J.Pansarasa, OriettaCidre-Aranaz, FlorenciaPanse, Jens P.Ciemerych-Litwinienk, AnnaPant, DeveshCieślińska, AnnaPant, KishorCifone, Maria GraziaPantaleão, Lucas CarminattiCiftci, HalilibrahimPanteris, EmmanuelCiliberti, Maria GiovannaPantsar, TatuÇinar, ÖzgürPanwar, AmitCingaram, PradeepPanzetta, ValeriaCingoz, OyaPaolini, MorenoCiobică, AlinPaolini, RossellaCioffi, Marcelo de BelloPaolo, DarioCione, ErikaPapaccio, GianpaoloCisneros, BulmaroPapachristos, ApostolosCisneros-Mejorado, AbrahamPapadimitriou, EvangeliaCiteroni, Maria RitaPapadopoulos, Dimitris P.Clanchy, FelixPapait, AndreaClaude-Taupin, AurorePapakonstantinou, Panteleimon E.Clayton, AndrewPapandreou, IoannaClemens, DahnPapaneophytou, ChristosClement, AlbrechtPapis, KrzysztofClement, CristinaPaplińska-Goryca, MagdalenaClementi, M. ElisabettaPapp, BélaClifford, RoycePappritz, KathleenClift, Cassandra L.Paquet, DominikCloninger, MaryPaquin-Proulx, DominicCmarko, DušanParadowska‑Gorycka, AgnieszkaCoada, CameliaParamasivan, KalaivaniCoates, DawnParameswaran, JananiCobo-Vuilleumier, NadiaParamio, Jesús M.Cocca, Claudia MarcelaParanjape, AnuragCoccheri, SergioParashar, VijayCocco, NinoParaskevaidis, IoannisCodrici, ElenaParaskevas, George P.Coeffier, MoïsePardo, Juan PabloCoelho, DavidParedes, RobertoCoghlan, GerryParedes-Gamero, EdgarCohen, EhudParekh, AnantCohen, MickaelParent, LucieCohen, PinchasParente, PaolaČokić, VladanParet, ClaudiaColangelo, DonatoParfyonova, Yelena V.Colasante, ClaudiaParga, Juan A.Colasanti, TaniaParhizkar, SamiraColbath, AimeeParimon, TanyalakColeman, JonathanParisi, FrancescaColeman, PaulPark, Eun JeongColetti, DarioPark, Hun-JunColinas-León, Maria TeresaPark, Jae-BongColinge, JacquesPark, Jae-HyungColl, MarPark, JaeyongColl, RebeccaPark, Jong KookCollantes-Estevez, EduardoPark, JoongkyuCollart, Martine A.Park, Jun HyoungCollawn, Sherry S.Park, Kwan-KyuColler, Hilary A.Park, Kyung SikCollins, FraserPark, Mi KyungColmenero, IsabelPark, SophieColombier, PaulinePark, Sun-ahColtro, GiacomoPark, SunyoungColumbano, AmedeoPark, Thomas In-HyeupColussi, ClaudiaPark, YongKeunComandone, AlessandroPark, Young-MinComas-Garcia, AndreuParker, Hugo J.Combettes, LaurentParker, JimComerma-Steffensen, Simon GabrielParker, KrystalComi, CristoforoParkinson, EricComincini, SergioParks, DavidCompagnucci, ClaudiaParks, William C.Conceição, FranciscoParmar, MalinCondello, MariaParnell, EuanCondrat, CarmenParnetti, LucillaConfalonieri, MarcoParoli, MarinoConnaughton, VictoriaParonetto, Maria PaolaConover, CherylParrini, Maria CarlaConradi, LenardParrizas, MarcelinaConstantin-Teodosiu, DumitruParrotta, Elvira ImmacolataContaldo, MariaParrow, Nermi L.Conti, MarcoParrozzani, RaffaeleConti, PioParveen, SadiyaCook, TiffanyParys, Jan B.Coombs, KevinPascale, Rosa MariaCooper, Jonathan B.Pascual, Carmen MaríaCooper, Robin L.Pascual, GloriaCorà, DavidePaslawski, WojciechCoradin, MarielPasquariello, RolandoCoral-Vázquez, Ramón MauricioPasquinelli, GianandreaCord, BrakebuschPassaro, FabianaCordani, MarcoPasseri, BenedettaCordo, Russo RosaliaPasternak, TarasCordoba, ElizabethPastor-Pareja, Jose CarlosCordone, ValeriaPatai, RolandCores, ÁngelPataskar, AbhijeetCormont, MireillePatel, BhoomikaCorrado, AngeliniPatel, Jai SinghCorrea, Diego AlzatePatel, JatinCorreia, SaraPatel, SandipCorrochano, SilviaPatel, ShyamCorsini, MichelaPatel, Sweta B.Cortelazzo, SergioPateras, IoannisCortés, Andrés J.Pathania, AnupCortese, BarbaraPatra, AmiyaCortese, Maria FrancescaPatra, ChinmoyCortese, NinaPatruno, AntoniaCortés-Montero, ElsaPatrussi, LauraCortés-Rojo, ChristianPattappa, GirishCorti, AlessandroPaudel, Keshav RajCorver, Wim E.Paul, DennisCosta, JoanaPaul, SujayCosta, Marina C.Paulo, PaulaCosta, Ryan S.Pavek, PetrCostell, MercedesPavel-Tanasa, MarianaCotella, DiegoPavlichenko, VasiliyCotterill, SuePavlik, Edward J.Cottrell, JessicaPavlov, Dmitrii A.Coulombe, BenoitPawar, Vishakha AnandCouso, InmaculadaPawlas, NataliaCoutant, FrédéricPawliczak, RafalCouvineau, AlainPawłowska, BarbaraCoy-Barrera, EricssonPawłowski, TomaszCoyle, Patricia K.Payne, Sophie C.Craigie, RobertPearlman, EricCraina, Marius L.Pearson, Helen B.Cram, ErinPeça, JoãoCram, Erin J.Pecar, Fonovic UrsaCramer, Chris S.Peche, VivekCravedi, PaoloPeck, Sun H.Crawford, LindseyPedelacq, Jean-DenisCrean, Angela J.Pedemonte, NicolettaCremer, AnjaliPedersen, Per AmstrupCremer, MarionPedro, BullonCrepaldi, TizianaPędzińska-Betiuk, AnnaCrestani, BrunoPeh, GaryCrestani, MaurizioPei, JiayiCreus-Muncunill, JordiPei, ShaopengCrinelli, RitaPeintner, LukasCrisafulli, GiovanniPeired, Anna JulieCrispo, FabianaPejchal, JaroslavCristofani, RiccardoPejler, GunnarCrnkovic, AnaPekary, Albert EugeneCrocetto, FelicePęksa, RafałCrosio, ClaudiaPelagalli, AlessandraCross, MichaelPelchen-Matthews, AnnegretCroteau, DeborahPellicer, PedroCroyle, MariaPellizzon, Cláudia HelenaCruz, Guerrero AlmaPelosi, EmanueleCruz, JorddyPeluzio, Maria DoCruz-Gregorio, AlfredoPeña-Amaro, JoséCserepes, MihalyPeña-Llopis, SamuelCsiszar, AgnesPenate, XeniaCsősz, ÉvaPeng, TianqingÇubuk, CankutPeng, YangCucchiarini, MagaliPeng, YeCucinotta, MaraPenissi, Alicia B.Cucu, DanaPenna, ClaudiaCuesta-Gomez, NereaPenna, FabioCui, JianzhouPennarun, GaëlleCui, JiePenolazzi, LetiziaCui, Mei-zhenPenzo, MariannaCui, WeiPepe, FrancescoCulot, MaximePepe, StefanoCumbo, CosimoPepper, AndreaCuneo, Matthew J.Peracaula, RosaCunha, Filipe M.Peraldi, PascalCurcio, Christine A.Perálvarez-Marín, AlexCurien, GillesPerche, FedericoCurvello, RodrigoPercival-Smith, AnthonyCvekl, AlesPerdriger, AlethCypess, Aaron M.Perego, RobertaCzamara, KrzysztofPereira, GisleneCzarnecka, KarolinaPereira, LeonelCzekała, WojciechPereira, Maurício DosCzembor, ElżbietaPereira, Renato B.Czimmerer, ZsoltPereira, RuiCzop, MarcinPereira, Susana P.Czyz, AnnaPereira-Dutra, FilipeD’Addario, ClaudioPerepechaeva, Maria L.D’Agostino, SilviaPerera, Chamini J.D’Agostino, Vito GiuseppePerera, Nirma D.D’Alò, FrancescoPerera, OmaththageD’Alterio, CrescenzoPérez, Luz M.D’Amico, DanielaPérez, WilliamD’Amico, FedericaPérez-Campo, Flor MaríaD’Amico, RamonaPérez-Castrillón, Jose LuisD’Amuri, AlessandroPérez-Hernández, NuryD’Aniello, EnricoPérez-Luz, SaraD’Aniello, SalvatorePérez-Monter, CarlosD’Antongiovanni, VanessaPerez-Rigueiro, JoseD’Ascenzo, MarcelloPérez-Santos, MartínD’Erchia, Anna MariaPérez-Torres, IsraelD’Ettorre, GabriellaPergolizzi, BarbaraD’Onofrio, NunziaPeritore, Alessio FilippoD’Orazi, GabriellaPerkins, AndrewD’Souza, Rochelle C.Perkins, James RichardD’Ursi, PasqualinaPernet, VincentDa, Cruz PaulaPerni, MicheleDa, Rocha AndrePerpetuini, DavidDababat, AmerPerrais, MichaëlDaban, Joan RamonPerrault, IsabelleDabdoub, AlainPerrella, AlessandroDabrowski, Michal J.Perrier, RomainDada, ReyadPerrin, Florence E.Dahl, RichardPerrotta, CristianaDai, HanchuanPerry, John M.Dai, HuiPerše, MartinaDaisuke, KobayashiPersechino, SeverinoDaisuke, KondohPerumal, Madan KumarDakhlallah, DuaaPerunova, NataliaDakshanamurthy, SivanesanPerveen, AnjumDal Col, JessicaPesce, GiuseppeDal, Prá IlariaPeskin, AlexanderDalCorso, GiovanniPetanidis, SavvasDal-Fabbro, RenanPetar, PujićDalhaimer, PaulPetca, AidaDalm, Simone U.Petcherski, AntonDalvi, SonalPeter, CarlenDamásio, JoanaPeters, ChristianDanforth, DavidPeters, MarcusDani, ChristianPeters, OwenDaniel, PankowskiPetersen, OleDaniele, SimonaPeterson, LisaDanila, Daniel C.Peterson, Stephen J.Danilenko, MichaelPetillo, OrsolinaDanyukova, TatyanaPetit, Patrice X.Danzi, Sara E.Petkov, StoyanDar, Mohd SaleemPetrakis, IoannisDar, WasimPetre, Brînduşa AlinaDarbon, PascalPetrescu, StefanaDarche, FabricePetri, William A.Dariolli, RafaelPetrić-Miše, BrankaDaròs, José-AntonioPetrinovic, Marija-MagdalenaDarszon, AlbertoPetroll, W. MatthewDart, RobinPetros, Timothy J.Darwiche, NadinePetrosyan, AstgikDaryabor, GholamrezaPetrou, PanayiotaDas, AayudhPetrs-Silva, HildaDas, Anibh MartinPetrushanko, IrinaDas, BhabatoshPettinato, GiuseppeDas, DebabrataPeuget, SylvainDas, MalayPeulen, OlivierDas, NeeladrisinghaPeyrl, AndreasDas, Raunak KumarPezzolesi, MarcusDas, RishabhPezzuto, AldoDas, SrjitPfeiler, SusanneDas, UtpalPfister, Astrid S.Das, ViswanathPfrieger, FrankDasari, SubramanyamPhalke, SwatiDass, CrispinPhan, Chia-WeiDaszkowska-Golec, AgataPhan, Thi Tuong VyDatta, MoumitaPhelps, Mitch A.Daubon, ThomasPhilips, Mari-AnneDavenpor, AlexPhilipsen, SjaakDavid, Laurent I. B.Phumee, AtcharaDavid, PiresPi, YuDavid-Cordonnier, Marie-HelenePiao, WenjiDavido, DavidPiasecki, EgbertDavie, JudithPiatek, JacekDavie`re, Jean-MichelPiatti, PieroDavis, PaulPiazzesi, AntoniaDavis, TomPibuel, Matías A.Davletov, BazbekPicard, DidierDavuluri, GangaraoPiccirillo, SilviaDawson, TedPiccoli, MarcoDay, Chi-pingPicconi, BarbaraDay, MatthewPichler, HerbertDe Biase, AlbertoPickering, Raelenede Brevern, Alexandre G.Picone, CarmineDe Candia, PaolaPiekorz, Rolan P.De Carvalho, Katherine Athayde TeixeiraPiemonte, FiorellaDe Castro, FedericaPierantoni, RiccardoDe Deurwaerdère, PhilippePierno, SabataDe Falco, ElenaPierre, RougéDe Falco, MariaPierzchala-Koziec, KrystynaDe Felice, ClaudioPiesik, DariuszDe Francia, SilviaPietropaolo, SusannaDe Geest, BartPignataro, GiuliaDe Groot, EricPignatelli, DuarteDe Luca, CaterinaPikarsky, EliDe Luca, FrancescaPilala, Katerina-MarinaDe Marco, RossellaPilc, AndrzejDe Miglio, Maria RosariaPilch, JanDe Oliveira, JaderPileczki, ValentinaDe Oliveira, Paulo TambascoPilette, CharlesDe Palma, ClaraPiliponsky, Adrian M.De Rosa, LuciaPillai, SnehaDe Rosa, VeronicaPimentel, GustavoDe Rossi, AnitaPina, CristinaDe Sanctis, Juan BautistaPinart, ElisabethDe Souza, Paulo Victor SgobbiPineda, BenjaminDe Souza, WanderleyPinel, KarineDe Stefani, DiegoPinho, DoraDe Waard, VivianPini Prato, AlessioDe, Almeida EdgarPini, Luigi AlbertoDe, Almeida ValériaPinti, MarcelloDe, Amicis FrancescaPinto, Maria JoanaDe, Andrade ClevertonPinto, Sneha M.De, Angioletti MariaPiomboni, PaolaDe, Aro AndreaPion, MarjorieDe, Barros Jean-PaulPioner, Josè ManuelDe, Castro GabrielaPiovesana, RobertaDe, Cesare DarioPipeleers, Daniël G.De, Chiara LetiziaPiperi, ChristinaDe, Ciuceis CarolinaPipino, CaterinaDe, Fabiani EmmaPires, Isabel M.De, Farias RobsonPires, PauloDe, Figueiredo-Pontes LorenaPirkmajer, SergejDe, Giorgi MarcoPiróg, Katarzyna A.De, Gonzalo-Calvo D.Pirtoli, LuigiDe, Graaf PetraPisani, Didier F.De, Grandis MariaPisanu, ClaudiaDe, Haan NoortjePisarev, VladimirDe, La PenaPisconti, AddolorataDe, Leon SmadarPiscopo, MarinaDe, Matos DihogoPishavar, ElhamDe, Melo BrunoPislariu, CatalinaDe, Miguel MariaPissarek, MargitDe, Ninno AdelePistamaltzian, NikolaosDe, Noronha LuciaPistelli, RiccardoDe, Oliveira CarvalhoPistello, MauroDe, PradipPistilli, EdwardDe, RanjitPistorio, ValeriaDe, Rezende ThiagoPitha, JanDe, Rosa FrancescoPittaluga, AnnaDe, Rossi PierrePitts, Todd M.De, Schauwer CatharinaPiwowarski, Jakub PatrykDe, TanimaPizzagalli, Diego UlisseDe, Vries AntoinePlaas, MarioDe, Vries JanPlacer, CarlosDe, Vries MichielPlaczek, William J.Dean, CharlottePlaninšek, OdonDe-Bandt, Jean-PascalPlant, EwanDebanne, DominiquePlatta, HaraldDebnath, RahulPlaza-Díaz, JulioDecaro, NicolaPleckaityte, MildaDecheng, WangPljesa-Ercegovac, MarijaDeCherney, Alan H.Ploner, ChristianDede, SemihaPluth, Janice M.Dedek, KarinPodar, DorinaDeering-Rice, Cassandra E.Pode-Shakked, NaomiDeeth, HiltonPodolski-Renić, AnaDegen, MartinPoirier, Guy G.Degirmenci, AlperenPojda, ZygmuntDegoricija, MarinaPolakowski, Nicholas J.Degrassi, FrancescaPolčic, PeterDekaban, Gregory A.Polesello, CédricDekleva, MilicaPoletto, EdinaDel Fabbro, MassimoPoliard, AnneDel, Campo MartaPöling, JochenDel, Porto PaolaPolis, BaruhDel, Re DominicPolito, RitaDelalle, IvanaPoljakovič, ZdravkaDelanoue, RenaldPoloni, AntonellaDelgado, Evan R.Poloni, Tino EmanueleDelgado, Olquin PaulPolovic, NatalijaDelgado, Teresa C.Polychronakos, ConstantinDelhanty, PatricPoma, AdolfoDelisle, Jean-SébastienPomara, CristoforoDell’Amore, AndreaPomorski, AdamDell’Aversana, CarmelaPomorski, PawełDella Torre, SaraPonce-Vargas, MiguelDella-Morte, DavidPongcharoen, SutatipDelle Cave, DonatellaPoniedziałek-Czajkowska, ElżbietaDellis, OlivierPoniewierka, ElżbietaDelmas, VeroniquePonnayyan, Sulochana SureshDelneste, YvesPonomarenko, ArinaDelprat, BenjaminPons, VincentDemarque, DanielPontarin, GiovannaDemartini, ChiaraPontes, BrunoDe-Miguel, ManuelPonte-Sucre, AliciaDemonacos, ConstantinosPonti, DonatellaDenBesten, PamelaPontifex, MatthewDende, ChaitanyaPontoriero, AntonioDeng, LingxiaoPoole, AlastairDeng, XuewenPoon, IvanDeniaud, AurélienPop, Laurentiu M.Denis, DupoironPop, SevinciDeniz, ErtemPopa-Wagner, AurelDenner, JoachimPopeijus, Herman E.Denton, Travis T.Popelka, HanaDepalma, Ralph G.Popescu, RoxanaDepciuch, JoannaPopov, Yury V.Deprá, Mariany CostaPopović, KostaDerda, Anselm A.Popovic, Zoran V.Derler, IsabellaPopper, Helmut H.Desai, AnandPorcari, Andréia DeDesai, DhruvPoreba, MarcinDesai, PriteshPorosk, LyDesantis, VanessaPorsborg, Simone RiisDesaphy, Jean-FrançoisPortella, GiuseppeDescamps, VincentPosey, Karen L.Deschenes, MichaelPotaczek, DanielDeshmukh, RupeshPotere, NicolaDeshpande, DeepakPotokar, MajaDespotović, SanjaPotter, Pamela E.Detmar, MichaelPoubelle, Patrice E.Dettlaff-Pokora, AgnieszkaPoulain, Fabienne E.Dettlaff-Pokora, Habil. AgnieszkaPourahmad, JalalDey, AbhishekPoyet, Jean-LucDey, Partha NarayanPožgajová, MiroslavaDhakal, SudipPozharskiy, EdvinDhamija, PuneetPozzo, Eleonora DaDhanavade, Maruti JayramPozzo, FedericoDhanushkodi, AnandhPozzobon, MichelaDhanyamraju, Pavan KumarPozzuoli, AssuntaDhar, InduPrabakaran, D. S.Dharmadasa, ThanujaPrabhakaran, Vasantha-SrinivasanDhasarathy, ArchanaPrabhala, Bala KrishnaDhawan, ManishPradeep, SunilaDhingra, RimpyPradhan-Sundd, TirthadipaDhungel, BijayPradotto, Luca GuglielmoDi Agostino, SilviaPrag, Hiran A.Di Benedetto, MélaniePrakash, Babu PhanithiDi Bernardo, GiovanniPrakash, MuthuramalingamDi Cerbo, AlessandroPramanik, ArindamDi Dato, ValeriaPramanik, DibyajyotiDi Donato, MarziaPrange, ReinhildDi Fazio, PietroPrantl, LukasDi Fiore, Maria MaddalenaPranzini, EricaDi Gennaro, FrancescoPrasanna, ChinmayiDi Giacomo, ClaudiaPrasse, AntjeDi Gioacchino, MarioPrathipati, PhilipDi Gioia, DianaPrats, Anne-CatherineDi Liddo, RosaPrattichizzo, FrancescoDi Lorenzo, FlavianaPrayer, DaniellaDi Malta, ChiaraPrchal, Josef T.Di Miceli, MathieuPrice, PatriciaDi Nardo, DarioPriksz, DanielDi Paola, RosannaPrince, Henry MilesDi Pietro, NataliaPrincivalle, AlessandraDi Resta, ChiaraPrinsloo, EarlDi Ruscio, AnnalisaPritchard, Michele T.Di Scala, CoraliePrivette, Vinnedge LisaDi Sole, FrancescaPriya, SmritiDi Stefano, CarlaProbert, LesleyDi, Benedetto GiuliaProcaccini, ClaudioDi, Buduo ChristianProcopio, Francesco AndreaDi, Giorgio ErosProietti, De SantisDi, Liegro ItaliaProikas-Cezanne, TassulaDi, Nottia MichelaProkesch, AndreasDi, Polo AdrianaProkopiou, KaterinaDi, Stefano BarbaraPromponas, VasilisDi, Tommaso LucaProneth, BettinaDiamantidis, Michael D.Pronina, IrinaDiao, YaruiProsperi, EnnioDias, CelesteProsperi, Jenifer R.Dias, SérgioPrószyński, Hab. TomaszDíaz , José FernandoProvot, SylvainDiaz, Miguel AngelPrud’homme, Gérald J.Díaz-Flores, LucioPruschy, MartinDíaz-Quintana, AntonioPrzemyslaw, BorysDíaz-Ruiz, CarmenPrzybyło, MałgorzataDiederichs, SolvigPrzystupski, DawidDieleman, Jan M.Psatha, NikolettaDiener, MartinPu, YongDieni, CristinaPuca, AnnibaleDiercks, Björn-PhilippPucéat, MichelDietl, PaulPuente-Rivera, JonathanDigilio, Filomena AnnaPuga, Guilherme MoraisDigvijay, KumarPuglia, GiuseppeDijkstra, Johannes M.Pugliese, NicolaDimakopoulos, GeorgiosPula, GiordanoDimitriadis, GeorgePullen, NicholasDinca, ValentinaPunia, HimaniDing, BaojinPurcell, ErinDing, Bi-SenPurgatorio, RosaDing, Ling-WenPuri, Basant K.Ding, PengfeiPurohit, PurviDing, WeihuaPuscasiu, LucianDini, ValentinaPusch, StefanDinischiotu, AncaPüschel, GerhardDiomede, FrancescaPushalkar, SmrutiDiotel, NicolasPushparaj, SamuelDiretto, GianfrancoPuskulluoglu, MiroslawaDirice, ErcumentPyle, GlenDistl, OttmarPyne, NigelDittenhafer-Reed, KristinQaisar, RizwanDivisato, GiuseppinaQamar, RafiDiwaker, DrishyaQasim, SumeraDiwan, AbhinavQayyum, Mohammad ZuhaibDixon, Ian M. C.Qazi, Izhar HyderDjabali, KarimaQi, DakeDjavadian, RuzannaQi, HuayuDjavaheri-Mergny, MojganQi, XiaofengDjerada, ZoubirQi, YifengDjukanovic, RatkoQi, YitaoDjurasevic, SinisaQian, HongDludla, Phiwayinkosi V.Qian, XiuqingDo, Carmo SoniaQiang, LiDobke, Marek K.Qiang, YuhaoDoblas, AnaQiao, JianlinDobner, JochenQin, FujunDobre, OanaQin, JuanDobrowolny, GabriellaQin, WeiDobrowolski, PiotrQiu, BinDocea, Anca OanaQiu, FangfangDoceul, VirginieQiu, HongyuDoczi, JuditQu, YanjiDodi, GianinaQuagliariello, VincenzoDogra, SamritaQuaglino, PietroDohan, OrsolyaQuang, Le BachDohmen, R. JurgenQuaresma, Juarez Antônio SimõesDokládal, LadislavQuartu, MarinaDolan, Brian P.Quayle, JohnDolivo, DavidQueiroz, GlóriaDolmatov, Igor Yu.Quek, CameliaDołowy, KrzysztofQuesada, Andrés E.Domene, HoracioQuijano, CeliaDominguez, MariaQuintana, AnitaDominguez-Meijide, AntonioQureshi, Asaf A.Domoki, FerencRab, AndrásDomvri, KellyRabanal, Ruiz YoanaDonati, GiacomoRabani, VahidehDonato, Rosario FrancescoRabelo, KíssilaDoncel-Pérez, ErnestoRabhi, NabilDong, KunzheRabinovich-Nikitin, InnaDong, XiangshuRabson, Arnold B.Dong, XianpingRacay, PeterDonizetti, AldoRachek, LyudmilaDonkers, Joanne M.Rachubinski, RichardDonninger, HowardRacioppi, LuigiDonoso, PaulinaRacke, MichaelDoody, Karen M.Raczyńska, Katarzyna DorotaDopytalska, KlaudiaRada, PatriciaĐorđević, AleksandarRadaelli, EnricoDormiani, KianoushRadenkovic, MiroslavDormoy, ValerianRadhakrishnan, RamalingamDoronzo, GabriellaRadin, MassimoDorszewska, JolantaRadmilović-Radjenović, MarijaDossena, SilviaRae, Roh CheongDossi, ElenaRafati, NimaDötsch, VolkerRagan, IzabelaDotti, SilviaRageul, JulieDougan, David A.Raghav, Pawan KumarDoulames, Vanessa M.Raghunathan, RakshaDoulberis, MichaelRagino, Yuliya I.Doventas, AlperRagni, EnricoDowd, Kimberly A.Ragnini-Wilson, AntonellaDowd, Patrick F.Ragusa, AndreaDowling, JenniferRahaghi, Franck FarzadDoxakis, EpaminondasRahim, FazalDragomir, MihneaRahman, Anas M.Dragonieri, SilvanoRahman, ArshadDrakos, EliasRai, Krishan MohanDrakulić, DunjaRai, Mani RatnamDratkiewicz, EwelinaRai, VikrantDreier, RitaRaica, Marius L.Drenjančević, InesRaimondo, DiegoDrube, SebastianRaimondo, StefaniaDrulis-Fajdasz, DominikaRaj, ThirumalDrummond, CatherineRaja, GanesanDrury, Stacy S.Raja, Iruthaya Pandi SelestinDu, MeijunRaja, RameezDu, ShaojunRaja, RathinamDuan, JialeiRajala, Raju V. S.Duarte, Márcio S.Rajasekaran, ArunDubé, MathieuRajendran, Ramya LakshmiDubey, AnamikaRajfur, ZenonDubiel, MariuszRajpurohit, SurendraDubinnyi, Maxim A.Rajwa, BartekDubinski, DanielRakhymzhan, AsylkhanDubovy, PetrRakowski, TomaszDubrova, YuriRamachandra, Rao SriganeshDubuquoy, LaurentRamadas, NirupamaDuchi, SerenaRamadesikan, SwethaDuda-Madej, AnnaRamadori, GiulianoDudas, JozsefRamaiahgari, Sreenivasa C.Dudzicz, SylwiaRamalho-Santos, JoãoDueck, AnneRamamoorthy, AyyalusamyDugbartey, George J.Ramamoorthy, PrabhuDuguez, StephanieRamanathan, PriyaDuitman, Jan WillemRamasamy, RajeshDulak, JózefRamatchandirin, BalamuruganDunaief, JoshuaRamazanov, BulatDuncan, Melinda K.Ramberger, MelanieDuncan, Raymond ScottRamchandran, RamaniDundar, MunisRamírez Hidalgo, Cristina M.Dunlop, ElaineRamírez-Bribiesca, J. EfrénDunoyer-Geindre, SylvieRamjeesingh, RaviDuong, Thuc-HuyRamkumar, Kunka MohanramDupin, IsabelleRamli, RoszalinaDupree, Jeffrey L.Ramos, AntonioDuran, Bruno OliveiraRamos-Tovar, ErikaDuran-Aniotz, ClaudiaRampias, TheodorosDuranti, ClaudiaRamsden, DavidDuranti, GuglielmoRamsköld, DanielDuraturo, FrancescaRanawade, AyushDurgo, KsenijaRangachari, ManuDurham, Benjamin H.Rangan, ParimalanDurham, EmilyRangrez, Ashraf YusufDurmaz, KorayRanieri, GirolamoDutta, AvikRanjan, KishuDutta, MainakRao, KrithikaDutta, PranabanandaRao, RakeshDutta, Raghbendra KumarRao, SmithaDutta, Shubha RanjanRao, TejeshwarDutta, SumanRao, VidhyaDvořáčková, MartinaRapino, CinziaDvorak, ZdenekRappa, FrancescaDwivedi, MiteshRaqib, RubhanaDyachuk, VyacheslavRashid, FahadDyląg, MariuszRashmi, RashmiDyleva, YuliaRaskov, Hans HenrikDyson, Paul J.Rasmussen, AndersDzięgiel, PiotrRasola, AndreaEaly, AlanRassoulzadegan, MinooEandi, ChiaraRasul, SazanEastman, AlisonRathee, VikramjitEberlé, DelphineRather, Irfan A.Ebert, FranziskaRathinasabapathy, AnandharajanEbihara, LisaRathore, AtulEbinu, Julius O.Ratnayaka, J. ArjunaEbstein, FrédéricRattan, SureshEckert, JeffreyRatushnyy, AndreyEckhardt, MatthiasRavaglia, SabrinaEckhart, LeopoldRaval, AmiEdelmann, ElkeRavanidis, StylianosEeckhoute, JérômeRavara, SofiaEfentakis, PanagiotisRavel, CeliaEferl, RobertRavens, SarinaEfthimia, CotouRavera, SilviaEftimie, RalucaRavid, TommerEgger, GerdaRavindra, P. VeerannaEhara, NatsuhikoRavindranath, MepurEhler, EdvardRavishankar, PrashanthEhnert, SabrinaRavn, PenilleEhrlich, Michelle E.Ravnik, JanezEichmüller, Stefan B.Rawat, AnoopEiges, RachelRawat, KavitaEini, OmidRay, SwapanEiras, SoniaRaychaudhuri, PradipEisenberg, TobiasRaymond, AndreaEisenga, Michele F.Raz, ItamarEisenstat, DavidRaza, AfsheenEkeuku, Sophia OgechiRaza, HaiderEkker, MarcRazidlo, Gina L.Ekpenyong, Andrew EdetRazin, EhudEl Agha, ElieRe, Cecconi AndreaEl Hiba, OmarReali, EvaEl Khatib, MohammadReane, Denis VecellioElahi, ShokrollahRebbeck, RobynEl-Amri, ChahrazadeRebelo, SandraElaswad, AhmedRebolledo, Daniela L.El-Bahrawy, MonaRebollo-Hernanz, MiguelElbouzidi, AmineRecchia, AlessandraElchaninov, AndreyRechciński, TomaszEl-Deiry, Wafik S.Reddehase, MatthiasElena, A. JonesReddy, Marpadga A.Elena, EnachiReddy, Narsa M.El-Esawi, Mohamed A.Reddy, P. HemachandraElewa, Yaser HosnyReddy, SangeethaEl-Gewely, Mohamed RaafatRedmer, TorbenElhasid, RonitRedondo, Maria J.El-Huneidi, WaseemReed-Geaghan, Erin G.El‐Husseiny, RaniaRegal, JeanEljaszewicz, AndrzejReggio, AlessioEl-Jawhari, Jehan J.Rehman, GauharElkady, AbeerRehman, NajeebElkamhawy, AhmedRehman, Saeed UrElkhenany, HodaRehman, ShaziaElks, PhilipReich, AdamElleder, DanielReichardt, Sybille D.Eller, KathrinReichart, BrunoElli, StefanoReinach, Peter SolEllinger, IsabellaReinartz, SilkeEllis, Ronald J.Reinders, JörgEllwanger, Joel HenriqueRelic, BiserkaEl-Magd, MohamedRelloso, Miguel S.Elmetwally, MohammedRemigante, AlessiaElmonem, Mohamed A.Ren, HongyanEl-Nagar, MehrezRen, JunElnahri, AymanRen, Pei-GenElnesr, ShaabanRen, TianhengEl-Ramady, HassanRen, XiangEl-Shal, AmalRenate, KunertElsner, MatthiasRene, SchrammEl-Soda, MohamedRenovato-Martins, MarianaEltit, FelipeRenzini, AlessandraEltzschig, HolgerResende, RosaEmamgholipour, SolalehRestaino, StefanoEmran, Talha BinReutelingsperger, ChrisEmri, EszterReverchon, MaximeEnany, ShymaaRey, LuisEndres, ThomasReyes, Carina AndreaEnger, RuneReyes, José LuisEngevik, ​AmyReynolds, PaulEngevik, Kristen A.Reynolds, Timothy M.Enguita, Francisco J.Rezaee, FaribaEnko, DietmarRezaie, JafarEnosawa, ShinRezende, Izabela MauricioEnzmann, VolkerRezende, Rafael M.Eom, Young WooRezsohazy, ReneEppenberger-Castori, SerenellaRhaman, Mohammad SaidurEppler, ElisabethRhee, SangmyungErb, Holger Hans HermanRiabowol, KarlErben, PhilippRiancho, José AntonioErcan-Sencicek, Adife GulhanRiaz, MuhammadErdal, EsraRiazuddin, SheikhErdődi, FerencRibaldone, Davide GiuseppeEriksson, UlfRibalta, JosepErjavec, Gordana NedicRibatti, DomenicoErlandsson, LenaRibeiro, LuísErmakov, EvgenyRibeiro, Vanessa RochaErmakova, OlgaRibeyre, CyrilErmund, AnnaRicardo, Dinis-OliveiraErnst, Peter B.Ricchiuto, PieroErrasti-Murugarren, EkaitzRicci, Angela DaliaEscalante, RicardoRicciardi, LuisaEscate, RafaelRicciardi, Maria RosariaEscriva, HectorRicciotti, EmanuelaEscudero, JoséRichard, PeterEsmaili, MansooreRichards, DylanEsnault, StephaneRichardson, Ricardo M.Espejo-Mojica, Angela JohanaRichardson, SimonEspéli, MarionRichardt, DoreenEspinosa, LluisRichter, WiltrudEspinoza-Sánchez, Nancy AdrianaRideout, HardyEsposito, GennaroRiegger, JanaEsposito, MassimilianoRiehle, Michael A.Essa, MohamedRiemann, DagmarEssaidi-Laziosi, ManelRiesen, Walter F.Esseltine, JessicaRiessland, MarkusEssers, Marieke A. G.Riethmacher, DieterEstrada, Jose L.Riethman, Harold C.Ethiraj, PurushothRigalli, Juan PabloEttensohn, CharlesRigoni, MichelaEttl, TobiasRiillo, CaterinaEtxebeste, OierRimondini, LiaEulenburg, VolkerRinaldi, CarloEvangelou, KonstantinosRinaldi, CiroEvans, CameronRinaldi, FabioEvans, GarethRinnerthaler, MarkEvavold, Charles LeeRintz, EsteraEvrard, Solène M.Riolo, GiuliaExbrayat, Jean-MarieRios-Colon, LeslimarEyase, FredRiparbelli, Maria GiovannaEyraud, DanielRipoli, AndreaFábián, AttilaRippe, CatarinaFabian, ZsoltRitchie, Helena H.Fabijanić, DamirRitter, AndreasFabrin, Thomaz MansiniRitva, TikkanenFabris, VictoriaRius-Pérez, SergioFacchin, FedericaRiuzzi, FrancescaFacciorusso, AntonioRiva, FedericaFachini, Roberta MariaRivera Del Álamo, Maria MontserratFaenza, IreneRivero-Müller, AdolfoFafián Labora, Juan AntonioRivero-Ríos, PilarFaggioli, FrancescoRivest, SergeFagioli, FrancaRizvi, FatimaFagone, PaoloRizwanullah, Md.Fahad, ShahRizzo, AlessandroFaheem, Marwa S.Rizzo, PaolaFahimfar, NoushinRizzo, RiccardoFainsod, AbrahamRizzolo, PieraFaiola, FrancescoRo, HyunjuFairless, RichardRobert, PhillipeFairweather, DeLisaRoberts, JoAnn S.Fais, StefanoRobles-Valero, JavierFaissner, AndreasRocco, LuciaFait, ThomasRocha-Mendoza, IsraelFaivre, AnthonyRochani, Ankit K.Faiyue, BualuangRoche, BenjaminFajkus, JiříRochfort, KeithFakhfouri, GoharRöder, StefanFalchetti, AlbertoRodrigo, Juan P.Falcioni, RenanRodrigo, LuisFalcone, SestinaRodrigues, António SebastiãoFalfan-Valencia, RamcesRodrigues, Márcia T.Falk, MartinRodrigues, Robim M.Fall, Mamadou LamineRodríguez Alvarez, JoséFalsini, BenedettoRodríguez, AmaiaFalzone, LucaRodríguez, IsabelFan, Guo-ChangRodríguez-Acebes, SaraFan, HuihuiRodríguez-Barbero, AliciaFan, JiabingRodríguez‐Fdez, SoniaFan, JingyuRodríguez-Fernández, José LuisFan, WenqiangRodríguez-León, JoaquínFan, XingjunRodriguez-Monroy, MarcoFan, XudongRodríguez-Pallares, JannetteFan, YujiangRodriguez-Perales, SandraFancher, Ibra S.Rodríguez-Perálvarez, ManuelFandrey, JoachimRodríguez-Pérez, Ana I.Fanelli, Mariel AndreaRodríguez-Reyna, Tatiana S.Fang, LekunRodríguez-Rocha, HumbertoFang, PingRoduit, RaphaëlFang, QinRoedel, FranzFang, QingmingRoelen, BernardFang, Su-ChiungRoessler, RafaelFantin, AlessandroRoger, LaurélineFantone, SoniaRogers, Gregory C.Farach-Carson, Mary C.Rogers, IanFaraj, MayRogers, Robert S.Faralli, Jennifer A.Rognoni, EmanuelFaraoni, PaolaRognoni, PaolaFaraonio, RaffaellaRogobete, Alexandru FlorinFaraz, AhmadRoh, Gu SeobFarhan, MohammadRohden, FrancieliFarhana, AishaRoi, AlexandraFaridi, RabiaRojalin, TatuFarina, AntonioRojas, ÁngelaFarinha, Carlos M.Rojas, Diego A.Fariss, RobertRojo, Jose M.Farmer, Patrick J.Rojo, Maria AngelesFarooqi, Ammad AhmadRojo-Alba, SusanaFarsetti, DanieleRokaya, DineshFasolato, CristinaRolandsson Enes, SaraFasolino, InesRolfe, RebeccaFassl, AnneRoll, PatriceFattizzo, BrunoRolla, GiovanniFaviana, PinucciaRoman, YoussefFayed, AhmedRomaniello, RominaFazekas, BarbaraRomano, MaurizioFazio, GraziaRomano, NiclaFedak, PaulRomanov, YuriFedele, MonicaRomão, LuísaFehr, AnthonyRomero, Francisco J.Fei, MiaoRomorini, LeonardoFei, XiangRonacher, KatharinaFeigenspan, AndreasRonald, RoepmanFekete, TündeRonca, RobertoFelli, Maria PiaRonca, ShannonFeloukidis, ChristosRonchi, AntonellaFelthaus, Oliver HeinrichRonchi, GiuliaFendt, MarkusRondeau, PhilippeFeng, HaidongRonnberg, ElinFeng, Han-PingRonnekleiv-Kelly, Sean M.Feng, YanpingRopka-Molik, KatarzynaFeng, YuliangRoque, CláudioFeng, YunRos, JavierFenouil, TanguyRos, UrisFenske, StefanieRosa Dias, JulianaFeo, SalvatoreRosa, Julia M.Ferbeyre, GerardoRosa, Juliana M.Fereira, Garrudo FábioRosado, Maria ManuelaFeresin, Rafaela G.Rosati, ElisaFerino, AnnalisaRosati, LuigiFernandes, Elizabeth S.Ros-Bernal, FranciscoFernandes, GustavoRoscini, LucaFernandes, JanainaRose, Christine R.Fernandes, Maria HelenaRose, ChristopherFernandes-Santos, CarolineRose, NjeminiFernández Rodríguez, AmandaRosenthal, Ken S.Fernández, Álvaro F.Rosetti, FlorenciaFernandez, MercedesRosse, CarineFernandez, Vanesa MartinRossi, Carlo MariaFernández-Alvarez, ClaraRossi, DanielaFernandez-Barrena, Maite G.Rossi, PellegrinoFernandez-Botran, RafaelRossignol, JulienFernandez‐Caldas, EnriqueRostami-Nejad, MohammadFernández-Fernández, DiegoRoszek, KatarzynaFernández-Medarde, AlbertoRoszkowska, Anna MariaFernández-Rojas, BereniceRoth, MichaelFernández-Sáiz, VanesaRothbauer, MarioFernández-Tomé, María DelRothe, MichaelFernandez-Tussy, PabloRotstein, NoraFerrai, CarmeloRotti, Pavana G.Ferrara, FrancescoRoumeliotis, Theodoros I.Ferrara, GiovanniRousset, RaphaëlFerrara, NicolaRoux, SophieFerrari, PaolaRoverso, MarcoFerraro, ElisabettaRoviello, GiandomenicoFerraro, SimonaRowlands, David J.Ferraz, Maria PiaRoy Choudhury, SwarupFerreira, Carolina de AguiarRoy, BipradasFerreira, FernandoRoy, MiltonFerreira, Helena LageRoy, Nand K.Ferreira, João AugustoRoy, SaptarshiFerreira, JorgeRóżalski, MarcinFerreira, LeonardoRozenberg, JulianFerreira, Luiz E.Rozza, ArianeFerreira, RodrigoRuan, JingjunFerreira-da-Silva, FranciscoRuaro, BarbaraFerrero, GiulioRubart, MichaelFerretti, EmanuelaRubelj, IvicaFerrini, FrancescoRubino, ElisaFerro, Emer SuavinhoRubinstein, MenachemFerro, RiccardoRubio, Miguel A.Ferrucci, MichelaRubio-Guerri, ConsueloFessler, Richard G.Rubio-Tomás, TeresaFesus, LaszloRubtsov, Yury P.Feurstein, SimoneRubtsova, Maria P.Fialová, AnnaRuden, Douglas M.Fiaschi, TaniaRudnitskaya, EkaterinaFichman, YosefRudolf, EmilFields, JerelRudolf, RuedigerFierro, H. AngélicaRuffini, Pier AdelchiFigg, WilliamRufino-Palomares, Eva E.Figueiredo, BonaldRui, LixinFigueroa, E.Ruijtenberg, SuzanFiladi, RiccardoRuiz, AsierFiliou, MichaelaRuiz, EnricoFilipovic, DraganaRuiz, Felix A.Filipovic, MiodragRuiz, IsaacFilipowska, JoannaRukavina, Mikusic NataliaFilippi, SilviaRumboldt, ZvonkoFilipponi, DoriaRupert, AbeleFilippou, Panagiota S.Ruperto, González-PérezFilosa, JessicaRuscica, MassimilianoFiltz, TheresaRusenova, N.Finazzi, DarioRusina, RobertFine, NoahRusmini, PaolaFinelli, CarloRussell, RodFinicelli, MauroRusso, DomenicoFink, DieterRusso, EthanFink, GeorgeRusso, GiancarloFink, Jodok MatthiasRusso, Roberto R.Finkenzeller, GünterRusu, Laura-CristinaFinnemann, Silvia C.Ruszkowska-Ciastek, BarbaraFinsterer, JosefRuth, E. CarmichaelFiona, LyngRutkowski, Joseph M.Fiorentino, VincenzoRuzzene, MariaFioriniello, SalvatoreRyan, Aimee K.Fioriti, LuanaRychkov, GrigoriFiorotto, RominaRyffel, BernhardFirsov, MikhailRyskalin, LarisaFischer, AndreasSaada, AnnFischer, ItzhakSaadé, NayefFischer, JudithSabapathy, KanagaFischer, Michael B.Sabarwal, AkashFischer, SilviaSabatino, LauraFischer, UlrikeSabir, ZulqurnainFisher, Joseph A.Sabol, MajaFitzGerald, DavidSacco, AntonioFitzgerald, JuliaSacco, FrancescaFiyaz, AbdulSaccone, SalvatoreFlagg, ThomasSacerdoti, FlaviaFleischer, SharonSachinidis, AgapiosFleischhacker, AngelaSaczko, JolantaFlinn, Barry S.Sadanandappa, Madhumala K.Flora, Gagan D.Sadayappan, SakthivelFlorán, BenjamínSadhukhan, PritamFloriano, Juliana FerreiraSaeed, AliFodor, JánosSaeedi, SoheilFoecking, MelanieSaeednejad, Zanjani LeiliFoglieni, ChiaraSaettone, AlejandroFojtová, MiloslavaSaez, BorjaFok, K. L.Safar, JiriFoley, AnnSaga, RyoFollenzi, AntoniaSagnelli, CaterinaFomina, Alla F.Saha, BiswarupFondufe-Mitterndorf, YuvonneSaha, ShekharFonseca-Alves, Carlos EduardoSahgal, PranshuFontaine, VincentSahin, ErgunFontana, RosaSahu, Bidya DharFontanesi, FlaviaSaika, HiroakiFontenla, Josè ÁngelSaini, SanjayForghanifard, Mohammad MahdiSaint-Pol, JulienForis, VasileSaito, MasahiroFornari, Caprara AnaSaito, ShokoFornasaro, StefanoSaito, YouheiForoncewicz, BartoszSaito, YukihiroForrester, Lesley M.Saitoh, MasaoForsburg, SusanSaitoh, ShinjiForsdyke, DonaldSajek, MarcinFort, Patrice E.Sak, Jarosław J.Fort, PhilippeSakamuro, DaitokuForte, MaurizioSakanoue, IchiroFortschegger, KlausSakasai-Sakai, AkikoFoster, Kenneth R.Sakata, NaoakiFotheringham, Amelia K.Sakhaee, KhashayarFouassier, LauraSakkas, Lazaros I.Fouda, AbdelrahmanSakowicz- Burkiewicz, MonikaFourriere, LouSakwe, AmosFox, Howard S.Sala, GessicaFracasso, GiulioSala, Lucia LaFraineau, SylvainSałagacka-Kubiak, AleksandraFrancelin, Rovarotto CarolinaSalama, SamirFrancesco, PivaSalamone, MonicaFrancesco, PuppoSalazar, Luis A.Francesconi, SaraSalbach-Hirsch, JulianeFrancis, HeatherSaldova/Fahey, RadkaFrancischetti, IvoSaleh, TareqFrancisco, Julio CesarSalerno, CarloFrancisco, RiveroSaliba, YouakimFrancisco, TâniaSalido, MercedesFrancisco, VeraSalikhova, DianaFrancisco-Morcillo, JavierSalinas, FranciscoFranco, AlessandraSalinska, ElzbietaFranco, AntoniettaSallustio, FabioFranco, OmarSalman, MootazFranco, PierfrancescoSalopek-Sondi, BrankaFrancolini, GiulioSalta, SofiaFrank, SašaSalton, StephenFranzoni, FerdinandoSaluja, RohitFraschini, RobertaSalvati, EricaFraser, LeylandSalvi, Prafull SalviFratelli, MaddalenaSalvi, SilviaFrati, AlessandroSalzmann, ManuelFratini, EmilianoSamaja, MicheleFrau, RobertoSamaranayake, Chinthaka B.Free, StephenSamarasinghe, Amali E.Freeman, SallySamarzija, IvanaFreen-van, Heeren JulianSamidurai, ArunFreichel, MarcSammar, MareiFreire-de-Lima, Celio GeraldoSampaio-Marques, BelémFrémont, StéphaneSanada, FumihiroFrench, CurtisSanadgol, NimaFrey, BenjaminSanak, MarekFreyschlag, Christian F.Sanches, PauloFrieden, MaudSánchez, Del PinoFriedler, ShevachSánchez, Maria DeFriedman, Alan D.Sanchez-Alcazar, JoseFriel, ClaireSánchez‐Bueno, FranciscoFriemel, JulianeSánchez-Collado, JoséFrietsch, ThomasSánchez-Madrid, FranciscoFrigo, LúcioSánchez-Margalet, VíctorFrigy, AttilaSánchez-Martín, JavierFrisch, Steven M.Sánchez-Sáez, XavierFritsche, EllenSancisi, ValentinaFröhlich, FlorianSandlesh, PoorvaFrøkjær-Jensen, ChristianSandra, Martinez JarquinFronczek, RolfSaneyasu, TakaokiFrondoza, CarmelitaSangiuolo, FedericaFrontiñán-Rubio, JavierSanjeewa, K. K. AsankaFrontini, AndreaŞanlıbaba, PınarFrossi, BarbaraSano, AkitoshiFrostell, ClaesSano, TeruoFrühbeck, GemaSansone, LuigiFu, GuodongSantacruz, Varela AmalioFu, JidongSantagostino, MarcoFu, ShaozhiSantamaria, Patricia G.Fu, ShuyuSantarelli, RobertaFu, TaoSantarsiero, AnnaFuchs, GabrieleSántha, PéterFuchs, TomaszSanthekadur, Prasanna KumarFuchshofer, RudolfSantiago-Tirado, Felipe H.Fuentes-Viveros, LidaSantilli, FrancescaFuhrmann, StephanSantocanale, CorradoFujii, KazuyasuSantos, conceiçãoFujii, YasuhisaSantos, DanielFujii, YukiSantos, DulceFujimoto, KiyohideSantos, Jana Sopková-De OliveiraFujinaga, KohSantos, JoãoFujisawa, KoichiSantos, MarianaFujishima, HiroshiSantos, Raquel DeFujita, MasayukiSantos-Coquillat, AnaFujita, Y.Santos-Costa, QuirinaFujita, YuichiSantovito, GianfrancoFujiwara, KeigiSantpere, GabrielFujiwara, YasuhiroSantulli, GaetanoFujiyoshi, KenjiSanyal, IndraneelFukagawa, TatsuoSanyal, TanmoyFukasawa, TakemichiSapkota, ArjunFukuchi, Ken-ichiroSapkota, KiranFukui, HirokazuSapozhnikov, AlexanderFukumoto, YoshihiroSarantopoulos, StefanieFukunaga, KohjiSaravanan, Konda ManiFukuoka, HideokiSaretzki, GabrieleFulle, StefaniaSarkar, Bhattacharya SayantaniFuller, BryanSarkar, KoustavFunk, Kristen E.Sarkozy, MartaFunk, RichardSarmento-Ribeiro, Ana BelaFurber, KendraSarode, SachinFurmanczyk, EwaSartini, FrancescoFurukawa, YusukeSarul, MichałFuschillo, SalvatoreSarver, AaronFusco, Francesca RomanaSasai, NoriakiFusco, RobertaSassi, CelesteFusz, KatalinSassi, YassineFuszard, MattSastre, LeandroFutrega, KathrynSathish, MuthuGaballah, MohammadSatish, LathaGabazza, Esteban C.Sato, AkiraGabbia, DanielaSato, HideyoGaber, TimoSato, ShuzoGabert, JeanSato, TakashiGabriel, IwonaSattler, WolfgangGabriele, MulthoffSauer, HeinrichGabrielli, BrianSauter, EdwardGacem, SabrinaSavatier, PierreGadad, ShrikanthSavic, Lynn JeanetteGadekallu, Thippa ReddySawai, SatoshiGadhave, KundlikSawicka, AnnaGadoth, AviSayako, KatadaGaetano, CarloSayed, Ibrahim M.Gaffke, LidiaSayre, Richard T.Gaffo, Angelo L.Sazonova, Margarita A.Gagat, MaciejScaglione, MichelangeloGage, MatthewScalco, ElisaGagliano, NicolettaScanu, AnnaGagliardi, StellaScaramuzzino, ChiaraGaglio, DanielaScarcia, PasqualeGagos, SarantisSchaaf, Marcel J. M.Gahmberg, Carl G.Schäbitz, Wolf-RüdigerGaiarsa, Jean-LucSchäfer, KatrinGaidin, Sergei G.Schaniel, ChristophGaigneaux, AnthoulaScharf, AndreaGaino, FrancescaSchatten, HeideGajavelli, ShyamSchattner, Mirta A.Gajewska, AlinaSchellenberg, MatthewGalagudza, Mikhail M.Schelling, JeffreyGalati, NickSchelling, Jeffrey R.Galfalvy, HangaSchemmert, SarahGalietta, LuisScher, MarkGáliková, MartinaSchermer, BernhardGalindo, AntonioSchetters, Sjoerd T.Galindo, Máximo IboSchieweck, RicoGalizzi, GiacomaSchilling, OliverGalkina, SvetlanaSchilling, StephanGalkina, Svetlana A.Schimpl, MarianneGall, Maude LeSchippa, SerenaGallardo, EduardSchizas, DimitriosGallardo, GilbertSchlee, Villodre EmillyGallardo, M. EstherSchlesinger, MartinGallart-Palau, XavierSchlicker, EberhardGallay, PhilippeSchmalzing, MarcGallego, MonicaSchmauss, DanielGallelli, LucaSchmidt, ConstanzeGallemí, MarçalSchmidt, Mirko H. H.Galletti, CosimoSchmitt, FernandoGalli, AndreaSchmitz, FrankGallo, GaetanoSchmollinger, Stefan R.Galluzzo, PaolaSchneckenburger, HerbertGalupa, RafaelSchneider, DirkGaluska, Sebastian P.Schneider, JochenGalvagni, FedericoSchneider-Maunoury, SylvieGalvan, Daniel L.Schoenhagen, PaulGama-Carvalho, MargaridaSchön, JenniferGamarra, Lionel FernelSchönthal, Axel H.Gamarra-Luques, Carlos DiegoSchöpfer, JuttaGambaryan, StepanSchossleitner, KlaudiaGamberi, ChiaraSchreckenberg, RolfGameiro, Steven F.Schröder, AgnesGanapathy, SuthakarSchubert, FrankGanassi, MassimoSchubert, MarioGandhi, Neha S.Schuchardt, MirjamGandía, LuisSchueler, JuliaGanesan, KumarSchukken, Klaske M.Gangaraju, RajashekharSchultz, VerenaGanguly, PayalSchulz, LauraGanji, RakeshSchulze-Makuch, DirkGantenbein, AndreasSchusser, Gerald FritzGantenbein, BenjaminSchutte, StaceyGanusov, VitalySchwalbe, Ruth A.Gao, BoSchwartz, Elisabeth FerroniGao, ChaoSchwarz, MarkusGao, FuqiangSchweitzer, JeffreyGao, HuichangSchwinger, WolfgangGao, JianSchwob, EtienneGao, PeisongSciaccaluga, MiriamGao, QiSciamanna, GiuseppeGao, ShouguoSciani, Juliana MozerGao, XiangScicali, RobertoGarab, GyozoScicluna, Brendon P.Garattini, EnricoScifo, EnzoGarayoa, MercedesScimone, ConcettaGarcia Cozar, Francisco JoseScomparin, AnnaGarcía De Frutos, PabloScorza, TatianaGarcia, AntonioScott, EricaGarcía, Del CañoScupoli, Maria TeresaGarcía, Foncillas JesúsScuteri, AriannaGarcía, GabrielaScuteri, DamianaGarcía, Heathcliff DoradoSeabra, CatarinaGarcia, Jéssica LeiteSeabra, Catarina MoraisGarcia, MagdalenaSeabra, MiguelGarcia, MichelleSebastian, MartinGarcía, MontserratSebestjen, MiranGarcía, Rivero JuanSebestyèn, AnnaGarcía-Arrarás, José E.Secondo, AgneseGarcía-Ayuso, DiegoSedyh, SergeyGarcía-Barrado, María JoséSeetoh, Wei GuangGarcía-Caballero, MelissaSegatto, MarcoGarcia-Etxebarria, KoldoSegura-Aguilar, JuanGarcía-Flórez, LuisSegura-Bayona, SandraGarcía-García, AndrésSehgal, Pravin B.Garcia-Macia, MarinaSeifalian, AlexanderGarcía-Miranda, PabloSeifert, GeraldGarcía-Sanz, José A.Seiler, Magdalen J.Garcia-Suarez, OliviaSeipel, KatjaGardiner, Katheleen J.Seki, EkihiroGardoni, FabrizioSeki, NaohikoGarg, DeepikaSeki, TakahiroGargin, VitaliySekiguchi, FumikoGargiulo, ErnestoSekler, IsraelGariboldi, Marzia BrunaSekulovski, NikolaGarofalo, CinziaSeledtsov, Victor IvanovichGarrido-Martín, Eva MaríaSellberg, FelixGarriga, PereSellin, LorenzGarvalov, Boyan K.Selva, Kevin J.Gary, StephensSelvam, KandasamyGarza-Rodríguez, María De LourdesSemenova, Irina V.Garziano, MicaelaSemrau, StefanGascon, EduardoSen, RwikGaskill, PeterSenbonmatsu, TakaakiGasnier, BrunoSengodan, SatheeshGáspári, ZoltánSengupta, KaushikGasparian, AlexanderSengupta, ShuvasreeGasparotto Junior, ArquimedesSenthil, RethinamGasparotto, DanielaSeo, Je HyunGašparović, Ana ČipakSeo, Ji HaeGastaldello, StefanoSeo, WooseokGasull, XavierSeo, Young-kyoGatesy, John E.Seow, Chun Y.Gatt, MosheSepulveda, RosarioGattei, ValterSerban, Andreea-irenGatti, LauraSerefko, AnnaGatti, PietroSergey, KasparovGatti, StefanoSergi, Consolato M.Gatto, Emilia M.Serova, OxanaGaudio, EugenioSerra, RaffaeleGautier, MathieuSerrano, MaríaGautier-Stein, AmandineSerrano, Maria LuisaGautvik, KaareSerres, AitorGavazzo, PaolaSert, YusufGavert, NancySertic, SarahGaviraghi, MarcoSertorio, MathieuGawel, KingaServi, Stefano DeGawlinska, KingaSetaluri, VijaysaradhiGayatri, SitaramSeto, ShigekiGazdhar, AmiqSeubwai, WunchanaGazzafi, RodrigoSevastianov, Viktor I.Ge, XiaodongSevcikova, SabinaGe, XueliangSever, BelginGe, YubinSéverin, SoniaGee, Christine E.Sevindik, MustafaGeiger, Jonathan D.Sewduth, Raj NayanGekle, StephanSeynhaeve, Ann L.Geldenhuys, WernerShadab, MohammadGelli, AngieShah, Jahan MohammadGeloso, MariaShah, Mujahid AliGembardt, FlorianShah, RuchiGena, PatriziaShah, SakhawatGeng, DaoyingShahbazi, Marta N.Geng, FengShahid, AyazGennai, AlessandroShahin, SabaGenovese, FedericaShahriyari, LeiliGenovese, IlariaShahzad, AnwarGenta, Fernando ArielShaik, AfzalGentileschi, PaoloShaked, Natan T.Gentry, MatthewShaker, AnisaGeoffray, Marie MaudeShakya, AnishaGeorge, AmanShalaby, SamerGeorgel, PhilippeShamsaeI, BehrouzGeorgiou, Christos D.Shan, SamanthaGeorgiou, IoannisShan, Samantha S. W.Geraci, FabianaShan, ShengshuaiGerasim, V. KrivovichevShan, YiGerasimovskaya, Evgenia V.Shankar, VijayalakshmiGerbaud, ÉdouardShanmugam, GobinathGergely, LajosShannon, ThomasGerhardt, ChristophShao, WeiGericke, AdrianShao, YimingGerisch, GüntherShaposhnikov, MikhailGerke, VolkerSharakhov, IgorGerlitz, GabiShariatpanahi, Seyed PeymanGermana, AntoninoSharif, JafarGermanidis, GeorgiosSharif, NajamaGerner, ChristopherSharma, AartiGernone, AngelaSharma, AbhishekGeronikolou, StylianiSharma, AjayGesmundo, IacopoSharma, AkankshaGeso, MoshiSharma, AtashiGessi, StefaniaSharma, Charu LataGetz, GodfreySharma, DeepakGevertz, JanaSharma, IshaGeyer, Pamela K.Sharma, Jai PrakashGhantous, YasmineSharma, NidhiGharagozloo, MarjanSharma, RahulGharari, ZahraSharma, Ravi KumarGhare, Smita S.Sharma, RishiGharipour, MojganSharma, RitinGhavami, SaeidSharma, RohitGheibi, SevdaSharma, ShilpyGhelli, Anna MariaSharma, ShwetaGheuca-Solovastru, LauraSharma, SuashGhezzi, DanieleSharma, VikasGhimire, Bimal KumarSharon, DeniseGhiroldi, AndreaShashikanth, NiteshGhita, MihaelaShaun, Okuda KazuhideGhogare, RishikeshShayakhmetov, Dmitry M.Ghosh, AmalenduShcherbakov, DmitriyGhosh, ArnabShea, Thomas B.Ghosh, DipanjanSheats, KatieGhosh, PriyankaShefler, IritGhosh, RajeshwarySheha, HosamGhosh, SharmilaSheikh, AbdullahGhosh, ShatadalShekhawat, Gyan SinghGhosh, SougataShelake, Rahul MahadevGhosh, SourishShemirani, Amir HoushangGhosh, SujoyShen, Che-Kun JamesGhoshdastider, UmeshShen, HaihongGhoubay, DjidaShen, LeGhouri, YezazShen, ShichenGi, GuangchenShen, Xing-jiaGiacomelli, LucaSheng, KuanweiGiacomello, EmilianaSheng, YonghuaGiannakouros, ThomasSheng, YueGiannikou, KrinioShentu, XingchaoGiannoudis, AthinaShepherd, JulianGiansanti, AndreaSherif, Zaki A.Giansanti, Maria GraziaSherin, PetrGiantsis, IoannisSherman, MichaelGibellini, LaraSherwani, Mohammad AsifGibney, NoelSheteiwy, MohamedGibson, PeterSheval, EugeneGidaro, AntonioShevchuk, OlgaGigoux, VéroniqueShi, AoGil, Cristiane DamasShi, DanGil, JeovanisShi, HaiboGilardini, Montani MariaShi, SamuelGill, DiamondShi, WeiyunGill, KatherineShi, ZhiGill, Upkar S.Shiba, KogikuGilloteaux, JacquesShibamoto, YutaGil-Sanz, CristinaShibata, HidekiGim, Jeong-AnShibata, YasushiGimple, Ryan C.Shibuya, HiroshiGioacchini, GiorgiaShidlovskii, YuliiGiorgi, Federico ManuelShigeyasu, KunitoshiGiovannoni, RobertoShih, Hsiu-mingGiovannotti, MassimoShih, Yu-RuGiovarelli, MatteoShiina, MarisaGiovarelli, MirellaShima, YoshihitoGirada, ShravanShimada, Briana K.Girard, LourdesShimazoe, TakaoGirard, MarcShimizu, KaorukoGirard, Pierre-MarieShimizu, YusukeGiraud, Marie NoëlleShimojo, MasahitoGiraud-Billoud, MaximilianoShimoni, SaraGirault, AlbanShimura, TsutomuGirdauskas, EvaldasShimura, YujiGirdhar, KhyatiShin, DonghunGirotto, GiorgiaShin, KichulGisel, AndreasShin, Meong CheolGissen, PaulShin, Teo JeonGiurisato, EmanueleShinde, PramodGiuseppe, MusumeciShirai, TsuyoshiGiusti, IlariaShirasuna, KoumeiGizak, AgnieszkaShirato, KenGkika, DimitraShirazi, SajjadGkretsi, VasilikiShiro, SuetsuguGladysheva, Inna P.Shirokikh, Nikolay E.Glazyrin, YuryShishkoff, NinaGluhovschi, CristinaShishmanova-Doseva, MichaelaGlumac, SandroShivanna, VinayGnanapavan, SharmileeShivapurkar, NarayanGnanasekaran, PrabuShizu, RyotaGöbel, HartmutShmakova, AnnaGodfraind, CatherineShmarakov, IgorGoel, Hira LalShnyder, SteveGoel, PragyaShokat, SajidGoericke-Pesch, SandraSholkamy, AmanyGoette, AndreasShortland, PeterGohy, SophieShoshan-Barmatz, VardaGöker, ErdemShramova, Elena IvanovnaGoker-Alpan, OzlemShrestha, HridayaGolab, AdamShrestha, RupendraGoldfinger, Lawrence E.Shrinivas, KrishnaGoldman, James E.Shroff, NehaGoldring, Mary B.Shtil, Alexander A.Golla, Upendar RaoShtutman, MichaelGolovleva, IrinaShu, XiaodongGomes, Ana RitaShui, WenqingGomes, Giovanni FreitasShutt, TimeGomes, Joao R.Shvartz, Vladimir A.Gómez, AnaShyr, Chih RongGomez, DianaSiano, GiacomoGomez, Gomez EnriqueSibson, David RossGómez, IvánSiddiqui, Shoib SarwarGoḿez, RáulSideris, NikolaosGomez-Lira, MacarenaSidhu, IshnoorGómez-Salinero, Jesús MaríaSiebenhaar, FrankGomora, Juan CarlosSiebzehnrubl, FlorianGomzikova, MarinaSiemiątkowska, AnnaGonatopoulos-Pournat, ThomasSieniawska, ElwiraGonçalves, CatarinaSiennicka, AldonaGonçalves, FelixSieprawska, ApoloniaGonçalves, JoanaSierecki, EmmaGonçalves, JoãoSieving, Paul A.Gonfloni, StefaniaSigalotti, LucaGong, ChangSiggins, Matthew K.Gong, GuohuaSignorello, Maria GraziaGong, MinghaoSignoriello, GiuseppeGong, PengtaoSikirzhytski, VitaliGongora-Rubio, Mario RicardoSikora, JoannaGontcharov, Andrey A.Silachev, Denis N.Gonzalez, AntonioSilke, JohnGonzález, Maglio DanielSillence, DanGonzález, María EugeniaSillitoe, RoyGonzalez, Mariscal IsabelSills, E. ScottGonzalez, MichaelSilnikov, Vladimir N.González, Sonia BenítezSilva, Célia C. G.González-Álvarez, MartaSilva, João CarlosGonzalez-Aseguinolaza, GloriaSilva-Cázares, Macrina B.Gonzalez-Benito, Maria ElenaSilva-Vignato, BárbaraGonzález-Espinosa, ClaudiaSilveira-Nunes, GabrielaGonzález-Fernández, RebecaSilvestre, Francisco JavierGonzález-González, RogelioSima, LiviaGonzalez-Mariscal, LorenzaSimmen, Frank A.Gonzalez-Menendez, PedroSimmen, ThomasGonzález-Sancho, José ManuelSimon, ItamarGonzález-Sarmiento, RogelioSimon, LizGoossens, GijsSimon, LukeGopal, JudySimon, ThomasGopal, RadhaSimone, GiuseppeGóralska, KatarzynaSimonetti, ManuelaGorantla, SanthiSimpson, FionaGorbacheva, LiubovSimu, Shakina YesminGorbe, AnikõSimula, LucaGorchakov, Andrey A.Simunovic, FilipGordeeva, OlgaSinclair, DavidGordon, Grant R.Šindelka, RadekGorelkin, PeterSinescu, Crina JulietaGorgey, Ashraf S.Singer, Bernhard B.Gorgisen, GokhanSinger, Eric A.Gorgulu, KivancSingh Tuli, HardeepGorio, AlfredoSingh, AmitGöritz, ChristianSingh, AmritGorman, Peter H.Singh, BalrajGorrell, MarkSingh, BandanaGosling, MartinSingh, BrijeshGospe, Sidney M.Singh, Deepak KumarGoto, KatsumasaSingh, GurinderGottikh, MarinaSingh, HarpalGoud, BrunoSingh, KarmveerGould, RobertSingh, KuljitGoulet, Dennis R.Singh, LataGourdie, Robert G.Singh, MahendraGouspillou, GillesSingh, Mithalesh KumarGovernini, LauraSingh, NikhleshGovindaraju, T.Singh, PradeepGovoni, AlessandraSingh, Prashant KumarGowda, Naveen KumarSingh, RaghunathGozal, EvelyneSingh, RajeevGozdz, AgataSingh, RajeshGożdzik, KatarzynaSingh, RakeshGrabbe, StephanSingh, Ratnesh KumarGrabowska, Anna M.Singh, SandeepGrabowska-Kowalik, IwonaSingh, Shashi ShekharGraeber, SimonSingh, Sumit RandhirGraef, MartinSingh, TanveerGramolelli, SilviaSingh, VikashGrand, RogerSinha, AbhilashaGranica, SebastianSinha, DebottamGranito, AlessandroSinha, SatrajitGrant, MichaelSinigaglia, AlessandroGrasemann, CorinnaSinkovic, AndrejaGrässel, SusanneSioofy-Khojine, AmirbabakGrassilli, EmanuelaSioud, MouldyGrativol, CliciaSirago, GiuseppeGrau, JamesSiraj, AbdulGrau, MarijkeSirbu, OvidiuGraves, Steven M.Sirisi, SoniaGravett, Michael G.Sirohi, KapilGray, MichaelSiska, PeterGrazi, Gian LucaSitarek, PrzemysławGrbic, VojislavaSitia, LeopoldoGreabu, MariaSitkiewicz, DariuszGreen, MichaelSiuda, JoannaGreen, NicolaSiva Kumar, SolletiGreiner, JohannesSivamaruthi, Bhagavathi SundaramGreinert, RüdigerSiwach, PriyankaGreither, ThomasSiwko, StefanGrewal, Ajmer SinghSjögren, MarieGrewal, Raji P.Skarzynski, DariuszGrewal, ThomasSkatchkov, SergueiGribova, VarvaraSkiba, Dominik S.Grieco, TeresaSklan, EllaGriesser, MichaelaSklaviadis, TheodorosGriffith, MaySkok, MarynaGriffiths, WilliamSkokou, MariaGrigaravičius, PauliusSkottman, HeliGrigorov, IlijanaSkoufias, Dimitrios A.Grillo, ElisabettaSkoufos, GiorgosGrimaldi, GiovannaSkoulakis, Efthimios M. C.Grimm, Marcus O. W.Skov, SørenGrimsrud, PaulSkrabana, RostislavGriñán-Ferré, ChristianSkriver, KarenGriñán-Lisón, CarmenSkrypnik, DamianGrinberg-Bleyer, YenkelSkrypnik, LiubovGroschner, KlausSkrzypek, KlaudiaGross, Julia ChristinaSkrzyszowska, MariaGross, OliverSlade, DeaGroßhans, JörgSlade, Jill M.Grote, KarstenSlade, NedaGrötzinger, CarstenSlama, PetrGrubczak, KamilSlavik, LudekGruber, FlorianSlezak-Prochazka, IzabellaGruber–Bzura, Beata M.Slominski, AndrzejGrubić Kezele, TanjaSlomka, ArturGrunig, GabrieleSłomski, RyszardGrünweller, ArnoldSlotkin, R. KeithGrusch, MichaelSłowikowska-Hilczer, JolantaGruss, OliverSluzalska, Katarzyna D.Grützkau, AndreasSmetana, KarelGryganskyi, Andrii P.Šmída, MichalGrzegorzewska, AgnieszkaSmirnov, AlexandreGrzesiak, JakubSmit, LindaGrześk, GrzegorzSmith, ColinGrzywa, TomaszSmith, GeorgeGrzywacz, KamillaSmith, Jill P.Gu, HuanSmith, Kevin T.Gu, JianguoSmith, Lucas R.Gu, QinshengSmith, Micholas DeanGu, WenyiSmith, StevenGu, ZhenglinSmits, AnjaGuaita, AntonioSmolarczyk, RomanGualdi, GiulioSmolińska, NinaGualtieri, RobertoSmyth, JamesGualtiero, GualtieroSnider, AshleyGuan, DailuSnow, Andrew L.Guardia, CarlosSnow, StephanieGuarnieri, SimoneSnytnikova, Olga A.Gudey, Shyam KumarSoares, FraserGuenancia, CharlesSoares, HelenaGueron, GeraldineSoares, Martins TâniaGuerout, NicolasSobacchi, CristinaGuerrera, Ida ChiaraSobhani, IradjGuerrero, Ángel L.Sobinoff, AlexanderGuest, Deborah JaneSoboloff, JonathanGuevara, Garcia AngelSobrido-Cameán, DanielGuevara, María GabrielaSochocka, MartaGugerell, AlfredSodora, Donald L.Gugliandolo, EnricoSohrabi, Kashani AhmadGuha, MayukhSokol, ElizabethGuidi, SandraSokolov, AlexeyGuieu, RégisSokolovskaya, AlisaGuigas, BrunoSokolowska-Wojdylo, MalgorzataGuihot, AmélieSolanke, Amolkumar U.Guillen-Muñoz, Juan M.Solano, FranciscoGuillén-Ponce, CarmenSolary, EricGuinamard, RomainSolé, Cañadas CarlaGujarati, Nehaben A.Sol-Foulon, NathalieGukovsky, I.Soliman, Elshakhs NeveenGulbis, BéatriceSoliven, Betty C.Gulic, TamaraSolomonia, RevazGullo, GiuseppeSolon, JérômeGulshan, KailashSolopov, PavelGulya, K.Solovchenko, AlexeiGumpert, AnnaSomanath, PayaningalGunning, William T.Somma, Maria PatriziaGuo, AnyuanSommar, PehrGuo, ChunSonah, HumiraGuo, LiSonar, Sandip AshokGuo, MingleiSong, AnyingGuo, QingxiangSong, ChunhuaGuo, QiongSong, GuanbinGuo, RuiSong, LinGuo, RuomiSong, LinshengGuo, ShichengSong, Na-YoungGuo, TheresaSong, WenxinGuo, WeiSong, XiaojinGuo, WentingSong, ZhiyinGuo, YahuiSoni, AashishGuo, YixuanSoni, NeerajGuo, YouzhongSonnino, SandroGupta, AnushkaSonntag, Kai-ChristianGupta, ArnabSonoda, Koh-HeiGupta, MaheshSontheimer, HaraldGupta, SeemaSopel, NinaGupta, ShantanuSophie, JoanisseGupta, SharadSoplinska, AleksandraGupta, YashSopper, SieghartGureev, VladimirSorci, GuglielmoGurevich, Eugenia V.Sordo-Bahamonde, ChristianGurevich, Vsevolod V.Soreq, HermonaGurke, RobertSorge, MatteoGuru, Sameer AhmadSoria, BernatGurumurthy, AishwaryaSoria-Gómez, EdgarGurung, SonamSoriano-Ursúa, Marvin AntonioGururani, Mayank AnandSorice, GianpioGurusamy, NarasimmanSorice, MaurizioGuryanova, Svetlana V.Sorokin, Vitaly A.Gurzov, EstebanSorokina, OksanaGusar, VladislavaSorrentino, RaffaellaGusev, EvgeniiSosic, IzidorGustavsson, MartinSossey-Alaoui, KhalidGutiérrez, CrisantoSotiropoulou, Christina D.Gutiérrez, LauraSoto, Guzman JesusGutiérrez, María LauraSoto-Rojas, Luis O.Gutiérrez, Uzquiza ÁlvaroSoubannier, VincentGutierrez-Aguilar, ManuelSoucek, LauraGutiérrez-Cárdenas, JuanSoukup, OndrejGutiérrez-Merino, CarlosSoundharrajan, IlavenilGuyer, RichardSousa, ÂngelaGuzmán, Ruiz RocíoSousa, MárioGuzmán-Flores, Juan ManuelSouza, MeniraGyurcsik, BelaSowislok, Andrea AnnaHa, DatSoylu-Kucharz, RanaHa, EunyoungŠpaková, IvanaHa, JihwanSpampinato, Santi M.Haanes, KristianSpanic, ValentinaHaastert-Talini, KirstenSparagna, Genevieve C.Habib, AidaSparrer, Konstantin M. J.Habib, Amyn A.Specchia, GiorginaHabibi, EhsanSpector, Stephen A.Hachlafi, Naoufal ElSpencer, Gaynor E.Hacia, Joseph G.Spencer, Juliet V.Haddad, MansourSpetz, Anna-LenaHadziselimovic, FarukSpinelli, ClaudioHafeez, RahilaSpira, Micha E.Hägerling, RenéSposi, Nadia MariaHagey, DanielSpritzer, Mark D.Hahne, JensSpurgeon, MeganHahne, MichaelSpurney, Robert F.Haig, DavidSpyratou, EllasHajiahmadi, ZahraSquecco, RobertaHajra, AdrijaSreekumar, Parameswaran G.Hakuno, FumihikoSridhar, AdithyaHalaban, RuthSrinivas, MidturuHalder, Sebok KumarSrinivasan, MukundHale, TabenSripathi, Srinivas R.Halim, Sobia AhsanSripetchwandee, JirapasHall, DuaneSrivastava, AkritiHall, GentzonSrivastava, AshishHallett, MauriceSrivastava, Madhu B.Hallstrand, Teal S.Srivastava, NityanandHam, JiyeonStaab-Weijnitz, ClaudiaHamamoto, RyujiStaal, Frank J. T.Hamczyk, Magda R.Stacchiotti, AlessandraHamdani, NazhaStachowicz, KatarzynaHamilton, NoémieStacker, StevenHammerschmidt, MatthiasStadlbauer-Köllner, VanessaHammond, Rosemarie W.Stadler, MarcHamond, CamilaStadler, RudolfHamoud, ShadiStafuzza, Nedenia BonvinoHan, ChendongStagni, VenturinaHan, DongdongStaiano, LeopoldoHan, EugeneStallone, GiovanniHan, HanŠtampar, MartinaHan, Ho-SeongStančiaková, LuciaHan, Hyung-SooStanciu, Gabriela DumitritaHan, Ik-HwanStănculeanu, Dana LuciaHan, JingyanStanescu-Spinu, Iulia-IoanaHan, JinmingStange, KatjaHan, PingpingStanislawska-Sachady, AnnaHan, RenzhiStaniszewska, MagdalenaHan, ZhenboStanke, FraukeHan, ZongchaoSt-Arnaud, ReneHanani, MenachemStasiolek, MariuszHanash, Samir M.Stathopoulos, ConstantinosHancock, Wayne W.Stathopoulou, KonstantinaHanel, WalterStaudacher, ErikaHanifeh, MohsenStaudt, David W.Hannun, Yusuf A.Staveley, Brian E.Hansen, Hinrich P.Stawski, RobertHansen, TorbenStayner, CherieHansikova, HanaStefan, GabrielHanson, Aimee L.Stefanantoni, KatiaHanson, JulienStefanescu, RazvanHantke, KlausStefania, NiadaHänze, JörgStefanowicz-Hajduk, JustynaHao, ChunyiStefanska, BarbaraHao, QinyuStein, DonaldHao, XiaSteinecker-Frohnwieser, BibianeHaoqiang, YuSteiner, GünterHappel, ChristineSteinman, LawrenceHaqqani, ArsalanStendahl, OlleHaque, MuhammadStepanov, Alexey V.Harada, AkihitoStephen, YarwoodHaragan, AlexanderStępień, TomaszHaraguchi, TokukoStępniewski, JacekHarbauer, AngelikaSterlini, BrunoHarding, Alfred T.Stern, PeterHardiville, StephanStewart, AdeleHare, Joshua M.Sticherling, MichaelHarfoot, RhodriStixova, LenkaHarmer, NicholasStoccoro, AndreaHarmsen, Martin C.Stock, WillemHarrer, AndreaStocker, ClaireHarrer, DennisStoeckl, JohannesHarris, AndrewStoiber, DagmarHarris, DavidStojak, MartaHarrison, PaulStojanović, NikolinaHarsing, Laszlo G.Stojanovich, LjudmilaHartman, Jessica H.Stoll, GautierHartner, AndreaStommel, ElijahHartwig, ThomasStorchova, ZuzanaHashemolhosseini, SaidStöß, ChristianHashimoto, AriStossi, FabioHashimoto, KenjiŠtrac, Dubravka ŠvobHashimoto, YoshihideStradal, TheresiaHashita, TadahiroStrader, Michael BradHassan, MohamedStrano, SabrinaHastings, Philip J.Strappazzon, FlavieHasumi, KeijiStrasser, RichardHata, KenjiStratton, RichardHata, MasakiStrauss, LeenaHathaway, Nate A.Strecker, Robert E.Hatipoglu, FatihStrettoi, EnricaHattori, YoritoStroud, Matthew J.Hau, PeterStrum, StephenHaudenschild, Dominik R.Študentová, HanaHaumaitre, CécileSturchio, AndreaHausch, FelixSturla, LauraHausser, AngelikaStutz, BernardoHautefeuille, MathieuSu, HuaboHawkes, DavidSu, JianbinHawkridge, Adam M.Su, JianminHawthorne, SusanSu, PingHay, EricSu, ShangHayashi, MakotoSu, SiyuanHayashi, MikioSu, YapengHayashi, YoshinoriSu, YijingHayashida, KenjiSuades, RosaHayashida, NaokiSubramaniam, SaravananHayer, ArnoldSubramanyam, RajagopalHayıroğlu, Mert İlkerSuccu, SaraHayward, Lawrence J.Suda, KenichiHazafa, AbuSuda, ToshioHe, BinSue, KyleHe, JunqiSugawara, ShigekiHe, JunxianSuh, Chang-HeeHe, PeijianSui, HaixinHe, QiushuiSukhorukov, VasilyHe, WangxiaoSuklabaidya, SujitHe, XiaochenSukumaran, Sunil KumarHe, YaodongSukumaran, VijayakumarHe, ZhigangSulek, AnnaHe, ZhiguoSuliman, Hagir B.Healey, JohnSułkowska-Ziaja, KatarzynaHeath, PaulSulová, ZdenaHebeda, Konnie M.Summers, MatthewHefferon, KathleenSun, DaHegazy, Mohamed-Elamir F.Sun, HongliuHeide, MichaelSun, JunHeidenreich, OlafSun, Jun-RenHeinbockel, ThomasSun, LingHeindl, Ludwig M.Sun, QinyuHeinrich, StefanSun, RunziHeise, Herbert MichaelSun, ShixiangHejmej, AnnaSun, Wei-HsinHelbok, RaimundSun, XiangjieHeldin, ParaskeviSun, Xiao-MingHelguero, LuisaSun, YinHellemons, Merel E.Sun, YitangHeller, JanoschSun, YuHellingwerf, Klaas J.Sun, YunfuHellman, LarsSundaram, Gopinath M.Hellmann, NadjaSundaram, Sivaraj MohanaHellmen, EvaSundararajan, RajiHellysaz, ArashSundararajan, VenkateshHelsper, C.W.Sundaresan, Nagalingam RaviHemberger, MyriamSundfeldt, KarinHemmersbach, RuthSung, Pil SooHemmings, DeniseSunnagatta, Nagaraja SunilgowdaHemphill, Wayne O.Sunny, SiniHen, RenéSunseri, FrancescoHendrix, MarySur, IlknurHenique, CaroleSur, Vishma PratapHennies, Hans ChristianSurapaneni, Krishna MohanHenry, Ryan A.Suravajhala, Prashanth N.Her, Guor MourSurguchov, AndreiHerath, Chandana B.Surve, Chinmay R.Herault, YannSussman, MarkHerb, MarcSutanto, HenryHerbath, MelindaSutherland, Ann E.Herbelet, SandrineSuvorov, Alexander NikolaevichHerbst, RuthSuvorova, Irina I.Herencia, CarmenSuzdaltseva, YuliaHerman-Antosiewicz, AnnaSuzuki, AkikoHermann, EvaSuzuki, AussieHermawan, AdamSuzuki, HideoHermouet, SylvieSuzuki, HirotadaHermsen, MarioSuzuki, KazuhitoHernandez, Herrera PaulSuzuki, KeisukeHernández, Rivera JuanSuzuki, MasatoHernandez-Alcoceba, RubenSuzuki, MikioHernández-León, AlejandroSuzuki, SusumuHernández-Pérez, SaraSuzuki, ToshikazuHernandez-Romero, DianaSuzuki-karasaki, YoshihiroHerold, ZoltanSüβ, PatrickHerrera, Antonio J.Svedruzic, ZeljkoHerrera, BlancaSveshnikova, AnastasiaHerrera, EloisaSvirshchevskaya, Elena V.Herrera, HectorSvirskis, SimonsHerrera-Marschitz, MarioSwain, Jason E.Herrera-Molina, RodrigoSwann, KarlHerrera-Rincon, CeliaSwanson-Mungerson, MichelleHerrero-Galán, ElíasŚwia̧tkiewicz, IwonaHerreros-Pomares, AlejandroSyafruddin, Saiful EffendiHersey, PeterSzabelski, JakubHesami, MohsenSzabo, GyongyiHess, BeckySzakacs, GergelyHeuer-Jungemann, AmelieSzandruk-Bender, Marta JoannaHeumann, RolfSzászi, KatalinHeurtaux, TonySzatmari, SzabolcsHeyam, HayderSzczerbal, IzabelaHeyland, AndreasSzczesny, BartoszHeymann, J. BernardSzegö, Éva M.Hibaoui, YoussefSzeliga, MonikaHibshman, Jonathan D.Szentmáry, NóraHickey, Scott E.Szepanowski, FabianHida, TokimasaSzewczuk, Myron R.Hidalgo, GuillermoSzibor, MartenHiebert, PaulSzijan, IreneHieronymus, ThomasSzilágyi, MelindaHigaki, TakayaSzostak, RomanHiguchi, AkonSzpechciński, AdamHiguchi, KyokoSzychowski, Konrad A.Hildyard, John C. W.Szydlak, RenataHilgen, GerritSzyfter, KrzysztofHilhorst, MarcTabarkiewicz, JacekHillion, SophieTabatabaeian, HosseinHiltunen, MikkoTabatabaie, FatemehHime, GaryTachibanaki, ShujiHimeda, Charis L.Tada, YuichiHimoto, TakashiTaddei, Maria LetiziaHinck, LindsayTagawa, TakanobuHinden, LiadTago, KenjiHinds, Terry D.Taguchi, ToruHintschich, Constantin A.Taha, SalmaHiram, RoddyTaha, ZaidHirano, KatsuyaTahmasbpour Marzouni, EisaHirasawa, NoriyasuTaira, ShuHirata, YokoTajiri, KazutoHirata, YusukeTakabayashi, ShujiHirayama, KouichiTakahara, TerunaoHiroki, Carlos H.Takahashi, ChiakiHirota, KiichiTakahashi, HidehisaHirotsu, TakaakiTakahashi, MasayukiHirozane, ToruTakai, YoshikiHirsch, EmilioTakakubo, YuyaHjelmeland, AnitaTakamatsu, ShinjiHjelmen, Carl E.Takano, KatsuraHlavaty, JurajTakasawa, ShinHnilička, FrantišekTakashima, ShotaHo, Cheng-MawTakatani-Nakase, TomokaHo, Ivy A.Takegahara, NorikoHo, Shu LeongTakeuchi, HirokiHo, Wan YongTalagas, MatthieuHobbs, Gabriela SorianoTalbert, ErinHochstrasser, MarkTalia, MariannaHocking, Jennifer C.Talianidis, IannisHodgetts, StuartTallaksen, Chantal Me E.Hodgman, Charlie T.Tallquist, MichelleHodkovicová, NikolaTalmon, MariaHoerning, AndréTalora, ClaudioHoetzenecker, KonradTalukdar, SarmisthaHoffmann, Lucia VieiraTam, Roger Y.Hofman, PaulTamagno, ElenaHohendanner, FelixTamamoto-Mochizuki, ChieHohenegger, MartinTamaskovics, BálintHohmann, TimTamkovich, SvetlanaHohwieler, MeikeTammaro, AlessandraHojsgaard, DiegoTamura, YasuakiHokugo, AkishigeTan, Kai SenHolada, KarelTan, Lu PingHollemann, ThomasTan, Meng HowHollenhorst, Monika I.Tan, SuiyiHolliday, L. ShannonTanabe, ShihoriHollingsworth, T. J.Tanaka, HiroshiHolly, JeffTanaka, Hiroyoshi Y.Holmager, PernilleTanaka, MasaruHolnthoner, WolfgangTanaka, MiyakoHolt, Scott M.Tanaka, SatoshiHolub, KatarzynaTanaka, SusumuHoma, Fred L.Tanaka, TeruyoshiHomma, TakujiroTanaka, ToshiyaHonari, ArvinTanaka, YoshiakiHong, Chien-TaiTanaka, YoshimasaHong, HuilingTanavde, VivekHong, Hyun SookTang, BinHong, Jeong HeeTang, DamuHong, Jiann-RueyTang, WenwenHong, Sung NohTang, YameiHongwei, GuoTang, YuHoppler, StefanTaniguchi, HiroakiHoque, MehboobTaniguchi, KoheiHordijk, PeterTaniguchi, NaoyukiHore, Peter J.Taniyama, YoshiakiHorikawa, IzumiTanner, N. KyleHorin, PetrTantilipikorn, PongsakornHorng, Chi-TingTantos, ÁgnesHornos Carneiro, Maria FernandaTanwar, JyotiHorowitz, Robert A.Tao, GeHorowitz, ScottTao, JianliHorsman, Michael RobertTao, KaiHorstmann, MartinTao-Cheng, Jung-HwaHorváth, GabriellaTaouis, MohammedHoshiba, TakashiTapia, Alejandro A.Hosoya, MakotoTappia, Paramjit S.Hossain, Md. SanowerTaranta, AnnaHossein, GhamartajTarantino, GiovanniHosseini, Fatemeh S.Tarashi, SamiraHossein-Zadeh, Navid GhaviTarasiuk, AleksandraHostiuc, MihaelaTarasov, AndreiHosui, AtsushiTarasova, Olga S.Hosur, VishnuTarique, AbdullahHou, YingchunTarlinton, RachaelHousset, ChantalTarnacka, BeataHowe, AnitaTarnowski, MaciejHowlett, AllynTarquini, GiuliaHoyer, J. SteenTartara, Fulvio AlbertoHoyne, Gerard F.Tata, Despina A.Hribal, MartaTatarova, ZuzanaHritcu, LucianTatullo, MarcoHrnčić, DraganTaub, MaryHsia, KuochiangTaube, JosephHsiao, Hui-HuaTaurin, SebastienHsieh, Ming-ChingTautenhahn, Hans-MichaelHsieh, Tusty-JuanTavakoli, MehdiHsieh, Yu-LinTavares, Ivan MaynartHsin, I-LunTavazzi, BarbaraHsiu, HsinTaverna, SimonaHsu, Chih-HsinTawa, MasashiHsu, Chih-pingTaxiarchi, ChrysanthiHsu, Chin YuanTaye, NandarajHsu, Chung Y.Tayebati, Seyed KhosrowHsu, KateTayebi, Hend M. ElHu, CaihongTaylor, Andrew W.Hu, ChengTaylor-Papadimitriou, JoyceHu, ChenghuTchetina, ElenaHu, DongjianTchoghandjian, AurélieHu, HenglongTebar, FrancescHu, KebinTebyaniyan, HamidHu, MinTee, Shiau FoonHu, NaTeixeira, GraciosaHu, RongTelang, NitinHu, TaoboTelbisz, ÁgnesHu, Wen-YangTelek, AndreaHu, YoujiaTéllez-Valencia, AlfredoHu, YuanTellier, MichaelHu, YuntaoTemiz, Selami AykutHu, ZhiqiangTeng, LipingHuai, Chong LorTeng, WeiHuang, Andrew Chih WeiTeng, Zhao-QianHuang, CanhuaTengattini, SaraHuang, ChaoqunTeodoro, João S.Huang, Chien-ChungTeofili, LucianaHuang, Chien-YuTéot, LucHuang, Chun-JungTeran, Luis ManuelHuang, FangTerao, RyoHuang, Fu-ChenTerasmaa, AntonHuang, HaiqiuTerenin, Ilya M.Huang, Hsing I.Terentyev, Vasily V.Huang, HsiyuanTeresa, RussoHuang, HuihuiTerp, Mikkel GreenHuang, JianfengTerracciano, DanielaHuang, JijunTerrazas, Luis I.Huang, JunqingTerreni, MarcoHuang, Kevin Chih-YangTerrinoni, AlessandroHuang, Li-JunTersariol, Ivarne L.Huang, LiliTesarik, JanHuang, LulinTesfaigzi, Yohannes A.Huang, MianTesic, VesnaHuang, Ming-YiiTeske, NicoHuang, Shau-KuTessem, Jeffery S.Huang, ShengyouTestini, ChiaraHuang, Shu-PinTétreault, MartineHuang, SuiTeyssier, CatherineHuang, SulingTezcan, GülçinHuang, TaoThakare, RiteshHuang, TimothyThakur, ArchanaHuang, YangpeiweiThan, Tin A.Huang, YiminThanabalu, ThirumaranHuang, Yu-FangThangudu, SureshHuang, ZiliangThatikonda, SowjanyaHubal, Monica J.Thawani, AkankshaHübener-Schmid, JeannetteTheotokis, PaschalisHuber, ArminThiel, UweHublitz, PhilipThiriet, ChristopheHübner, MaxThomas, Biju B.Huckle, WilliamThomas, ElizabethHudmon, AndyThomas, JordanHudson, MatthewThomas, LintoHuebbers, Christian U.Thomas, Stefani N.Huerta, MiguelThome, UlrichHueso, MiguelThompson, Peter J.Huey-Shan, HungThompson, RuthHugo, LeonThor, StefanHui, XiaoyanThorens, BernardHukkanen, JanneThornton, ClaireHull, J. JoeThuillier, RaphaëlHulleman, JohnThurnher, MartinHulme, CharlotteTian, AiguoHulme, John P.Tian, HengliHumeres, ClaudioTian, LingHumrich, Jens Y.Tian, LiningHung, Jan-JongTian, WeihuaHupé, PhilippeTian, YeHur, JungukTicconi, CarloHurwitz, StephanieTicinesi, AndreaHusain, ShahidTidy, AlisonHuss, AndreTiidus, PeterHussain, AdilTikhonova, MariaHussain, MansoorTimmons, LisaHussain, Muhammad SajidTiperciuc, BrindusaHussain, NadiaTiriveedhi, VenkataswarupHussain, SabahTisi, AnnamariaHussain, TariqueTit, Delia MirelaHussein, Kamal HanyTitov, Sergei E.Huttemann, MaikTittarelli, AndrésHüttenhofer, AlexanderTitus, MarkHuwiler, AndreaTiwari, ArchanaHuxford, TomTiwari, Rahul KumarHuynh, NamTiwari, SwapnilHwang, In KooToba, HiroeHwang, JongikTodaro, Laura BeatrizHybertson, Brooks M.Todi, Sokol V.Iaccarino, CiroTodinova, SvetlaIaccarino, NunziaTodorov, SvetoslavIacobucci, IlariaTodorović, AnaIacomino, GiuseppeToegel, StefanIacono, SalvatoreToh, Han SiongIacoviello, MassimoToh, Wei SeongIbáñez-Fonseca, ArturoToh, Yi LongIbarretxe Bilbao, GaskonTokumitsu, HiroshiIbrahim, Mahrous AbdelbassetTokunaga, FuminoriIchas, FrançoisTollár, JózsefIde, KazukiTolomelli, AlessandraIdo, YasuoTolomeo, AnnamariaIdoate, MiguelTomar, DhanendraIdris, ZamzuriTomarev, Stanislav I.Ielapi, NicolaTomašič, TihomirIelciu, IrinaTomassetti, AntonellaIetta, FrancescaTomić, Sergej Z.Ietto, GiuseppeTomičić, MajaIgarashi, KazueiTominaga, MitsutoshiIglesias, AmandaTommonaro, GiuseppinaIglesias-Figueroa, Blanca F.Tomokiyo, AtsushiIglicki, MatiasTomos, IoannisIgoillo-Esteve, MarianaTomuleasa, CiprianIgotti, MariaTonali, NicoloIguchi, YoheiTong, Chun Kit BenjaminIijima, KazutoshiTong, ShupingIjaz, MuhammadTong, TaoIjichi, HideakiTong, WenxinIkebe, MitsuoTong, XiaoyongIkeda, KazuhiroTong, ZhongchunIkegame, MikaTonhajzerova, IngridIkkonen, Elena N.Ton-Hoang, BaoIkram, MuhammadTonn, Jörg C.Ilchibaeva, TatianaTooley, Christine E. SchanerIldikó, TarTopp, Kimberly S.İlgen, UfukTorban, ElenaIlias, IoannisTorices, SilviaIlies, Marc A.Torikai, YukiImai, JunTornóczky, TamásImai, YuzuruTörnquist, KidImamoto, NaokoToro, Carlos AlejandroImamura, SousukeToro, RocioImbimbo, CiroToronov, VladislavImhof, PetraTorraco, AlessandraImokawa, GenjiTorrado, EgídioImran, SaimaTorras-Ambròs, JoanIn’t, Veld SjorsTorre, VincentInaba, MayuTorrente, AngeloInagaki, YoshinoriTorres, BlancaInamdar, ShivangiTorres, JosemaInamo, JocelynTorres-Aguila, Nuria P.Inan, SaadetTorres-Vergara, PabloIndiveri, CesareTorretta, EnricaIndra, RadekTorrisi, Sebastiano AlfioIngrassia, RosariaTorti, MarinIniesta, Cuerda MaríaTortosa, AvelinaIno, KosukeTósaki, ÁrpádInoue, IkuoToscani, DeniseÍnsua, ÁngelToshio, HattoriInutsuka, AyumuTossetta, GiovanniIommelli, FrancescaTosti, ElisabettaIorio, JessicaTotey, SatishIorio, RobertoToth, Balazs IstvanIosif, PediaditakisTotiger, Tulasigeri M.Irimia, ManuelTotta, PierangelaIrini, EvnouchidouToubai, TomomiIrmgard, TegederTouil-Boukoffa, ChafiaIsachenko, VladimirTouloupakis, EleftheriosIshida, TakashiTournissac, MarineIshigaki, YasuhitoTousif, SultanIshii, SanaeTownsend, Logan K.Ishikawa, MitsuruToyoda, HirokiIshiwata-Endo, HirokoToyoda, MasashiIsidoro, CiroToyohara, TakafumiIskra, MariaToyooka, KazuhitoIslam, M. RezaulTozaki-Saitoh, HidetoshiIsmael, SaifudeenTozzi, AlessandroIsraeli, DavidTraber, KatrinaIssara, UtthaponTraboulsi, Waelİşsever, HalimTrachana, VarvaraItabaiana, Ivaldo, Jr.Trachootham, DunyapornIto, ShosukeTraiffort, ElisabethIturriaga-Vasquez, PatricioTrajković, JelenaIturrioz-Rodríguez, NereaTran, Cam HaIvanisenko, Nikita V.Tran, Hai BacIvanov, StoyanTran-Nguyen, Viet-KhoaIvanovic, ZoranTraub, FrankIvarne, TersariolTravers, Jeffrey BryantIvaska, JohannaTrayhurn, PaulIwamaru, YoshifumiTremblay, DouglasIwasaki, ShinichiTrendafilova, ElinaIwaszkiewicz-Grzes, DorotaTrendelenburg, MartenIwata, JunichiTrerotola, MarcoIwata, KoichiTresguerres, JesusIyaswamy, AshokTretter, VerenaIzquierdo-Serra, MercèTreulén, FaviánJaafar, RolaTreuner-Lange, AnkeJablonska, EwaTriarico, SilviaJablonska, KarolinaTribulova, NarcisJabůrek, MartinTricard, ThibaultJacevic, VesnaTricarico, DomenicoJackson, EdwinTricoire, LudovicJackson-Weaver, OlanTrifunovic, DanijelaJacob, Harrys Kishore CharlesTrimarco, BrunoJacobi, DavidTringali, CristinaJacobo-Albavera, LeonorTrinh, Kieu The LoanJacobo-Herrera, Nadia JudithTrionfini, PieraJaconi, Marisa E. E.Tripathi, AshutoshJadavji, NafisaTripathi, Dinesh ManiJaeger, SebastianTripathi, RakshamaniJafari, RezaTripathi, RatnakarJafri, MohsinTripodi, Sergio AntonioJagtap, AjitTripon, FlorinJahroudi, NadiaTriposkiadis, FilipposJain, AbhinavTrivedi, Arun K.Jain, AklankTroncoso, RodrigoJain, AshishTrotta, MassimoJain, Bhawik KumarTrovato, MaurizioJain, ManishTrubiani, OrianaJaishankar, DineshTrueba, Gabriel A.Jaiswal, Jyoti K.Trujillo, XóchitlJakas, AndrejaTruong, Vi-KhanhJakiela, BogdanTrusiak, MaciejJakieła, SławomirTruskey, GeorgeJakovcevski, IgorTsai, Chen-LiangJakubíková, JanaTsai, Hsiang-LinJalan-Sakrikar, NidhiTsai, Hung-PeiJambhekar, AshwiniTsai, Hung-WenJames, Kit-Hon TsoiTsai, Kun-LinJammalamadaka, UdayabhanuTsai, Kun-LingJan, Arif TasleemTsai, Li-KaiJan, AsadTsai, RongkungJan, Ming-ShiouTsai, YuhsuanJana, SoumenTsang, MichaelJanczar, SzymonTsarouhas, VasiliosJandova, JanaTseng, JenchihJaneczko, AnnaTseng, Jen-ChihJanel, NathalieTseng, TojungJanić, MiodragTseng, Yi-HsinJanković, AleksandraTsepokina, Anna V.Jankowska, ElzbietaTsikou, DanielaJankowski, MichaelTsimokha, Anna S.Janoušková, Olga ŠebestováTsingotjidou, AnastasiaJanowska, AgataTsirka, Styliani-AnnaJanowski, TomaszTsitsilonis, Ourania E.Jansen, GertTsoi, HoJantas, DanutaTsotakos, NikJantunen, EsaTsubaki, MasanobuJaquet, KorneliaTsubaki, MotonariJaremko, LukaszTsuboi, MayoJarome, TimothyTsuchida, KunihiroJarry, AnneTsuchiya, HiroyukiJärvinen, PäiviTsuda, MakotoJarza̧bek-Bielecka, GrażynaTsuji, KenjiJas, Gouri S.Tsuji, ShojiJasbi, PanizTsujioka, MasatsuneJaskiewicz, EwaTsukamoto, HayatoJat, Parmjit S.Tsukamoto, ShoJaukovic, AleksandraTsuneki, MasayukiJavadov, SabzaliTsutomu, NakazawaJaworek, JolantaTsygankov, Alexander Y.Jayabalan, AravinthkumarTsytsarev, VassiliyJayaraman, SrinidhiTuboly, EszterJayaraman, SundararajanTuğcu, MuratJayawardena, DulariTuka, BernadettJazir, Sabira MohammedTuluce, YasinJazirehi, Ali R.Tünde, TarrJean Luc, MorelTuntivanich, NalineeJeans, Alexander F.Tupikowski, KrzysztofJeffery, SteveTupler, RossellaJellinger, Kurt A.Tura-Ceide, OlgaJelski, WojciechTuraga, Ravi ChakraJembrek, Maja JazvinšćakTurato, GraziellaJenness, MarkTurczyn, RomanJeon, YubyeolTurgeman, GadiJeong, AyoungTurhan, Ali G.Jeong, DaewonTurillazzi, EmanuelaJeong, GunjaeTurino, Gerard M.Jeong, Wol-IlTurki, AminJerin, AlešTurki, TurkiJetten, AntonTurksen, KursadJevremovic, SladjanaTurner, Alice M.Ježić, MarinTurner, Neil A.Jha, Narendra NathTuroverov, Konstantin K.Jha, Rajesh KumarTurovskaya, Maria V.Jhawat, VikasTurowski, PatricJi, RenleiTurrini, EleonoraJia, LongfeiTurrini, LapoJia, NingTurska, MonikaJia, SonghuiTurski, WaldemarJian, ZhongTuytten, RobinJiang, GuochunTvrdá, EvaJiang, WeiTyagi, AbhishekJiang, WeiyuTyagi, Vinay KumarJiang, YifengTyrka, MirosławJiang, YuyanTyurin-Kuzmin, Pyotr A.Jiao, KaiTzachanis, DimitriosJide, TianTzang, Bor-ShowJimbo, MitsuruTzanoudaki, MariannaJiménez Rosado, MercedesTzeng, Chii-RueyJimenez-Acosta, FranciscoTzeng, I-ShiangJiménez-Luna, CristinaUberti, FrancescaJin, HongUcciferri, ClaudioJin, HongtaoUchida, ShizukaJin, LiUchida, TakafumiJin, WenbingUchiumi, FumiakiJin, YiUchiyama, MasateruJin, YongUd, Din AhmedJin, ZhuqiuUddin, ShahabJing, ZhaoU-Din, MueezJirsova, KaterinaUdono, HeiichiroJiu, YamingUebanso, TakashiJivanji, SwatiUeda, KaoriJo, Min GiUeda, NatsuoJoana, Sofia CorreiaUehara, YoshinariJogam, PhanikanthUeki, ShigeharuJohansen, Anne KatrineUemura, TakeshiJohn, Sinu P.Ugalde, Juan E.John, T. HancockUgel, StefanoJohnson, Lance A.Uhe, TobiasJohnson, Travis KaneUhlenhaut, Nina HenrietteJohnstone, CameronUlasov, IlyaJoles, Jaap A.Ullah, H. M.Jolly, Lachlan A.Ullah, Md AshikJolly, Mohit KumarUllah, SultanJonckheere, NicolasUller, LenaJones, Robin L.Ulusu, NurayJones, Simon W.Um, JiJones, VeronicaUmair, MuhammadJoo, MyungsooUmapathy, GaneshJordheim, Lars PetterUmek, NejcJörg, GrosshansUmemura, TsukuruJörg, WeimerUmmarino, AldoJorge, ErikaUngefroren, HendrikJorgensen, Trine N.Unudurthi, SathyadevJose, LenyUosaki, HidekiJosefsson, AndreasUpadhya, DineshJoseph, ChitraUpadhyay, ArunJoshi, Abhayraj S.Upadhyay, Sushil K.Joshi, RohitUras, Iris Z.Joshi-Navare, KasturiUrbach, AnjaJost, EdgarUrbanelli, LorenaJotic, AleksandraUrbańska, Ewa M.Joubert, BastienUrbański, ArkadiuszJoung, BoyoungUrdiciain, AlejandroJovanovic, FilipUri, AskoJozef, MravecUribe, ElenaJuárez-Flores, Diana LuzUribe, Kevin P.Juarranz, ÁngelesUrik, MilanJuarranz, YasminaUrrea-Mendoza, EnriqueJuban, GaëtanUrso, DanieleJuhlin, ChristoferUrso, LoredanaJulián, EstherUsaj, MarkoJun, SadoshimaUsui, NoriyoshiJune, Ronald K.Uziel, OritJung, SungjunVacca, MircoJungnickel, ChristianVacca, Rosa AnnaJunier, Marie-pierreVaccaro, María I.Juranek, Judyta KarolinaVaclavikova, RadkaJurek, BenjaminVadas, MathewJurič, Damijana MojcaVaddi, PrasannaJurica, JanVafiadaki, ElizabethJurkowska, RenataVaidya, Anuradha A.Jyotsna, MishraVaira, Luigi AngeloKabakov, AlexanderVaira, ValentinaKabani, MehdiVaka, RamanaKabir, M. AnaulVakhshiteh, FaezehKabrah, SaeedValadez-Graham, VivianaKaca, WiesławValdez-Jasso, DanielaKachamakova-Trojanow, NeliVale, NunoKaczmarek, AgataValente, Fabrício LucianiKaczyńska, KatarzynaValenti, DanielaKaddoumi, AmalValenti, Maria TeresaKaestner, LarsValenzuela, RodrigoKaether, ChristophValerio Branca, Jacopo JunioKahali, BratatiValero, Pacheco NuribanKaida, DaisukeValero, YulemaKaissi, AliVallamkondu, JayalakshmiKaji, KosukeVallés, Ana SofiaKakabadse, DimitriValletta, RosaKakei, ShinjiValojerdi, Mojtaba RezazadehKakinoki, SachiroValor, Luis M.Kakinuma, YoshihikoValverde, AnthonyKalal, Bhuvanesh SukhlalVan Berlo, JopKalathil, SureshVan Dam, Anne-MarieKaliňáková, BarboraVan der Weyden, LouiseKaliszewska, MagdalenaVan der Zeijst, B. A. M.Kalita, JatinVan Grunsven, Leo A.Kalogiannidis, Ioannis A.Van Ha, ChienKalra, RashiVan Tilbeurgh, HermanKalwar, QudratullahVan, De LindeKamal Abdel-Aziz, AmalVan, De LooKametaka, SatoshiVan, Den AkkerKaminska, DorotaVan, Der DoesKaminska, JoannaVan, Guilder GaryKaminski, RafalVan, Hamersvelt HenkKamiński, TadeuszVan, Hateren AndyKaminski, Tomasz W.Van, Hoek BartKamisah, YusofVan, Loon JackKamitani, ShigekiVanacore, NicolaKamolz, Lars P.Vancurova, IvanaKampen, Kim RosalieVandermoere, FranckKanagy, Nancy L.VanderVeen, BrandonKanakis, IoannisVan-Dijk, JulietteKanashiro, MiltonVanDuijn, Martijn M.Kanaujiya, JitendraVanneste, SteffenKanczler, Janos M.Vannini, CandidaKanda, TatsuoVannini, NicolaKandeel, MahmoudVannucci, SusanKandimalla, RaghuramVanoni, MarcoKanekiyo, TakahisaVaranda, CarlaKang, DongchulVarela-Ramirez, ArmandoKang, Ellen H.Varga, LukasKang, JianmingVarga, ZoltánKang, Kyung PyoVargas-Rodriguez, OscarKang, Sung UngVarkouhi, Amir KhashayarKang, TaebongVarricchio, EttoreKang, Yang JaeVarzideh, FahimehKania, Stephen A.Vas, ViragKankuri, EskoVascotto, CarloKannan, AnbarasuVasić, Tanja P.Kannan, NagarajanVasileva, LiliyaKanneganti, Thirumala-DeviVasili, EftychiaKanninen, KatjaVaslin, Maite F. S.Kano, YutakaVasquez, VelmariniKansakar, UrnaVassallo, NevilleKansal, VikashVassetzky, Yegor S.Kanse, Sandip M.Vassilakos, NikonKantidakis, TheoVassilev, DobrinKantorow, MarcVassilopoulou, EmiliaKanzaki, YumikoVaughan, MelKao, Shao-HsuanVaz, Sandra H.Kao, Shung-TeVázquez Cuevas, Francisco GabrielKaoud, Tamer S.Vazquez-Roque, Ruben A.Kaplan, AlanVeenhoff, LiesbethKar, AnirbanVeerappan, ArulKar, PremashisVega, Francisco M.Karachunski, PeterVelazquez-Salinas, LauroKaragiannakis, DimitriosVelázquez-Sánchez, ClaraKaragiannis, SophiaVelickovic, KsenijaKarakaidos, PanagiotisVelikova, TsvetelinaKarakesisoglou, IakowosVelleuer, EunikeKaramanos, YannisVelusamy, PremaKarampela, IreneVenesio, TizianaKarch, FrançoisVenihaki, MariaKardassis, DimitrisVenkatesan, Jagadeesh K.Kareva, IrinaVentura, NatasciaKargozar, SaeidVenugopal, ChitraKarimi, KhalilVercellin, Alice VerticchioKarimi, SoheilVerderio, ClaudiaKariminik, AshrafVergallo, AndreaKarisola, PiiaVergani, LauraKariya, YoshinobuVergara, DanieleKarkowska-Kuleta, JustynaVergara, M. NataliaKarl, MatthiasVerges, MarcelKarlsen, Bård OveVerhoeven, AdrieKarlsson, OskarVerma, AkankshaKarn, Santosh Kr.Verma, Akhilesh K.Károly, Kristóf EndreVerma, Praveen C.Karousou, EvgeniaVerma, Shiv PrakashKarpiński, StanisławVermassen, TijlKarsdal, MortenVermeulen, NathalieKarstedt, Silvia VonVerrecchia, FranckKarthika, ChenmalaVerrier, SophieKarthikeyan, AdhimoolamVertii, AnastassiiaKarwowski, Boleslaw T.Vesa, Stefan CristianKasakovski, DimitriVesna, CoricKasam, RajeshVetere, AmedeoKasem, KulkeawVettorazzi, SabineKashanchi, FatahVezzoni, PaoloKashfi, KhosrowViana, Vívian EbelingKashiwabara, ShinichiVicario, MartaKashiwagi, ShinichiroVicens, QuentinKashtanova, DariaVicens-Zygmunt, VanessaKashyap, Vivek K.Vicente-Manzanares, MiguelKasper, CorneliaVidal, MiguelKasprzak, AldonaVidal, PiaKastaniotis, AlexanderVideira, MafaldaKastner, CarolinVieira, AlexandreKasture, Ameya SanjayVieira, FredericoKaszaki, JozsefVieira, JorgeKasztelan-Szczerbińska, BeataViela, FelipeKata, DianaViengchareun, SayKataoka, NaoyukiViennet, ThibaultKatare, Deepshikha PandeVietor, IljaKatari, VenkateshVieyra, AdalbertoKataru, Raghu P.Viggiano, EmanuelaKatayama, TakayukiVignando, MiriamKathiriya, Jaymin J.Vijayakurup, VinodKatona, GergelyVikal, YogeshKatsaraki, KaterinaVikram, AjitKatsinelos, TaxiarchisVila, MiquelKatt, MoriahVilarrasa, NuriaKaul, MarcusVilas-Boas, VâniaKaur, GaganpreetVilla, Luisa LinaKaur, KulwinderVillano, InesKaur, SavneetVillanueva-Romero, RaúlKaur, SukhbirVillarroel-Espíndola, FranzKaushik, AbhinavVillegas, VictoriaKaushik, PrashantVilmantė, BorutaitėKausik, DattaVincentini, OlimpiaKauskot, AlexandreVincenzi, FabrizioKavianpour, MariaVincenzo, RocchettiKawaguchi, NanakoVindigni, VincenzoKawale, AjinkyaVinggaard, Anne MarieKawamoto, AtsuhikoVingolo, Enzo MariaKawasaki, TakeshiVioleta, StojanovskaKawashima, NobuyukiVirdi, KamaldeepKawauchi, HideyukiVirolle, ThierryKay-Dietrich, WagnerVirtuoso, AssuntaKazak, LawrenceViscomi, MariaKazanis, IliasVisentin, SilviaKazemian, NeginVisner, GaryKazim, NoorVisser, FrankKe, Po-YuanVisser, LydiaKeevil, Brian G.Viswanathan, Vibhu KrishnanKelleher, AndrewVitale, ChiaraKeller, Jonathan R.Vitali, RobertaKellett, Katherine A. B.Vitkova, VictoriaKelley, ThomasVitorino, RuiKemper, BjörnVittori, MilošKeniry, AndrewVittorio, GebbiaKeniry, MeganVivar, CarmenKerr, Ian DerekVlachodimitropoulos, DimitriosKesavan, RushendhiranVlachonasios, Konstantinos E.Kesh, KousikVladimirovna, Roshchina VictoriaKeshari, SunitaVlashi, ErinaKeshel, Saeed HeidariVleminckx, KrisKęsik-Brodacka, MałgorzataVliagoftis, HarissiosKessler, EfratVliet, Kim VanKeszenman, Deborah J.Vliora, MariaKettritz, RalphVlkova, BarboraKettunen, PetronellaVo, GiauKhachigian, LevonVogt, SebastianKhajanchi, SubhasVoican, CosminKhalil, Magdy M.Voidarou, ChrysaKhalil, Mahmoud I.Völker, JohannesKhan, AdnanVölkl, SimonKhan, AmjadVolpi, ClaudiaKhan, ArifVolpicelli, FlorianaKhan, HaroonVolynsky, PavelKhan, Masood AlamVon Eyben, Finn EdlerKhan, Meraj AlamVon Gall, CharlotteKhan, MohsinVon Kobbe, CayetanoKhan, MuhammadVon, Hundelshausen PhilippKhan, MushfiquddinVon, Lindern MariekeKhan, NafeesVon, Lintig JohannesKhan, Nauman RahimVona, RosaKhan, RajwaliVooijs, Marc A. G. G.Khan, RehanVorkas, Charles KyriakosKhan, SaadUllahVorndran, ElkeKhan, SujoyVoronkov, Alexander S.Khanday, Firdous A.Voss, RandalKhanh, Le Nguyen QuocVrecl, MilkaKharaghani, DavoodVriend, JerryKharel, PrakashVu, TuanKhasawneh, FadiVujicic, MiloradKhatami, MahinVukojević, KatarinaKhattab, MostafaVydra, NataliaKheirollahi, VahidVygodina, Tatiana V.Khodzhaeva, Zul’Fiya SagdullaevnaVyhnánek, TomášKhona, DollyWadowski, Patricia PiaKhoshbouei, HabibehWagh, AdityaKhrapko, KonstantinWaghray, AvinashKhrenova, Maria G.Wagner, LarsKi, Sung-hwanWaguri, SatoshiKiang, Juliann G.Waheed, YasirKiczmer, PawełWählby, CarolinaKido, Mizuho A.Wahli, WalterKienzl, MelanieWake, KoushoKietzmann, ThomasWałajtys-Rode, ElżbietaKiktev, NikolayWalczewska, AnnaKim Lim, Lina HsiuWalendzik, KatarzynaKim, BuyeoWaliszewska-Prosół, MartaKim, Byung EuiWalker, FrancisKim, Byung G.Walker, NykiaKim, Byung SooWall, DonnaKim, ChangsooWallace, NicholasKim, Cheorl-HoWallin, Åsa K.Kim, Chi-JuWaloski, Robert AnnyKim, EllaWalter, FruzsinaKim, Eung-GookWalter, IngridKim, Eun-HeeWalter, JonasKim, Hahn YoungWalter, UlrichKim, Hee JungWalters, Eugene HaydnKim, HeiWan, LeiKim, Heui-SooWan, QianfenKim, HyeoneWan, YanminKim, Hyeong-GeugWanaka, AkioKim, Hyun JoonWanders, AlkwinKim, JaeryungWang, AnyouKim, JaesangWang, ChengKim, Jeong-IlWang, Cheng-HsuKim, Jeong-minWang, Chia-YihKim, JiyeonWang, Chih-KuangKim, Jong-WonWang, Chih-YangKim, KanghwanWang, Chrong-ReenKim, Kyeong-ManWang, ChunxinKim, Kyung WonWang, DiKim, Kyung–MinWang, FengKim, MinsooWang, GangKim, Moon YoungWang, GuozhengKim, NayoungWang, Hai LongKim, Ryong NamWang, HaibinKim, Sally YunsunWang, HailinKim, Sang-MinWang, HaitaoKim, Sang-WeWang, HongKim, SeilWang, HonglinKim, SoochongWang, HongmeiKim, Sung HyunWang, HongminKim, SungeunWang, HuiKim, Sung-EunWang, Hui-Min DavidKim, Sung-HoonWang, JiajiaKim, TeayounWang, JianhongKim, Uh-HyunWang, Jian-YingKim, Yeon-SooWang, Jin’AnKim, Yoon YoungWang, KaiKim, Young HwanWang, KankanKim, Young WooWang, KimberleyKim, YoungjoWang, LeiKim, Yun JinWang, LiKimura, YasutoshiWang, LihuaKinashi, HiroshiWang, LinhuaKino, TabitoWang, MingyiKino-oka, MasahiroWang, PengKinra, ManasWang, PingKirby, Elizabeth DianaWang, Po-HuiKiricsi, MónikaWang, QingdeKirischuk, SergeiWang, RuoxiangKiriyama, YoshimitsuWang, San MingKirkegaard-Klitbo, Ditte MarieWang, Seok MuiKirov, Ilya V.Wang, ShanshanKirshenbaum, LorrieWang, ShaobinKirstein, JanineWang, ShaoxunKirtiklis, LechWang, ShiboKiselev, Ivan S.Wang, Tong-HongKishimoto, JiroWang, Wang-XiaKisková, TeréziaWang, WeidongKiss, AlexiWang, WenjunKissoudis, ChristosWang, XiaoyanKitagawa, MunenoriWang, XingKitamura, KatsuyaWang, YajingKitamura, KenjiWang, YanKitaura, JiroWang, YanchangKiyan, YuliaWang, YanchaoKjaer, LasseWang, Yang-KaoKlaassen, IngeborgWang, Yan-HsiungKlabukov, IlyaWang, YigangKlar, AgnesWang, YingKlassen, GiseliWang, Yuan-HungKlassen, RolandWang, Yu-ChiehKlatte-Schulz, FrankaWang, YuedanKlauber-DeMore, NancyWang, YueqiKlein, Peter S.Wang, Zhao V.Klein, SebastianWang, ZhenboKleindienst, AndreaWang, ZhengKlempin, FriederikeWang, ZhixiangKlettner, Alexa KarinaWang, ZongweiKleuser, BurkhardWankhade, Umesh D.Klimczak, AleksandraWant, MuzamilKlimek, KatarzynaWarang, PrashantKlinakis, ApostolosWarfel, Noel AndrewKlose, Christoph S. N.Waryha, Ali MohammadKloss-Brandstätter, AnitaWarzecha, ZygmuntKlotzsch, EnricoWąsik, AgnieszkaKluger, Petra J.Wasilewicz, Michał P.Klymiuk, IngeborgWasson, Christopher W.Knabbe, CorneliusWatała, CezaryKnecht, HansWatanabe, ShigeruKnights, Alexander J.Watanabe, TakashiKnopf, JasminWaters, Samuel T.Knudsen, LarsWatt, PeterKo, HanseokWatts, Phillip C.Ko, Jung-JaeWawrocki, SebastianKobarg, JörgWebber, Lisa J.Kobayashi, HatasuWeber, BernhardKobayashi, JunyaWeber, Jonasz JeremiaszKobayashi, MasakiWeber, K. ScottKobayashi, MichihiroWeber, Yvonne G.Kobayashi, SatoruWehrens, XanderKobayashi, YoshiroWei, ChengguoKobeissy, FirasWei, JunKobori, TakuroWei, Kai-CheKocerha, JannetWei, QiangKoch, Karl-WilhelmWei, WeiKociok, NorbertWei, Wei-ChyouKocki, TomaszWeick, Jason P.Kodani, AndrewWeigmann, BennoKodrik, DaliborWeindl, GüntherKoehne, TillWeinstein-Oppenheime, CarolineKöffel, RenéWeinzierl, RobertKöhler, NatalieWeiskirchen, RalfKoike, AtsushiWeissert, RobertKoike, HarukiWeisz, AlessandroKoirala, DeepakWeitzel, Joachim M.Koistinen, HeikkiWen, HaitaoKokai, Lauren E.Wen, Jake JianjunKokaia, ZaalWen, WeiKokkotou, EfiWenderfer, Scott E.Kokudo, TakashiWender-Ozegowska, EwaKolarič, AnjaWeng, Hao-JuiKölbel, HeikeWeng, XiuhuaKolberg, Hans-ChristianWensel, TheodoreKole, ChristoWenzel, JanKolesnikova, Tatyana D.Werner, Felix MartinKologrivova, IrinaWertel, IwonaKolosnjaj-Tabi, JelenaWesolowski, RobertKoltai, HinanitWestenskow, Peter D.Komatsu, MasanobuWestmark, CaraKomatsu, SatoshiWestwell, AndrewKomleva, Yulia K.Wettervik, Teodor M.Komnenov, DraganaWetzker, ReinhardKomori, KojiWheeler, MichaelKonark, MukherjeeWhisenant, Thomas C.Koncz, IstvánWhite, AnnKondo, MotonariWhite, Kenneth N.Kondo, TomoWhite, ThomasKong, Sun-YoungWicky, ChantalKonishi, MasaakiWidelitz, Randall BruceKonishi, TomokazuWidiapradja, AlexanderKonjar, ŠpelaWiechec, EmiliaKonopelski, PiotrWieczorek, MarekKonrad, FranziskaWiedlocha, AntoniKonstantin, V. SlavinWieers, GregoireKonstantinidis, Theocharis G.Wiegmans, Adrian P.Kontarakis, ZachariasWieland, ThomasKontek, BogdanWielockx, BenKontos, Christos K.Wiemann, StefaKooijmans, SanderWienands, JürgenKoopman, GerritWiener, ReuvenKopeva, KristinaWietrzyk, JoannaKopp, ReinhardWiggs, MichaelKorać, AleksandraWijesinghe, RuchireKorac, BatoWiklander, OscarKorać, PetraWiktorska, KatarzynaKorbecki, JanWilber, Andrew C.Kordes, ClausWilde, AnnegretKorff, ThomasWilhelm, KerstinKorkmaz, CengizWilhelmsson, UlrikaKorkmaz, YükselWillets, JonathonKorolenko, Tatiana A.Williams, E. MarkKoroleva, Elena A.Williams, Jessica A.Koroleva, Iuliia A.Williams, PhilipKoromilas, Antonis E.Williamson, VernellKorzhevskii, DmitriiWillison, Alice G.Kosan, ChristianWilm, BettinaKoskinas, JohnWilmes, AnjaKoskinen, PäiviWilson, Melissa LeeKosmalski, MarcinWindhorst, SabineKosová, KláraWingren, Anette GjörloffKöster, DariusWinning, SandraKostka, TomaszWin-Shwe, Tin-TinKostrominova, TatianaWinter, JochenKostrzewa, MagdalenaWisłowska-Stanek, AleksandraKostyuchenko, RomanWitczak, Carol A.Kostyuk, George P.Witke, WalterKosuge, YasuhiroWitkowska, DanutaKotanidou, AnastasiaWitkowska-Piłaszewicz, OlgaKotelevtsev, Yuri V.Witkowski, WojciechKothari, AshishWittwer, NicoleKotsinas, AthanassiosWłoga, DorotaKotsou, MariaWnuk, AgnieszkaKotula-Balak, MałgorzataWnuk, DawidKou, XiaoxingWöber, ChristianKoua, DominiqueWojakowska, AnnaKoul, BhupendraWojciechowska, MałgorzataKourtidis, AntonisWojcik, AndrzejKoustas, EvangelosWojda, IwonaKoutelou, EvangeliaWołczyński, SławomirKoutroumpakis, EfstratiosWolf, HansKouwenhoven, Mathilde C. M.Wolf, Steven E.Kovács, TiborWölfle, UteKoval, AlexeyWolska, NinaKovalchuk, AlexanderWoltering, ErnstKovalenko, Elena I.Won, Sae-YeonKovalski, JoannaWong, Chi-MingKovalszky, IlonaWong, Ching-OnKowalczyk, PawełWong, ChunKowald, AxelWong, Clarence T. T.Kowalik, MagdalenaWong, GarryKowalski, TadeuszWong, Hovy Ho-WaiKoyama, YutakaWong, JudyKozieł, EdmundWong, Richard W.Koziol-White, Cynthia J.Wong, Siu Hong DexterKozlov, AndreyWong, Wing Tak JackKozlov, SergeyWoo, So-YounKozłowska, AnnaWood, TimKozłowska, MariolaWooldridge, Amy L.Kozma-Bognár, LászlóWorthmann, AnnaKozubowski, LukaszWright, Gus A.Kraaij, RobertWright, Helen L.Kraft, RobertWrobel, LidiaKraft, Timothy W.Wrotek, SylwiaKrag, Thomas. O.Wrześniok, DorotaKrantic, SlavicaWu, BiKrasemann, SusanneWu, BinKraskiewicz, HonorataWu, Bin-NanKraus, TheoWu, Cheng-TienKravchik, MichaelWu, Chun-ChiKrawczenko, AgnieszkaWu, DanKrebs, JoachimWu, DiKreis, StephanieWu, Guan-ChungKrejsgaard, ThorbjørnWu, GuangyuKretowski, RafalWu, HaichongKretsovali, AndronikiWu, HuaizhuKreutzberger, Alex J.Wu, Hua-TaoKreutzmann, Judith C.Wu, JianKrezdorn, NiccoWu, JianguoKrieger, José E.Wu, JunfangKrieghoff-Henning, EvaWu, KailiKrijgsman, DaniëlleWu, Katherine C.Krishnan, HariniWu, KeqiangKrishnan, ShanmugarajanWu, KeyangKristensen, SalomeWu, Li-chenKristian, TiborWu, LicunKrizmanić, JelenaWu, Li-WhaKroeker, AndreaWu, LongjunKroenke, Christopher D.Wu, MengKroken, Abby R.Wu, MinKronenberg, DanielWu, PeiyiKronsteiner, BarbaraWu, Shan-YingKruglov, AlexeyWu, ShibeiKrum, Susan A.Wu, SiqiKrzyżanowska-Gołąb, DorotaWu, WenxinKshirsagar, SudhirWu, WutianKu, RayWu, XiaokangKuan, Yu-HsiangWu, XinchenKuban-Jankowska, AlicjaWu, XingyuKubo, YoshinaoWu, YahongKubo, YoshiyukiWu, YihuiKuboki, TakuoWu, YuanhuaKubota, KeiichiWu, YuliangKucharczyk, DariuszWuest, StephanKuchler-Bopp, SabineWüestefeld, TorstenKuçi, SelimWullaert, AndyKucic, NataliaWurm, Florian M.Kučinskas, VaidutisXi, JiajiaKudova, EvaXi, LeiKudryashov, Dmitri S.Xia, JihanKudryashova, ElenaXia, XuhuaKuek, VincentXiang, WeiKuijpers, TacoXiao, HaipengKujan, OmarXiao, JunKukor, ZoltánXiao, LanKulbacka, JulitaXiao, LiKulisz, JoannaXiao, WeikunKulka, MariannaXiao, YiKulkarni, AbhishekXie, CanKulkarni, ArpitaXie, ChaoKumamoto, EiichiXie, HehuangKumar Jha, MithileshXie, JindongKumar, AmitXie, MinKumar, AnilXie, QingmeiKumar, AnoopXie, RuosenKumar, AnujXifró, XavierKumar, ArvindXinaris, ChristodoulosKumar, AshishXiong, JingweiKumar, AshokXirodimas, DmitrisKumar, AshutoshXu, ChuanmingKumar, AvinashXu, DehuiKumar, BalawantXu, DejunKumar, Bhuvnesh SunilXu, DongboKumar, HemantXu, EnquanKumar, JitendraXu, FengKumar, K. J. SenthilXu, GuozhouKumar, Kishore RajXu, HuafengKumar, ManuXu, JianKumar, Nagi B.Xu, JianzhenKumar, PavanXu, JieKumar, PraveenXu, JingliangKumar, RahulXu, JunKumar, RaviXu, KangKumar, RavinderXu, MeifengKumar, RohitXu, NaihanKumar, SachinXu, PengfeiKumar, SatwantXu, QinKumar, ShaileshXu, TaoKumar, SugandhXu, WeiKumar, SusheelXu, XinKumar, T. K. S.Xu, XuejiaoKumar, TanweerXu, YanKumar, UjendraXu, ZhihengKumar, VijayXuan Hieu, CaoKumar, VikasXue, MengzhouKumaraswami, KondaXu-Monette, Zijun YidanKumari, DamanYabuki, ShojiKunacheewa, ChutimaYadav, DhananjayKunc, MichałYadav, JayKuncha, Santosh KumarYadav, RajKundu, AmitYadav, Santosh K.Kundu, ParagYadav, VikasKung, SamYahubyan, Galina T.Kung, Woon-ManYakovlev, AlexanderKunimatsu, RyoYamad, TaketoKunz, WolframYamagata, MasahitoKuo, Chan-YenYamaguchi, SatoshiKuo, Hsiao-HsuanYamaguchi, YoshikiKuo, Jinn-RungYamamoto, FumiichiroKuo, Sung-HsinYamamoto, NaokiKuo, Yen-WenYamamoto, YusukeKura, BranislavYamaoka, ToshimitsuKurasaki, MasaakiYamasaki, HideoKurokawa, JunkoYamashiro, SawakoKurokawa, MasaomiYamashita, HirokoKurose, HitoshiYamashita, HironobuKurowski, MarcinYamashita, KimihiroKurzbach, AnizaYamauchi, TakahiroKusnierczyk, PiotrYamauchi, YoheiKusumoto, DaiYamazaki, FumikazuKutikhin, Anton G.Yamazaki, ManabuKuzuya, TeijiYan, GegeKweon, Oh-KyeongYan, JiangweiKwiatkowska, KatarzynaYan, PengzeKwiecien, IngaYan, RuiKwon, InsuYan, WeiKwon, SoYan, Yong-BinKwong, JosephYanagisawa, HiromiLa Monica, SilviaYang, BoguangLa, Penna GiovanniYang, Chao-YieLabadie, ThomasYang, Chien-ChungLabahn, JoergYang, ChuanbinLabardi, MassimilianoYang, Chuen-MaoLabeit, SiegfriedYang, ChunguangLabhasetwar, VinodYang, CindyLabour, Marie-NoëlleYang, Deng-HoLabrie, MarilyneYang, FengLachke, SalilYang, GuanLachmann, NicoYang, Guo-YuanLacina, LukasYang, HaopengLaConte, Leslie E.Yang, HongLaffolie, Jan DeYang, HuaqiangLaFlamme, Susan E.Yang, Huaxiao AdamLafont, ElodieYang, Hung-ChihLafont, VirginieYang, KunLafrenaye, AudreyYang, LiliLagneaux, LaurenceYang, LingLagranha, Cláudia JacquesYang, LiweiLahm, HaraldYang, MengbiLahuerta, Juan JoséYang, MingLai, FlorenceYang, MingchongLai, MicheleYang, QiweiLai, PuxiangYang, ShengLaight, DavidYang, SongLaird, AngelaYang, TaoLaiva, AshangYang, WeiLaizé, VincentYang, Wei-hsiungLajqi, TrimYang, Wen-HaoLakey, JeremyYang, XiLakić, IvaYang, XingLallemand, FraņcoisYang, XiuyingLam, YanYang, XuesongLamar, John M.Yang, Ya-LingLamartine, JérômeYang, YangLamas, AlexandreYang, Yi-QingLamba, DorianoYang, YongLambert, JohnYang, ZhihongLamberti, MaríaYao, JianboLammerding, JanYao, LijunLammert, EckhardYao, ShaomianLammi, Mikko J.Yao, ShenqinLampinen, MariaYao, XiaoqiangLamport, Derek T. A.Yap, Wei BoonLamprecht, RaphaelYarım, Gül FatmaLamprou, MargaritaYarygin, Konstantin N.Landi, ClaudiaYasuda, YutoLandi, SilviaYasufumi, KatanasakaLandim-, Alvarenga FernandaYasuhara, NorikoLandowski, MichaelYaswanth, KuthatiLane, MichaelYazawa, TakashiLaneri, SoniaYazdanian, MohsenLaney, Dawn A.Yazdian, FatemehLanfranchi, GerolamoYdyrys, AlibekLang, LiweiYe, GangLang, MorinYe, ZhennanLang, RolandYeh, I-JuLangdon, CaseyYeh, Jiann-HorngLange, FalkoYeh, Jwu-LaiLange-Consiglio, AnnaYelamanchili, Sowmya V.Languino, Lucia R.Yélamos, JoséLanna, CaterinaYeng, Looi ChungLannoy, DamienYeo, SujungLanz, RainerYesilkanal, Ali EkremLaprévôte, OlivierYi, Eunhee S.Lara, LoboYi, Yong WeonLarocca, Maria CeciliaYildiz, AysegulLaRocque, JanYin, LiyaLaronda, Monica M.Yin, TaoLarre, IsabelYin, WeiyanLarrivée, BrunoYochum, GregoryLarsen, PeterYoder, MervinLaselva, OnofrioYoke, Kqueen CheahLashin, Sergey A.Yokota, TakafumiLaskowska, MarzenaYoon, Je-HyunLasoń, WładysławYoshida, KatsunoriLatina, ValentinaYoshida, ManabuLatire, ThomasYoshimura, MasamiLatorre, EvaYoshino, HironoriLattante, SerenaYoshino, YukiLattanzi, GiovannaYoshioka, KentaroLattanzi, WandaYosipovitch, GilLatteri, SaverioYou, SixianLau, AndyYou, XiaonaLauriano, Eugenia RitaYou, Zhu-HongLauzon-Joset, Jean-FrancoisYouker, RobertLauzurica, PilarYoum, Jae BoumLavender, AndrewYoun, Hyun JoLaverny, GillesYoun, Seock-WonLaviola, GiovanniYounis, Nancy S.Lavogina, DarjaYousef, EinasLavrik, OlgaYousefi, Seyede RahelehLaw, John LokYousefzadeh, MatthewLawal, Ismaheel O.Yu, BoLawrence, B.Yu, Hong-RenLawson, CharlotteYu, Huang-PingLayek, BuddhadevYu, HuiLazarevic, MilosYu, JiaLázaro-Diéguez, FranciscoYu, JialiLazennec, GwendalYu, JiaquanLazrak, AhmedYu, JunLazzarini, EdoardoYu, KaiLe Romancer, MurielYu, NingLe, Dévédec SylviaYu, PengchunLe, WeidongYu, QuanyouLeastro, Mikhail OliveiraYu, RenqiangLeBeau, Fiona E.Yu, ShanpingLebedeva, OlgaYu, ShuangLebrilla, Carlito B.Yu, TengtengLechniak, DorotaYu, YueLeclerc, EstelleYu, Yung-LuenLeclercq, IsabelleYu, ZuorenLecureur, ValérieYuan, JianyeLedesma, Colunga MaríaYuan, XimingLee, Byeong HaYuan, Zi-GuoLee, CharlesYudoh, KazuoLee, Chien-HsingYuksel, SelcukLee, Chul-HwanYuldasheva, Nadira Y.Lee, DeokhoYun, Bo HyonLee, Dong HunYurochko, AndrewLee, DongheonYusof, Siti RafidahLee, Dong-SeolYuspa, Stuart H.Lee, HaroldYusuke, ImagawaLee, Hsin-ChenYu-Taeger, LiboLee, HyeminYuyama, KoheiLee, Hyun SooYuzbasiyan-Gurkan, VilmaLee, HyunjuYuzhalin, Arseniy E.Lee, J. ScottZaarour, Rania FaouziLee, JaewonZabel, Brian A.Lee, Jana C.Zabuchi, GiulianoLee, Jeong-ChaeZaccagnini, GermanaLee, JeungchanZaccara, SaraLee, Jong-hanZacco, ElsaLee, JungmyungZacharoulis, DimitrisLee, Keun SeokZachos, GeorgeLee, Ki YoungZada, SahibLee, Kuan-HanZádor, ErnöLee, Michael K.Zafeiriou, Maria PatapiaLee, Min YoungZaffaroni, NadiaLee, MyoungsookZagorac, SladjanaLee, Peter Chang-WhanZagorchev, LyubenLee, Robert J.Zagrebelsky, MartaLee, Sang JoonZahan, MariusLee, Seung KyuZaharie, Gabriela C.Lee, Sung JoongZahid, SaadiaLee, Sung-BauZahler, StefanLee, SunghwanZaiou, MohamedLee, Tsung-HsienZaiss, DietmarLee, Tzong-ShyuanZaitone, Sawsan A.Lee, Wei-HwaZakaria, DaliaLee, Wing KeeZakharov, YuryLee, Wing-HinZakharova, Ekaterina V.Lee, Yen-ChienZakkar, MustafaLee, Yi-JangZaldumbide, ArnaudLee, YoungkyunZaman, Qamar U.Lee, Yun-SilZaman, WajidLee, YureeZampino, MartaLee-Liu, DasfneZanetti-Domingues, Laura CarolinaLee-Ruff, EdwardZangle, ThomasLefort, Craig T.Zani, Bianca MariaLegartová, SoňaZanni, GinevraLegay, ClaireZapico, Sara C.Legembre, PatrickZaravinos, ApostolosLeggatt, GrahamZaric, Kontic MarinaLegland, DavidZarnitsyna, Veronika I.Legler, Daniel F.Zarogiannis, SotiriosLehmann, ChristianZatsepin, TimofeiLehmann, JörgZatsepina, Olga G.Lei, MingxingZavala-Cerna, MariaLei, Tie-ChiZavattaro, ElisaLei, WeiZavros, YanaLei, ZhiyongZawadzka, MalgorzataLei, Zi-NingZayed, MohammedLeisengang, StephanZaza, AntonioLeisz, SandraŻebrowska-Nawrocka, MartaLejeune, FabriceZegeye, Mulugeta M.Lemaître, GillesZeier, ZaneLemay, GuyZeitz, Michael J.Leme, Ligiane DeŻelechowska, PaulinaLemonnier, LoicZelek-Molik, AgnieszkaLeng, RogerZella, DavideLeng, TiandongZellner, MariaLenka, NibeditaZellner, Wendy L.Lennartz, MichelleZemła, JoannaLenos, Kristiaan J.Zenaro, ElenaLents, ClayZeng, JipingLenzi, PaolaZeng, LiLeoncini, LorenzoZeng, SicongLeoncini, SilviaZeng, WeifengLeone, PatriziaZeng, YongjiLeoni, ValerioŽenka, JanLeon-Juarez, MoisesZentilin, LorenaLeow, Melvin K. S.Zepeda, AngelicaLeow, Melvin Khee ShingZeshan, BasitLepper, ChristophZeviani, MassimoLeppik, LiudmilaZgliczynska, MagdalenaLerardi, EnzoZhabyeyev, PavelLeri, ManuelaZhai, Rong GraceLes, FranciscoZhan, TianzuoLeshkowitz, DenaZhang, AidiLeskovac, Andreja R.Zhang, BohanLeśniak, WieslawaZhang, ChaoLeśnikowski, Zbigniew JanZhang, ChengchengLettau, MarcusZhang, ChengxinLeu, Jia-ShiunZhang, ChuanhaiLeung, Anthony K. L.Zhang, DalinLevada, KaterynaZhang, Daniel XinLevander, FredrikZhang, DianbaoLevens, David L.Zhang, DuanwuLevone, Brunno RochaZhang, DuoLevy, EfratZhang, EnxiangLewandowska-Szumieł, MałgorzataZhang, ErshuaiLewinska, AnnaZhang, FanLewis, L. KevinZhang, FengLewko, BarbaraZhang, GaoLey, KlausZhang, HaoLeyva, KathrynZhang, HaomingLi Puma, Domenica DonatellaZhang, HuiliangLi, AngZhang, HuizhenLi, BinsenZhang, IssanLi, ChangZhang, JianhuaLi, Chuan-YuanZhang, JianingLi, ChunyingZhang, JianminLi, FangZhang, Jian-YeLi, FengzhiZhang, JiaxingLi, FuyiZhang, JieLi, GuangzhaoZhang, JileiLi, HongjunZhang, JunLi, HuiliangZhang, KeqiangLi, HuixiaZhang, KuiLi, JianZhang, LeiLi, JianrongZhang, LiningLi, JinanZhang, MingLi, JingyaZhang, MingyangLi, JinhuiZhang, MinjieLi, JinlinZhang, PengLi, Jin-luanZhang, QiangLi, JinyuZhang, QiaoLi, JunZhang, RanLi, JunhaoZhang, RudianLi, KaZhang, ShijunLi, KeZhang, ShubingLi, LiZhang, SouquanLi, Li-FuZhang, TiantianLi, LiliZhang, TingxinLi, LingZhang, WeiLi, LiyaZhang, WenboLi, MiZhang, WenjunLi, MinZhang, XiaonanLi, PengZhang, XiaoyuLi, QianZhang, XinLi, QiuZhang, XingLi, QiubaiZhang, XuemingLi, Ruei-NianZhang, XuesongLi, RuiZhang, YanLi, TangliangZhang, YingzeLi, TiangangZhang, YinhuaLi, WeiZhang, YongLi, XiaofeiZhang, YuLi, XiaojiangZhang, YunwuLi, XiaopengZhang, YunxiaoLi, XingangZhang, Zhentao (China)Li, XinleiZhang, Zhentao (USA)Li, YafengZhang, ZhibingLi, YanZhang, ZhiguoLi, YaoZhang, ZuohuiLi, YichaoZhao, BiLi, YingchuanZhao, ChiLi, YingxiangZhao, JianpingLi, YongguoZhao, JingLi, YulongZhao, LiangLi, YunleiZhao, RunchenLi, YuxinZhao, ShidouLi, ZheqiZhao, SiyuanLi, ZhongZhao, WuliLi, ZhonghanZhao, XiaodiLi, ZhuqingZhao, XiaohangLi, ZicongZhao, XiaonanLi, Zi-JianZhao, YangLian, QizhouZhao, YongLiang, GongZhao, YuguangLiang, ShunqingZhao, YunfengLiao, ChangZhao, ZongshengLiao, PengZheng, BinhaiLiao, WupengZheng, DaliLiao, Yi-JenZheng, GuopingLiao, ZehuanZheng, JialinLiarte, SergioZheng, LinglingLibardi, Cleiton AugustoZheng, PengyuanLibra, MassimoZheng, PingLiby, KarenZheng, ShaoquanLichtstein, DavidZheng, TianyuLicini, CaterinaZheng, Wen-HuaLiebau, StefanZheng, YeLieder, HelmutZheng, Yun-WenLiefke, RobertZheng, ZizhengLien, YuchinZhong, XiaolingLiese, JulianeZhou, Guang-BiaoLightfoot, Adam P.Zhou, JingsongLill, Christina M.Zhou, JingyingLilly, Michael B.Zhou, LiangLim, DmitryZhou, LibinLim, FilipZhou, LufangLim, HanZhou, PengLim, Jeong-HoonZhou, PingLim, Kwang SukZhou, PingkunLim, SoyeonZhou, QingyuLim, Tae-GyuZhou, QunLim, WonbongZhou, ShuanhuLim, WonchungZhou, SitongLima, Joanna DarckZhou, XiangmeiLima, Maria HelenaZhou, XiaolingLimanaqi, FionaZhou, YimingLimjunyawong, NathachitZhou, ZhidongLimon, AgenorZhou, ZhongwuLin, Chang-ChiZhu, BangfuLin, Chien-MinZhu, BaojianLin, ChinghsiungZhu, FangLin, ChujiaoZhu, Guo-ZhangLin, Dar-ShongZhu, JiefuLin, Hsi-HsienZhu, JoeLin, Huey-JenZhu, KongjuLin, Hung-YuZhu, LingLin, JianZhu, MengLin, Julie QiaojinZhu, Michael X.Lin, LiZhu, ShizhenLin, LianbingZhu, SiboLin, WeichunZhu, SongliLin, XialuZhu, WuqiangLin, XiaoZhu, ZhichuanLin, Yi-YuanZhuang, JunlingLin, ZhichengZhuo, YehongLinder, BenediktZieba-Przybylska, DorotaLindinger, MichaelZiemnicka, KatarzynaLindner, RobertZienkiewicz, KrzysztofLindquist, Jonathan A.Ziganshin, AyratLindqvist, JohanZíková, MartinaLindsay, Susan L.Zimmer-Bensch, GeraldineLindstedt, SandraZimmermann, RichardLindström, Mikael S.Ziółkowska, BarbaraLink, WolfgangZiolkowska, NataliaLino, Miguel M. F.Zissel, GernotLio, DomenicoZissler, UlrichLiobikas, JuliusZito, FrancescaLiovic, MirjanaZivkovic, AngelaLippert, EricZlatina, KristinaLips, Katrin SusanneZmijewska, AgataLira-Medeiros, CatarinaZmijewski, MichalLirussi, FrédéricZmonarski, SławomirLisa, AndreaZobi, FabioLisignoli, GinaZoccali, GiovanniLisowska, BarbaraZohdi, HamoonLisowska, Katarzyna M.Zoidl, GeorgListos, JoannaZoladek, TeresaLitowczenko, JagodaZolfaghari Emameh, RezaLittle, Joshua W.Zolkowska, DorotaLitwa, Karen A.Zommiti, MohamedLiu, AipingZong, Wei-XingLiu, BoZoratti, MarioLiu, ChangmeiZorkina, YanaLiu, ChengfeiZorov, Dmitry B.Liu, ChunZou, XuenongLiu, Daniel S. K.Zubareva, Ekaterina V.Liu, DongŻuchowska, AgnieszkaLiu, DongfangZuellig, RichardLiu, EnwuZuena, Anna RitaLiu, JianxinZühlke, DanielaLiu, JieZúñiga-Pflücker, JuanLiu, JingZununi-vahed, SepidehLiu, JingyuŻurawek, MagdalenaLiu, Jung-PingŻurek, GrzegorzLiu, LaikuiZurita, MarioLiu, Li-PingZusterzeel, PetraLiu, LiuZvyagilskaya, Renata A.Liu, LixinZweigerdt, RobertLiu, LumeiZylinska, Ludmila


